# Solvent Drives Switching between Λ and Δ Metal Center Stereochemistry of M_8_L_6_ Cubic Cages

**DOI:** 10.1021/jacs.2c00245

**Published:** 2022-04-02

**Authors:** Weichao Xue, Tanya K. Ronson, Zifei Lu, Jonathan R. Nitschke

**Affiliations:** Yusuf Hamied Department of Chemistry, University of Cambridge, Cambridge CB2 1EW, United Kingdom

## Abstract

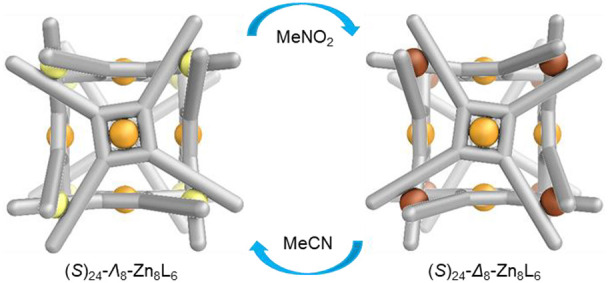

An enantiopure ligand with four bidentate metal-binding sites and four (*S*)-carbon stereocenters self-assembles with octahedral Zn^II^ or Co^II^ to produce *O*-symmetric M_8_L_6_ coordination cages. The Λ- or Δ-handedness of the metal centers forming the corners of these cages is determined by the solvent environment: the same (*S*)-ligand produces one diastereomer, (*S*)_24_-Λ_8_-M_8_L_6_, in acetonitrile but another with opposite metal-center handedness, (*S*)_24_-Δ_8_-M_8_L_6_, in nitromethane. Van ’t Hoff analysis revealed the Δ stereochemical configuration to be entropically favored but enthalpically disfavored, consistent with a loosening of the coordination sphere and an increase in conformational freedom following Λ-to-Δ transition. The binding of 4,4′-dipyridyl naphthalenediimide and tetrapyridyl Zn-porphyrin guests did not interfere with the solvent-driven stereoselectivity of self-assembly, suggesting applications where either a Λ- or Δ-handed framework may enable chiral separations or catalysis.

The chirality of metal–organic cages has enabled novel applications across different areas.^1^ For instance, the stereochemistry of cages has been used to recognize and separate enantiomers from racemic mixtures through encapsulation or cocrystallization.^2^ Enantiopure cages are also able to mimic the catalytic functions of enzymes,^3,4^ promoting asymmetric transformations by shaping the chirotopic space around reactive intermediates.^5^ Recently, chiral cages that emit circularly polarized luminescence (CPL) have also emerged as a novel platform for the modular design of CPL-active materials,^6^ which are of potential use in optical information transfer and new display technologies.^7^

Different methods can be used to prepare enantiopure cages. One approach is the resolution of racemic cage mixtures,^8^ which can be induced by chiral guests.^9^ Alternatively, the direct self-assembly of enantiopure ligands around metal ions can produce stereochemically pure cages.^1^ This method involves stereochemical information transfer from ligands to metal centers, which adopt preferentially a Δ or Λ configuration based upon ligand sterics.^10^ As a consequence, coordination cages are formed in a diastereoselective manner. Among these enantiopure architectures are sandwiches,^11^ helicates,^12^ tetrahedra,^2e,4e,13^ octahedra,^14^ cubes,^15^ knots,^16^ and other structures.^1,17^

In cases that have been reported so far, one enantiomer of ligand leads to a single diastereomer of metal–organic assembly, with diastereoselective self-assembly minimally impacted by external factors.^18^ Here we demonstrate the self-assembly of a single enantiopure ligand with octahedral metal ions to produce two distinct diastereomers of an M^II^_8_L_6_ cage, having metal centers with either a preferred Δ or Λ configuration, through a simple change of solvent.

Enantiopure porphyrin-containing fourfold-symmetric ligand **A** (Figure 1a) was synthesized from commercially available 5,10,15,20-tetrakis(pentafluorophenyl) porphyrin^19^ as described in Supporting Information section S2. The amide-containing chiral directing groups, each bearing a carbon stereocenter with an a (*S*) configuration, were incorporated near the coordination sites. Based upon the original work of Lusby *et al*. on cages built from pyridyl-triazole-based ligands,^20^ we envisioned that this design, together with the high rigidity of the porphyrin skeleton, would influence the metal center configurations during cage formation, resulting in diastereoselective cage formation.

**Figure 1:**
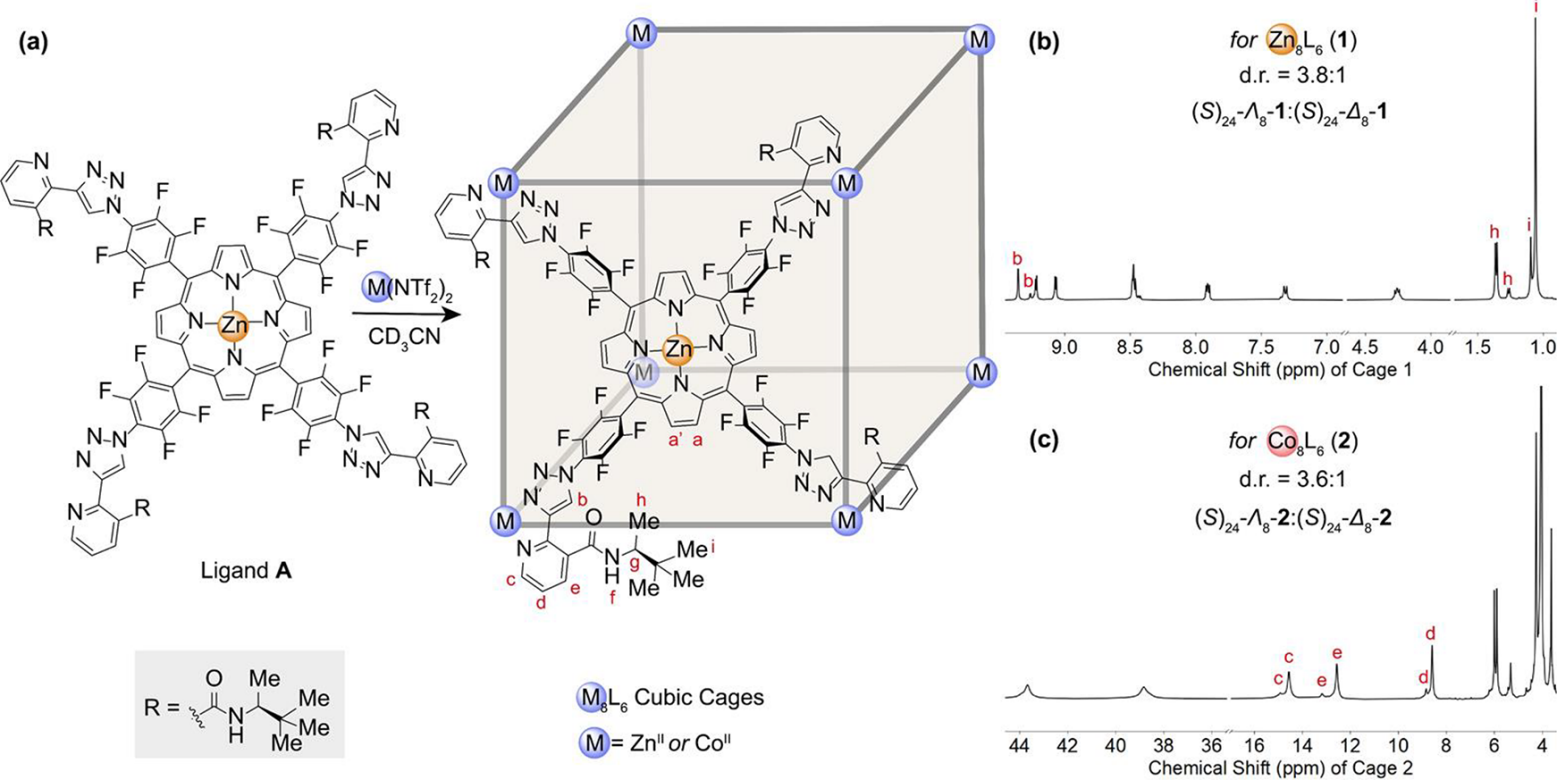
(a) Diastereoselective self-assembly of Zn^II^_8_L_6_ and Co^II^_8_L_6_ cages **1** and **2** from chiral porphyrin ligand **A**. The chiral directing group R is highlighted in the gray box. (b) Partial ^1^H NMR spectrum of Zn^II^_8_L_6_ cage **1** (CD_3_CN, 500 MHz, 25 °C). (c) Partial wide-sweep ^1^H NMR spectrum of Co^II^_8_L_6_ cage **2** (CD_3_CN, 500 MHz, 25 °C).

The self-assembly of **A** (6 equiv) with zinc(II) bis(trifluoromethanesulfonyl)imide (Zn(NTf_2_)_2_, 8 equiv) in acetonitrile at 70 °C produced Zn_8_L_6_ cubic cage **1** as the uniquely observed product, with the metal centers adopting either all Δ- or all Λ-handedness (Figure 1a). Electrospray ionization mass spectrometry (ESI-MS) confirmed formation of a Zn^II^_8_L_6_ complex (Figure S18). Two sets of H_b_, H_h_, and H_i_ signals in the ^1^H NMR spectrum indicated that the cage consists of a pair of diastereomers. The well-separated signals allowed determination of the diastereomeric ratio (d.r.) to be 3.8:1 (Figure 1b). Diffusion-ordered spectroscopy (DOSY) NMR experiments confirmed the same diffusion coefficient for both diastereomers (Figure S14). In control experiments, a monomeric pyridyl-triazole ligand bearing the same chiral directing group reacted with Zn(NTf_2_)_2_ (Figures S77 and S78), forming a ZnL_3_ complex with significantly lower diastereoselectivity (d.r. = 1.3:1). These results indicated that the energy difference between the Zn_8_L_6_ diastereomers emerges as a consequence of higher-order assembly.

To demonstrate the generality of this method, cobalt(II) bis(trifluoromethanesulfonyl)imide was also employed in self-assembly (Figures 1c), leading to the formation of Co^II^_8_L_6_ cubic cage **2** in equally high yield and with similar diastereoselectivity (d.r. = 3.6:1).

Circular dichroism (CD) spectroscopy was used to gauge the diastereoselectivities of formation of zinc and cobalt cages **1** and **2** (Figure S20). Both **1** and **2** displayed clear negative Cotton effects in acetonitrile, with similar intensities in the region from 413 to 440 nm, corresponding to the Soret bands of the Zn-porphyrin walls. In accordance with previous observations^19,21^ and the present NMR spectra (Figures 1 and S22), we inferred that the major diastereomer for each cage has all eight metal centers with either Λ- or Δ-handedness, whereas the minor diastereomer has all metal centers with the opposite stereochemical configuration. Other diastereomers, containing both Λ and Δ metal vertices, were not observed by NMR.

Purple crystals suitable for analysis by single-crystal X-ray diffraction were obtained by slow diffusion of diethyl ether into acetonitrile solutions of the Zn_8_L_6_ and Co_8_L_6_ cages **1** and **2** (Figure 2). The X-ray structures revealed six chiral ligands bridging eight octahedral Zn^II^ or Co^II^ centers in an *O*-symmetric cubic configuration, with all eight stereogenic metal centers adopting a Λ configuration surrounded by three (*S*)-bidentate chelating moieties. Within (*S*)_24_-Λ_8_-**1**, the metal–metal distances are 19.4 Å for Zn^II^ centers forming adjacent vertices and 19.8 Å for the Zn^II^ centers in facing porphyrins. For (*S*)_24_-Λ_8_-**2**, the corresponding Co^II^···Co^II^ and Zn^II^···Zn^II^ distances are 18.6 and 19.7 Å, respectively. The internal cavity volumes of (*S*)_24_-Λ_8_-**1** and (*S*)_24_-Λ_8_-**2** were calculated to be 2881 Å^3^ and 2906 Å^3^ respectively using the MoloVol program (Figure S74).^22,23^

**Figure 2:**
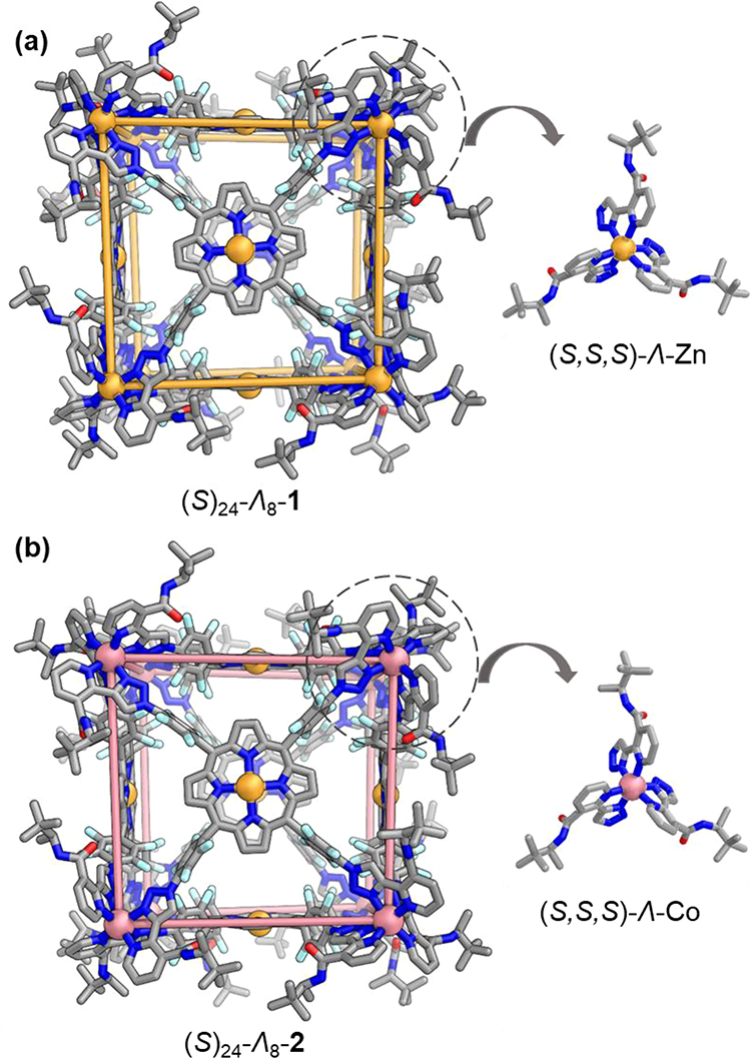
(a) Crystal structure of (*S*)_24_-Λ_8_-Zn_8_L_6_ cage **1**, highlighting a single (*S*,*S*,*S*)-Λ-Zn center. (b) Crystal structure of (*S*)_24_-Λ_8_-Co_8_L_6_ cage **2**, likewise showing a single (*S*,*S*,*S*)-Λ-Co center. Disorder, hydrogen atoms, and H_2_O bound to the porphyrin Zn^II^ centers are omitted for clarity.

We then investigated the parameters that can influence diastereocontrol in the self-assembly of cage **1**. The concentration appeared to not impact the diastereomeric ratio, as the same d.r. of 3.8:1 was observed when the reaction of ligand **A** with Zn(NTf_2_)_2_ was carried out at ligand concentrations ranging from 1 to 8 mM (Figures S40 and S41).

We found the diastereoselectivity of self-assembly to be profoundly influenced by the choice of reaction solvent, however. In acetonitrile, we tentatively assign the major diastereomer of cage **1** as (*S*)_24_-Λ_8_-**1**, based on the observation that this diastereomer crystallized from acetonitrile. This diastereomer was obtained with a d.r. of 3.8:1 in acetonitrile, whereas in nitromethane the diastereomer with opposite metal handedness, (*S*)_24_-Δ_8_-**1**, was formed predominantly, with a d.r. of 1:6 (Figure 3a). Opposite Cotton effects observed in the CD spectra of cage **1** in acetonitrile and nitromethane also confirmed these divergent stereochemical outcomes (Figure 3c). At different ratios of these two solvents (CD_3_CN:CD_3_NO_2_), the diastereomeric ratio of cage **1** also differed (Figure 3b). Although good diastereoselectivity was observed in nitromethane, attempts at growing crystals of (*S*)_24_-Δ_8_-**1** in nitromethane suitable for X-ray diffraction were unsuccessful.

**Figure 3:**
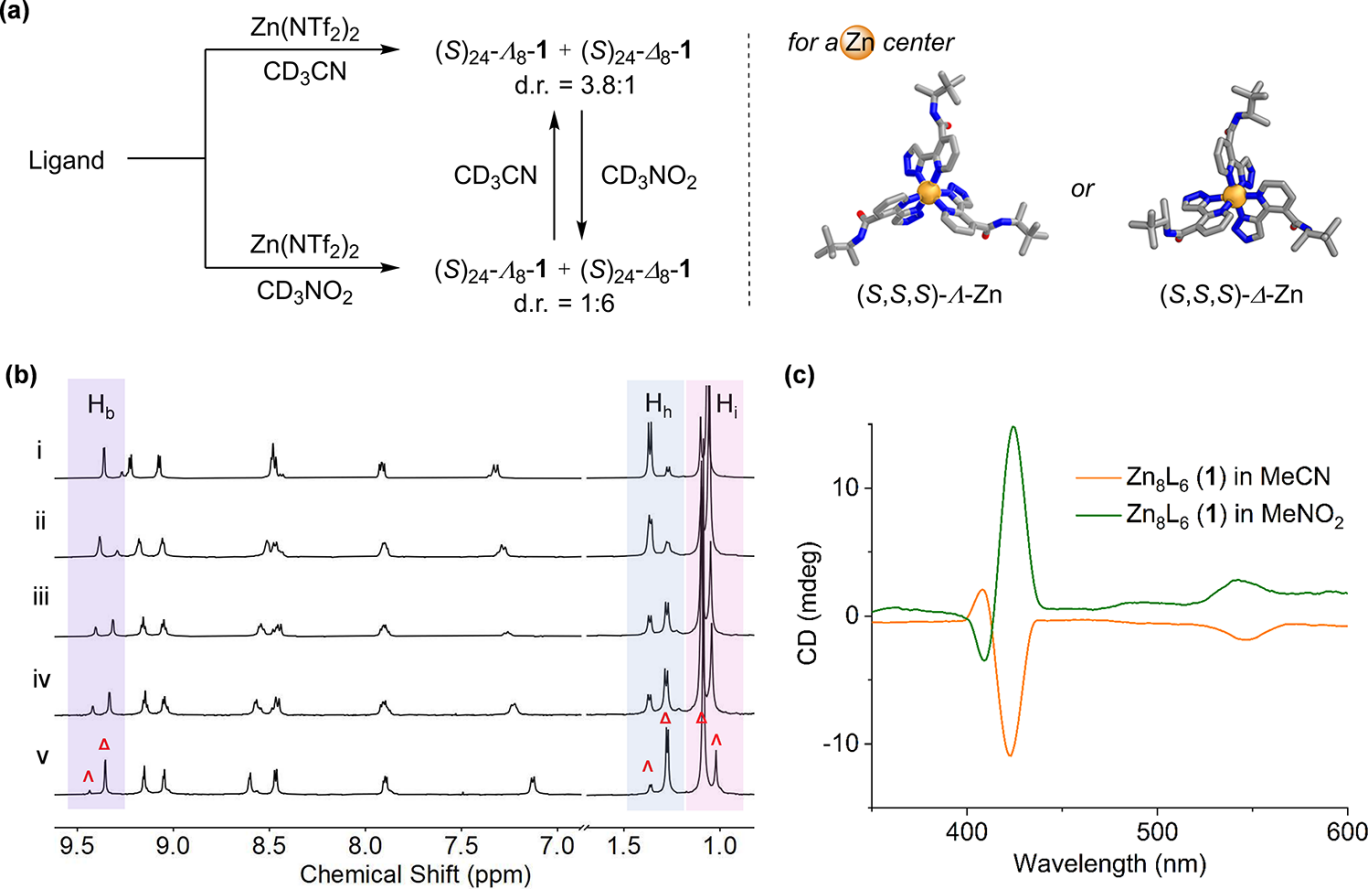
(a) Solvent-dependent self-assembly of Zn_8_L_6_ cage **1** to form two diastereomers (d.r. = (*S*)_24_-Λ_8_-**1**:(*S*)_24_-Δ_8_-**1**). (b) Partial ^1^H NMR spectra (CD_3_CN or CD_3_NO_2_, 500 MHz, 25 °C) of Zn_8_L_6_ in different solvent ratios: (i) CD_3_CN, d.r. = 3.8:1; (ii) CD_3_CN:CD_3_NO_2_ = 7:3, d.r. = 2.2:1; (iii) CD_3_CN:CD_3_NO_2_ = 1:1, d.r. = 1:1.6; (iv) CD_3_CN:CD_3_NO_2_ = 3:7, d.r. = 1:2.6; (v) CD_3_NO_2_, d.r. = 1:6. (c) CD spectra of Zn_8_L_6_ in MeCN and MeNO_2_ at the same concentrations. A PM3 molecular model of a (*S*,*S*,*S*)-Λ-Zn center was minimized using the SCIGRESS software package,^24^ whereas the (*S*, *S*, *S*)-Δ-Zn center is from the crystal structure.

In control experiments, ligand **A** displayed no Cotton effects in either solvent (Figure S10). It was also observed that removing acetonitrile through evaporation, and subsequently adding nitromethane, switched the diastereoselectivity from 3.8:1 to 1:6 after 2 h at 70 °C (Figure S30). Removal of nitromethane and readdition of acetonitrile restored the diastereomeric ratio to 3.8:1 after 10 min at 25 °C. These observations indicated that Δ-Zn ⇄ Λ-Zn interconversion is reversible, with the equilibrium position governed by the solvent. Λ-Zn centers within **1** were thus favored in acetonitrile, whereas Δ-Zn centers were preferred in nitromethane.^25,26^ Predominantly (*S*)_24_-Δ_8_-**1** (d.r. = 1:2.4) was observed to form in acetone (Figure S24), as with nitromethane.

The impact of reaction temperature on diastereocontrol was also examined. The self-assembly of cage **1** was performed at elevated temperatures from 80 to 120 °C. Immediately after cooling to 25 °C, ^1^H NMR spectra were measured. The results showed that the diastereoselectivities remained the same as those of experiments carried out at 70 °C in both acetonitrile (d.r. = 3.8:1) and nitromethane (d.r. = 1:6).

The acetonitrile solution of **1** was stored at 25 °C for 6 months with no changes observed in the diastereomeric ratio. Variable temperature ^1^H NMR experiments indicated minimal temperature-dependent diastereomer interconversion in acetonitrile (Figure S38). In contrast, a temperature-dependent interconversion was observed in the nitromethane solution of **1** (Figure S38). After self-assembly in MeNO_2_, a diastereomeric ratio of (*S*)_24_-Λ_8_-**1**:(*S*)_24_-Δ_8_-**1** = 1:6 was observed. After 7 days at 25 °C, an equilibrium diastereomeric ratio of (*S*)_24_-Λ_8_-**1**:(*S*)_24_-Δ_8_-**1** = 1:1.5 was reached; reequilibration back to the original diastereomeric ratio of 1:6 occurred following heating of the diastereomeric mixture of cages.

Variable temperature ^1^H NMR experiments enabled the construction of a van ’t Hoff plot, which provided thermodynamic insight into the temperature-dependent diastereomer interconversion in nitromethane (Figure S39). The conversion of (*S*)_24_-Λ_8_-**1** into (*S*)_24_-Δ_8_-**1** in nitromethane was revealed to be an endothermic and entropically favored process, with Δ*H* = 24.6 ± 0.8 kJ mol^–1^ and Δ*S* = 83.1 ± 2.4 J K^–1^ mol^–1^. The Δ-Zn centers of (*S*)_24_-Δ_8_-**1** may thus possess slightly more conformational freedom than in the case of diastereomeric (*S*)_24_-Λ_8_-**1**, which may contain more strongly bound, but less free, Λ-Zn centers. These effects are likely to be small for each individual vertex, but the effects of the 24 pendent stereocenters cooperatively tip the thermodynamic balance from one diastereomeric form to the other.

The van ’t Hoff analysis also provided possible mechanistic insight into the solvent-controlled Δ-Zn ⇄ Λ-Zn interconversion. Based on the different hydrogen-bond acceptor abilities of acetonitrile (β = 40) and nitromethane (β = 6),^27^ we inferred that the better hydrogen-bond acceptor acetonitrile may restrict the conformational freedom of a metal vertex by accepting hydrogen bonds from the amide group, thus favoring the Λ-Zn configuration.

We then turned our attention to the exploration of the host–guest properties of cage **1**. Although porphyrin-containing metal–organic capsules have exhibited extensive fullerene-binding abilities,^28^ cage **1** was not observed to encapsulate C_60_, C_70_ and their covalent adducts (Figure S73). This observation may be attributable to the large inner cavity (2881 Å^3^) of **1** and the distance between parallel faces (19.8 Å), which preclude extensive stacking interactions between the host cage and guest fullerenes.^19^

Metal-porphyrin-containing supramolecular architectures have been used to bind pyridine-containing guests, driven by the coordination of pyridine nitrogen donors to porphyrin metal centers.^29^ Taking advantage of such coordination-mediated host–guest chemistry, bidentate 4,4′-dipyridyl naphthalenediimide guest **G1**, with a N···N distance of 15.4 Å, was investigated. The mixture of equimolar amounts of **G1** and **1** in acetonitrile led to the formation of host–guest complex **G1**⊂**1** (Figure 4, left). This complex (d.r. = 3.5:1) retained the stereochemical configuration of the parent cage **1** (d.r. = 3.8:1) (Figure S42).

**Figure 4:**
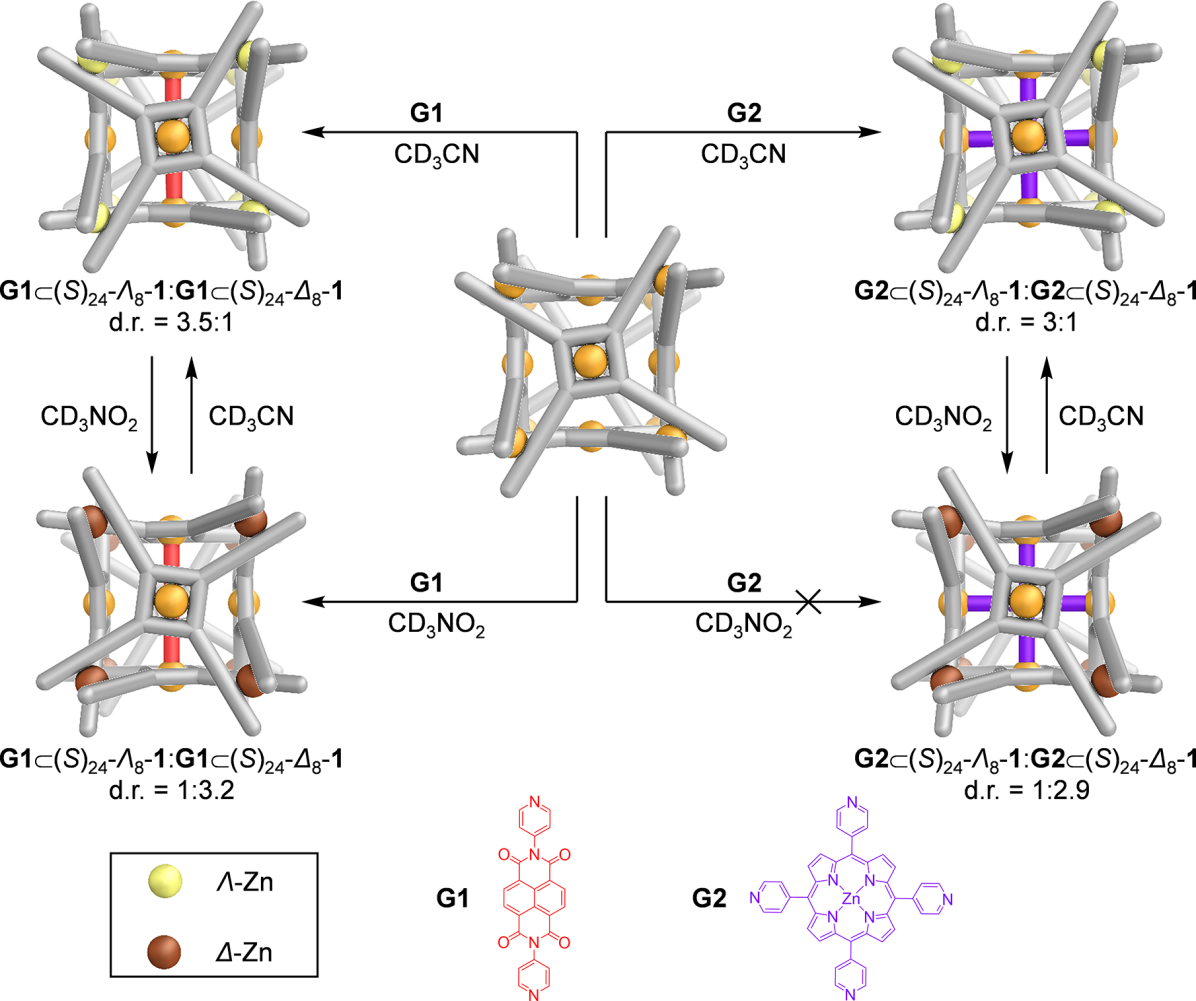
Simplified representation of the stereoretentive encapsulation of pyridine-containing guests by Zn_8_L_6_ cage **1**. Reaction conditions: encapsulation of **G1** in acetonitrile (70 °C, 16 h); encapsulation of **G1** in nitromethane (70 °C, 16 h); encapsulation of **G2** in acetonitrile (MW, 150 °C, 1.5 h).

Encapsulation of **G1** also occurred in nitromethane, furnishing **G1**⊂**1** with a lower diastereoselectivity (d.r. = 1:3.2) than was observed in the case of the empty cage (d.r. = 1:6) (Figure S51). The encapsulation of **G1** by **1** thus proceeds in a stereoretentive manner, as further confirmed by CD spectroscopy (Figure S50). The guest nonetheless influences the stereochemistry of the host metal vertices, particularly in nitromethane, leading to altered diastereomeric ratios.

Cage **1** was also observed to bind tetrapyridyl Zn-porphyrin **G2**, which measures 15.3 Å between opposing pyridyl nitrogen atoms, in acetonitrile (Figure 4, right). Host–guest complex **G2**⊂**1** was formed with a diastereomeric ratio of **G2**⊂(*S*)_24_-Λ_8_-**1**:**G2**⊂(*S*)_24_-Δ_8_-**1** = 3:1 (Figure S58). Although cage **1** was not observed to encapsulate **G2** in nitromethane, which we attribute to the insolubility of **G2** in nitromethane, dissolution of **G2**⊂**1** in this solvent gave **G2**⊂(*S*)_24_-Δ_8_-**1** with a diastereoselectivity (d.r. = 1:2.9) again favoring the opposite metal-center handedness than in acetonitrile (Figure S66).

The encapsulation of **G2** segregated the cavity of cage **1** into two symmetry-equivalent cells, enabling the binding of smaller guests that were previously not competent guests for cage **1**.^29c^**G2**⊂**1** was observed to internally bind 4,4′-bipyridine between central and exterior zinc sites (Figure S71).

The coordination-driven self-assembly of an enantiopure tetratopic ligand with either Zn^II^ or Co^II^ thus provided a straightforward strategy for the diastereoselective preparation of M_8_L_6_ cubic cages, which share the same (*S*) ligand stereochemistry but vary in the Λ- or Δ-handedness of their metal vertices. This metal-vertex handedness was switched by the solvent. Although the diastereoselectivities achieved in the present case are modest, this study may lead to the discovery of new methods by which the handedness of chiral coordination cage frameworks could be switched through a change in the environment, with no need for additional synthesis. The ability to bind pyridine-containing guests to form chiral coordination architectures such as **G1**⊂**1** and **G2**⊂**1** diastereoselectively may enable the creation of new caged metalloporphyrin catalysts capable of generating either enantiomer of a chiral product,^3,29^ which may become practically useful following the further improvements in diastereoselectivity noted above. Given that the Δ stereochemical configuration is entropically favored, future work to improve diastereoselectivity will focus on the incorporation of more flexible chiral side chains, which may enhance this entropic preference. This phenomenon of solvent-driven stereochemical switching may be general to cages with pyridyl-triazole ligands^20^ bearing chiral side chains, enabling the design of cages that are capable of selectively binding either enantiomer of a newly synthesized product, thus enabling new methods of chiral purification. Future studies will explore this solvent-driven phenomenon in cages built from ditopic, tritopic, and pentatopic ligands.

## Supporting Information Available

Experimental procedures; NMR characterizations; mass spectrometry data; CD spectra; volume calculations X-ray crystallographic data (PDF)

## Accession Codes

CCDC 2115416 and 2115489 contain the supplementary crystallographic data for this paper. These data can be obtained free of charge via www.ccdc.cam.ac.uk/data_request/cif, or by emailing data_request@ccdc.cam.ac.uk, or by contacting The Cambridge Crystallographic Data Centre, 12 Union Road, Cambridge CB2 1EZ, UK; fax: +44 1223 336033.
