# Supplementary material for: Solvent Drives Switching between Λ and Δ Metal Center Stereochemistry of M8L6 Cubic Cages
Source: J Am Chem Soc. 2022 Apr 2;144(14):6136–42. doi: 10.1021/jacs.2c00245 (PMC9098163; doi:10.1021/jacs.2c00245)
Supplement: Supplementary file 1 — ja2c00245_si_001.pdf [file ja2c00245_si_001.pdf]

Solvent Drives Switching between *Λ* and *Δ* Metal Center Stereochemistry of M_8L_6 Cubic Cages

Weichao Xue, Tanya K. Ronson, Zifei Lu, Jonathan R. Nitschke*

Yusuf Hamied Department of Chemistry, University of Cambridge,

Cambridge CB2 1EW, UK.

jrn34@cam.ac.uk

Supporting Information

Table of Contents

1 General Information S3

2 Synthesis and Characterization of Ligand **A** S5

2.1 Synthesis of Enantiopure 2-Ethynylpyridine (**S4**) S5

2.2 Synthesis of Enantiopure Ligand **A** S7

3 Self-Assembly and Characterization of Cages **1** and **2** S13

3.1 Self-Assembly of Cage **1** in MeCN S13

3.2 Self-Assembly of Cage **2** in MeCN S20

4 Investigation of Diastereocontrol for Cage **1** S24

4.1 Solvent Effect S24

4.2 Temperature Effect S34

4.3 Concentration Effect S38

5 Host-Guest Studies Using Cage **1** S40

5.1 Encapsulation of Guest **1** S40

5.2 Encapsulation of Guest **2** S51

5.3 Binding 4,4'-Bipyridine by **G2⊂1** S60

5.4 Non-Binding Guests S62

6 Volume Calculations S63

7 X-ray Crystallography S64

8 Investigation of the Monomeric ZnL_3 Complex S70

9 References S75

1 General Information

Unless otherwise specified, all reagents were purchased from commercial sources and used as received. Enantiopure 2-ethynylpyridine (**S4**) was prepared from commercially available 2-bromonicotinic acid; enantiopure ligand **A** was prepared from commercially available 5,10,15,20-tetrakis(pentafluorophenyl)porphyrin. Self-assembly reactions were performed in either deuterated solvents (CD_3CN, CD_3NO_2 and CD_3COCD_3) or distilled solvents.

NMR spectra were recorded using the following NMR spectrometers: Bruker 400 MHz Avance III HD smart probe (^1H, ^{13}C, ^{19}F, ^1H-DOSY, 2D NMR, and VT NMR experiments), Bruker Avance 500 MHz DCH cryoprobe (^1H, ^{13}C, and 2D NMR), Bruker Avance 500 MHz TCI cryoprobe (^1H NMR) or 700 MHz TXO Cryoprobe (^1H NMR). Chemical shifts of the NMR spectra are reported relative to $CDCl_3$ (^1H NMR: δ = 7.26 ppm, ^{13}C NMR: δ = 77.0 ppm), CD_3CN (^1H NMR: δ = 1.94 ppm, ^{13}C NMR: δ = 118.3 ppm), CD_3NO_2 (^1H NMR: δ = 4.33 ppm, ^{13}C NMR: δ = 62.8 ppm), CD_3COCD_3 (^1H NMR: δ = 2.05 ppm, ^{13}C NMR: δ = 29.9 ppm), and CD_3OD (^1H NMR: δ = 3.31 ppm, ^{13}C NMR: δ = 49.0 ppm). Data for ^1H NMR spectra were reported as follows: chemical shift (ppm), peak shape (s = singlet, d = doublet, t = triplet, m = multiplet), coupling constant (Hz), and integration. Data for ^{13}C NMR and ^{19}F NMR are reported in terms of chemical shift (ppm).

UV-vis measurements were employed to fine-tune the solution concentration for subsequent CD measurements, and were performed on a Varian Cary 400 scan UV-vis spectrophotometer with a 1 mm path-length cuvette at 298 K. Circular Dichroism was performed on an Applied-Photophysics Chirascan CD spectrometer using a 1 mm path-length cuvette. Experiments were recorded at 298 K, maintained with a Peltier temperature control. Measurements were background subtracted from blank solvent in an identical cuvette. The sample concentrations were adjusted to maintain a HV below 800 V.

Low resolution electrospray ionization mass spectrometry was undertaken on a Micromass Quattro LC mass spectrometer (cone voltage 20-50 eV; desolvation temp. 40 °C; ionization temp. 40 °C) infused from a Harvard syringe pump at a rate of 10 μL/min.

A microwave reactor from Discover SP-D 80-CEM Corporation was used for the encapsulation of tetrapyridyl Zn-porphyrin by Zn_8L_6 cage.

2 Synthesis and Characterization of Ligand A

2.1 Synthesis of Enantiopure 2-Ethynylpyridine (S4)

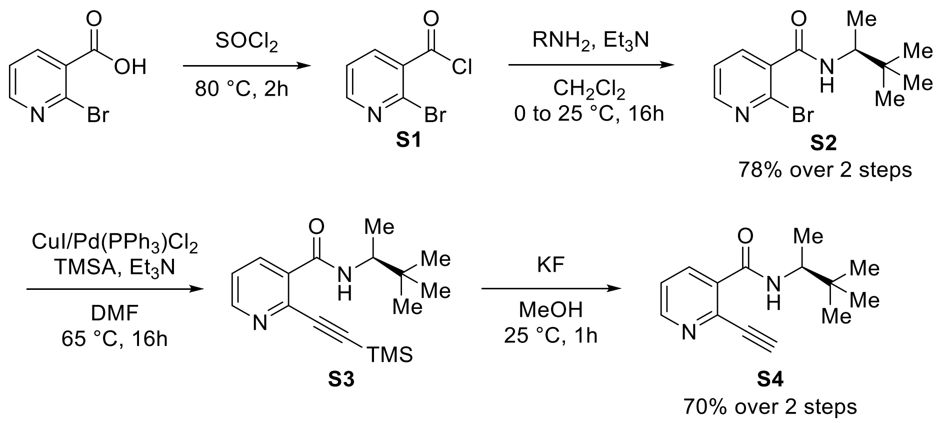

A suspension of 2-bromonicotinic acid (2.02 g, 10.0 mmol, 1.0 equiv) in $SOCl_2$ (15 mL) was heated to reflux at 80 °C for 2 hours. After cooling down to room temperature, excess $SOCl_2$ was evaporated under reduced pressure, and Et_3N (2.02 g, 20.0 mmol, 2.0 equiv) in CH_2Cl_2 (20 mL) was subsequently added. The solution was then cooled to 0 °C, and (*S*)-3,3-dimethyl-2-butylamine (1.01 g, 10.0 mmol, 1.0 equiv) was added over 30 minutes. The reaction mixture was warmed to room temperature and stirred for 16 hours. The resulting suspension was washed with brine (20 mL) and saturated aqueous $NaHCO_3$ solution (20 mL). The combined aqueous phases were extracted with CH_2Cl_2 (2 × 20 mL). The combined organic phases were dried over anhydrous Na_2SO_4, filtered, and the solvents were evaporated under reduced pressure. The crude compound **S2** was obtained as a pale yellow solid (2.22 g, 78% yield over 2 steps) and used without purification.

A suspension of compound **S2** (1.15 g, 5.0 mmol, 1.0 equiv), trimethylsilylacetylene (TMSA, 737 mg, 7.5 mmol, 1.5 equiv), Et_3N (506 mg, 5.0 mmol, 1.0 equiv), CuI (47.6 mg, 0.25 mmol, 0.05 equiv) and $Pd(PPh_3)_2Cl_2$ (175 mg, 0.25 mmol, 0.05 equiv) in dimethylformamide (DMF, 10 mL) was degassed under a stream of nitrogen for 20 mins. The reaction mixture was heated at 65 °C for 16 hours under nitrogen. After cooling down to room temperature, excess TMSA and DMF were evaporated under reduced pressure. KF (1.45 g, 25.0 mmol, 5.0 equiv) and MeOH (10 mL) were

subsequently added, and the reaction mixture was stirred for 1 hour at room temperature. Brine (50 mL) was then added, and the suspension was extracted with CH_2Cl_2 (3 × 50 mL). The combined organic phases were dried over anhydrous Na_2SO_4, filtered, and the solvents evaporated under reduced pressure. Purification by flash column chromatography on silica gel using hexane/EtOAc = 2/3 as eluent afforded **S4** as a brown solid (807 mg, 70% yield over 2 steps).

R_f = 0.40 (hexane/EtOAc = 2/3). **^{1}H NMR** (400 MHz, $CDCl_3$): δ 0.99 (s, 9H), 1.19 (d, *J* = 4.5 Hz, 3H), 3.57 (s, 1H), 4.09–4.19 (m, 1H), 7.22 (d, *J* = 8.4 Hz, 1H), 7.41 (dd, *J* = 7.9, 4.7 Hz, 1H), 7.41 (dd, *J* = 7.9, 1.8 Hz, 1H), 7.41 (dd, *J* = 4.7, 1.8 Hz, 1H) ppm. **^{13}C NMR** (100 MHz, $CDCl_3$): δ 15.9, 26.4, 34.4, 54.3, 82.2, 82.9, 123.9, 133.2, 137.5, 138.1, 151.4, 163.6 ppm.

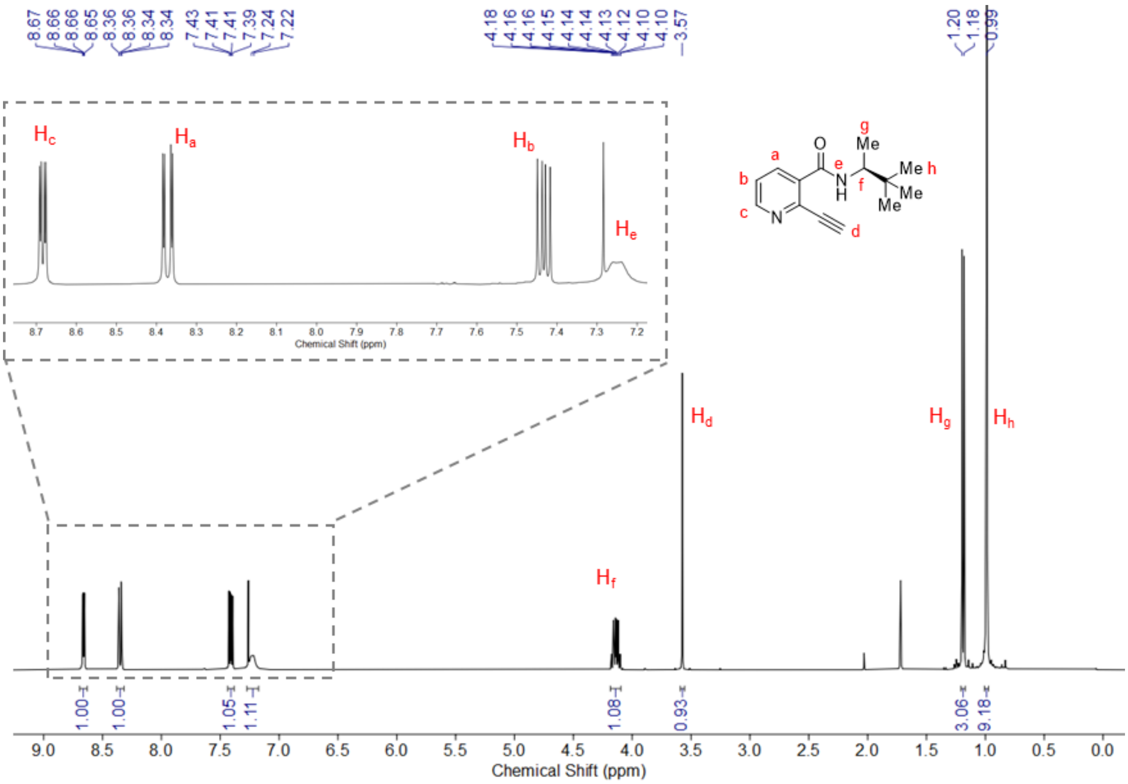

Figure S1. ^{1}H NMR spectrum of compound **S4** (400 MHz, $CDCl_3$, 25 °C).

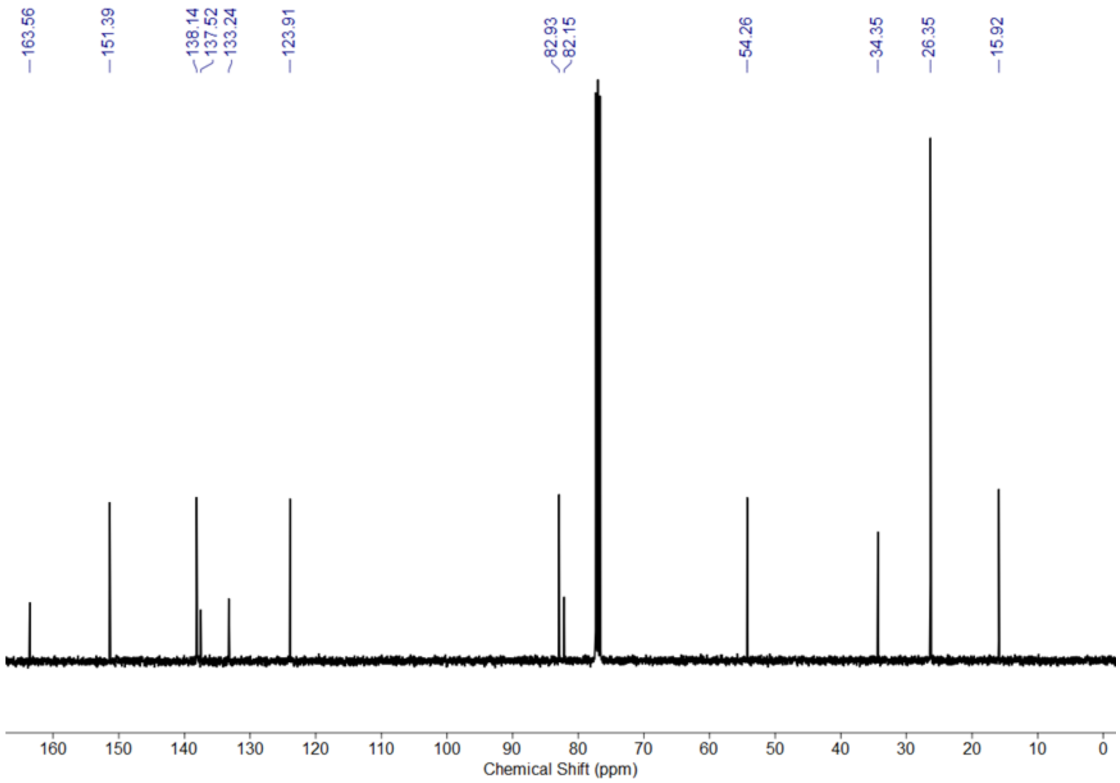

Figure S2. ^{13}C NMR spectrum of compound **S4** (400 MHz, $CDCl_3$, 25 °C).

2.2 Synthesis of Enantiopure Ligand A

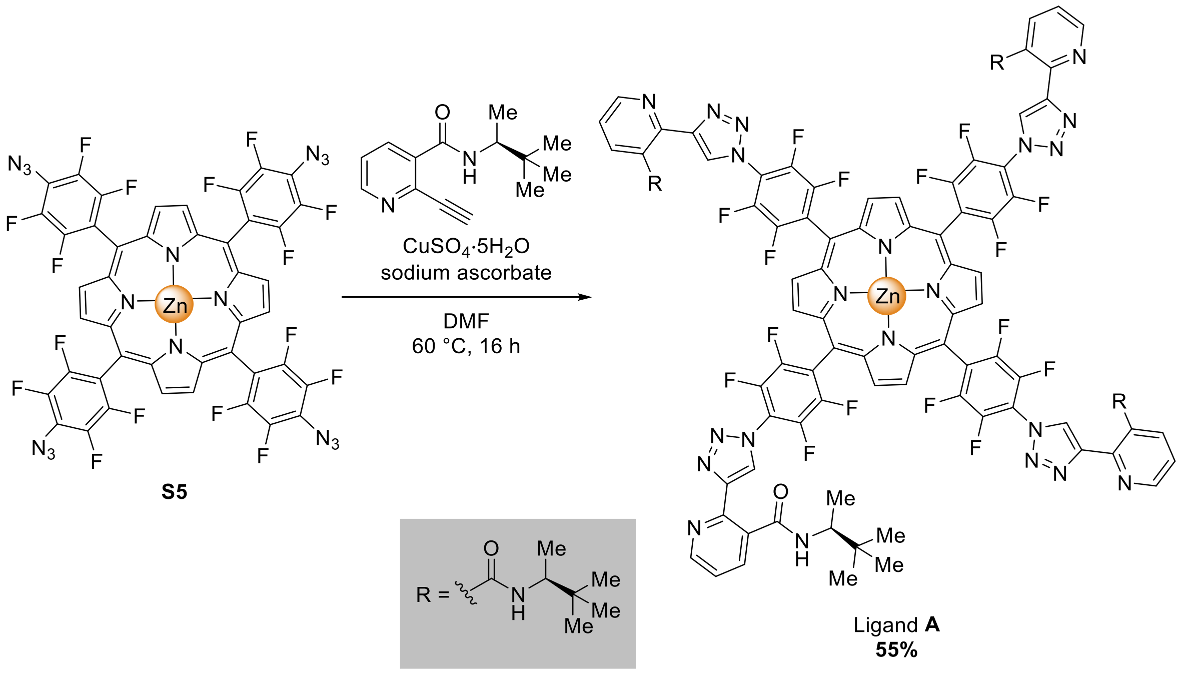

According to the reported metalation and azidation reactions, zinc-5,10,15,20-tetrakis (2,3,5,6-tetrafluoro-4-azidophenyl)porphyrin (**S5**) was prepared from commercially available 5,10,15,20-tetrakis(pentafluorophenyl) porphyrin.[1,2] To a solution of **S5** (170 mg, 0.15 mmol, 1.0 equiv) in DMF (10 mL) was added $CuSO_4 \cdot 5H_2O$ (22.5 mg, 0.09 mmol, 0.6 equiv), sodium ascorbate (35.7 mg, 0.18 mmol, 1.2 equiv) and **S4** (172.3 mg, 0.75 mmol, 5.0 equiv). The reaction mixture was heated at 65 °C for 16 hours under nitrogen. Brine (100 mL) was then added, and the suspension was extracted with $CHCl_3$ (3 × 100 mL). The combined organic phases were dried over anhydrous Na_2SO_4, filtered, and the solvents evaporated under reduced pressure. Purification by flash column chromatography on silica gel using MeOH/EtOAc = 1/30 as eluent afforded ligand **A** as a purple solid (169 mg, 55%).

No Cotton effects were observed for ligand **A** in both MeCN and $MeNO_2$ (Figure S10).

R_f = 0.25 (MeOH/EtOAc = 1/30). **^{1}H NMR** (400 MHz, CD_3OD): δ 1.01 (s, 36H), 1.24 (d, *J* = 6.4 Hz, 12H), 4.06–4.19 (m, 4H), 7.52–7.63 (m, 4H), 7.97 (d, *J* = 7.5 Hz, 4H), 8.79 (d, *J* = 4.1 Hz, 4H), 9.12 (s, 4H), 9.28 (s, 8H) ppm. **^{13}C NMR** (100 MHz, CD_3OD): δ 15.0, 26.9, 35.7, 55.3, 105.2, 118.6–119.3 (m, C_6F_4), 124.6, 124.8–125.4 (m, C_6F_4), 128.5, 133.4, 134.1, 137.9, 141.9–142.5 (m, C_6F_4), 144.4–145.1 (m, C_6F_4), 146.7–147.2 (m, C_6F_4), 146.8, 148.2, 149.0–149.7 (m, C_6F_4), 151.2, 151.5, 170.5 ppm. **^{19}F NMR** (470 MHz, CD_3OD): δ -151.46 (dd, *J* = 22.2, 8.9 Hz, 8F), -140.12 (dd, *J* = 22.2, 8.9 Hz, 8F) ppm. **UV/Vis** (MeCN): λ_{max} 417 nm (Soret band), λ_{max} 550 nm (Q band).

Note: Activated H_f (NH) is not observed in CD_3OD due to H/D exchange.

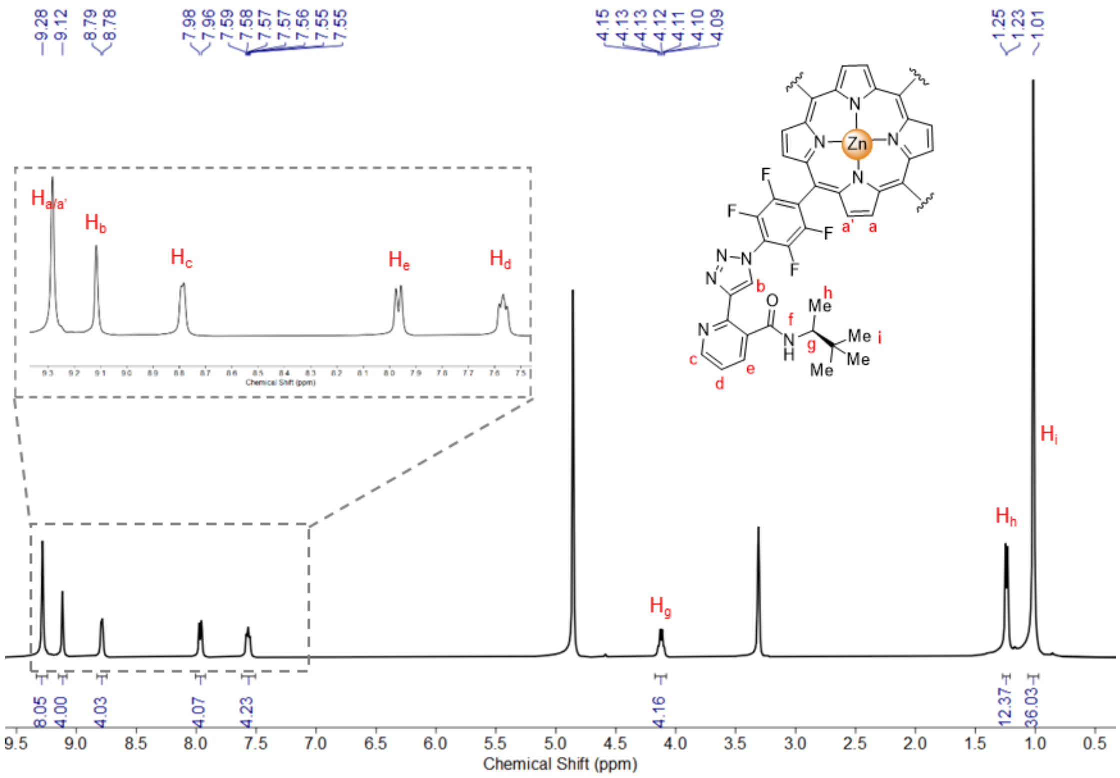

Figure S3. ^{1}H NMR spectrum of ligand **A** (400 MHz, CD_3OD, 25 °C).

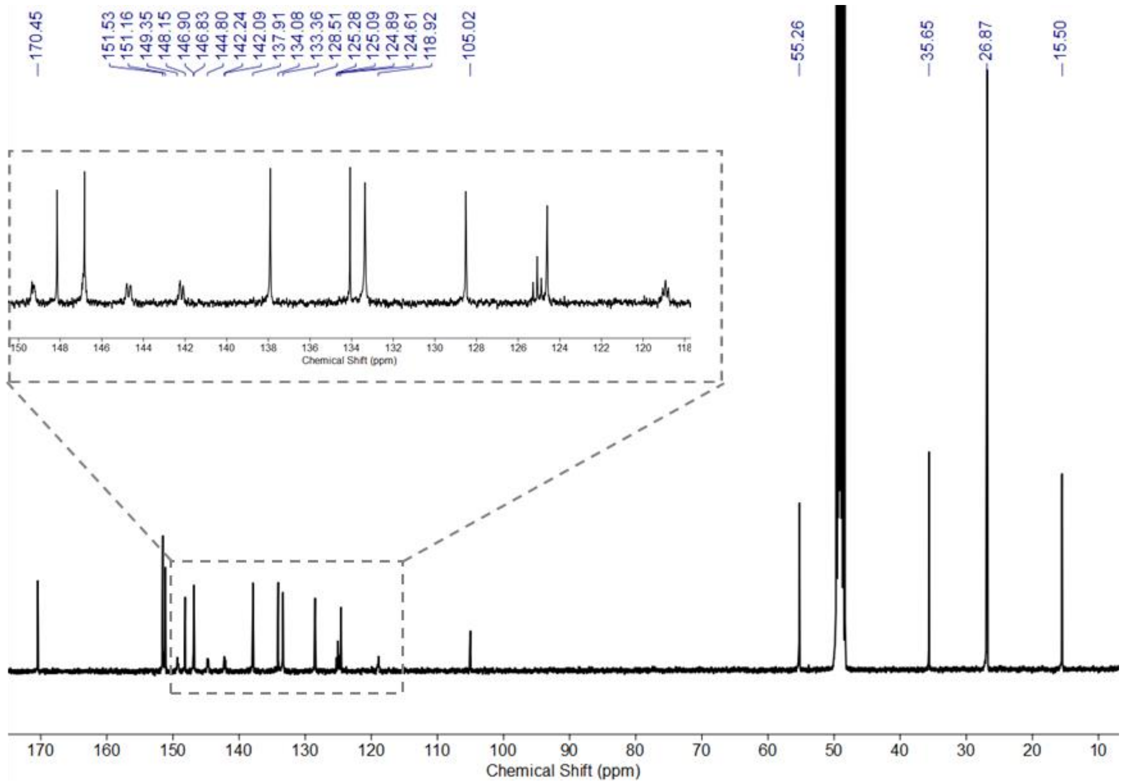

Figure S4. ^{13}C NMR spectrum of ligand **A** (100 MHz, CD_3OD, 25 °C).

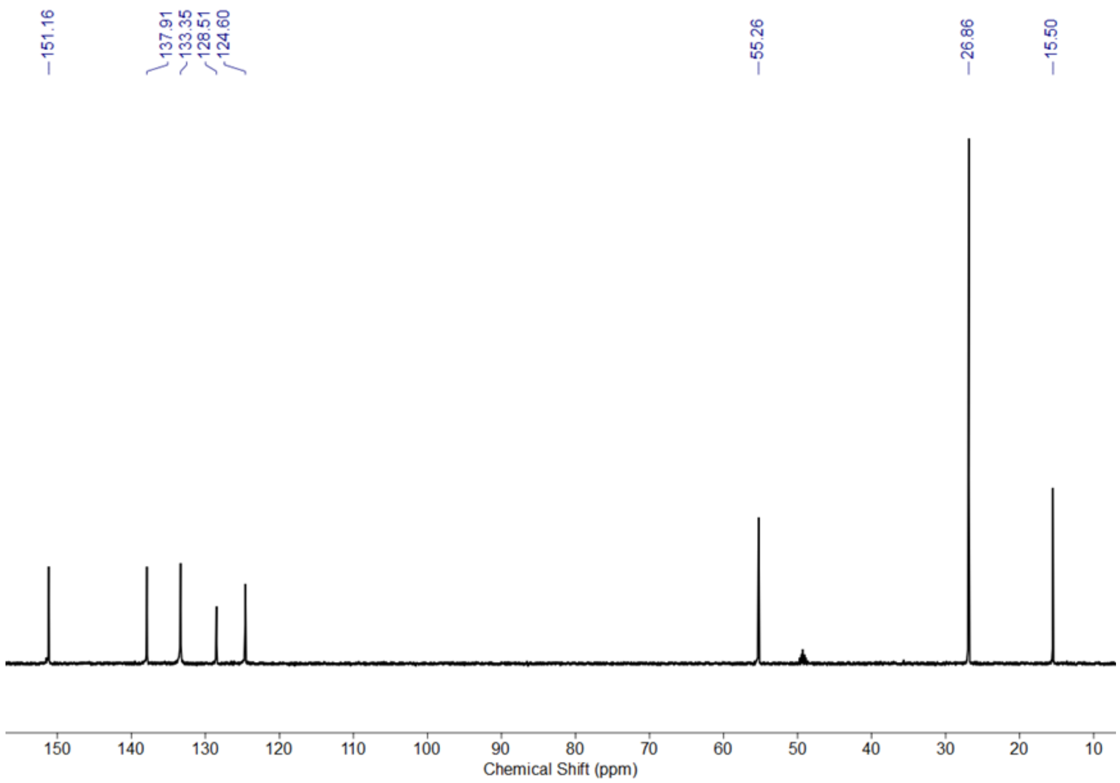

Figure S5. ^{13}C DEPT-135 NMR spectrum of ligand **A** (100 MHz, CD_3OD, 25 °C).

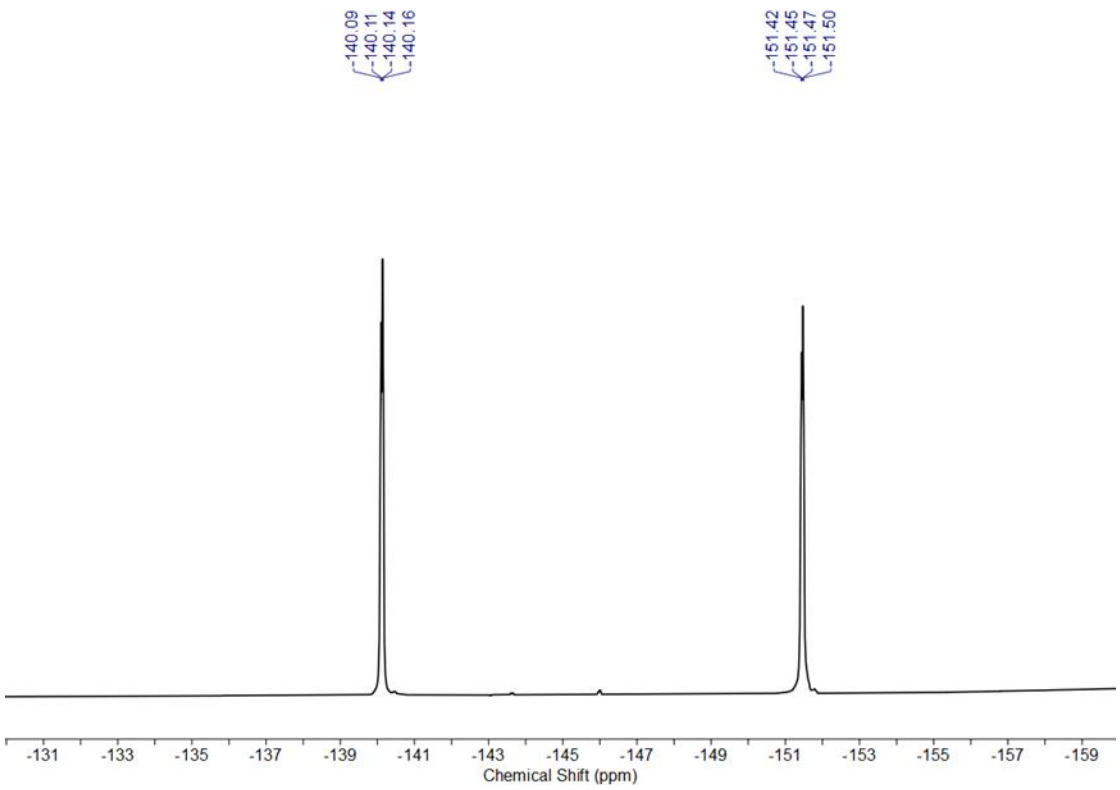

Figure S6. ^{19}F NMR spectrum of ligand **A** (470 MHz, CD_3OD, 25 °C).

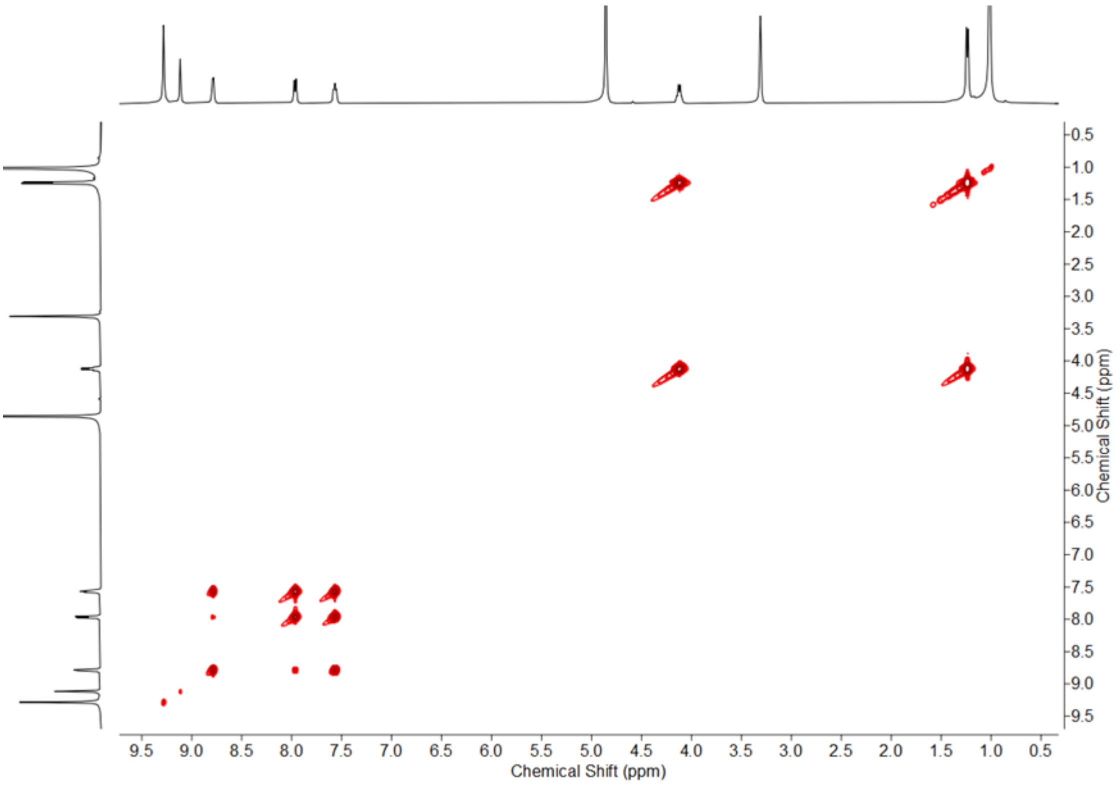

Figure S7. ^{1}H-^{1}H COSY NMR spectrum of ligand **A** (400 MHz, CD_3OD, 25 °C).

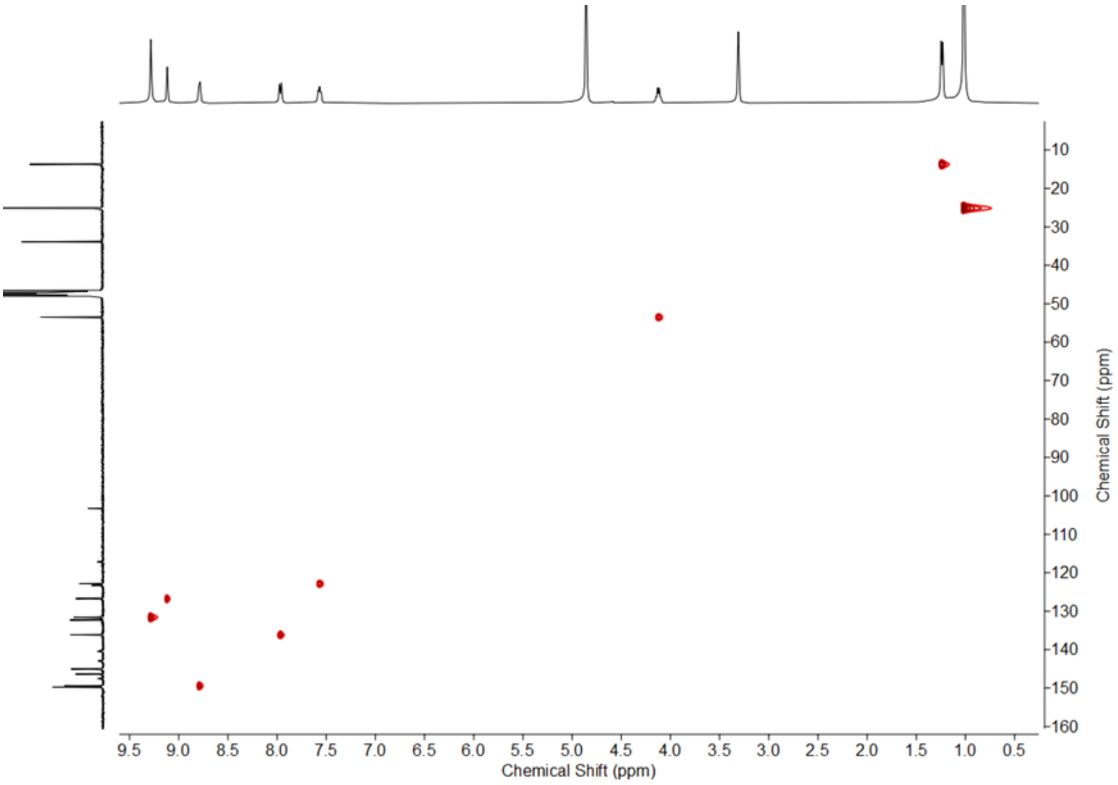

Figure S8. ^{1}H-^{13}C HSQC NMR spectrum of ligand **A** (400 MHz, CD_3OD, 25 °C).

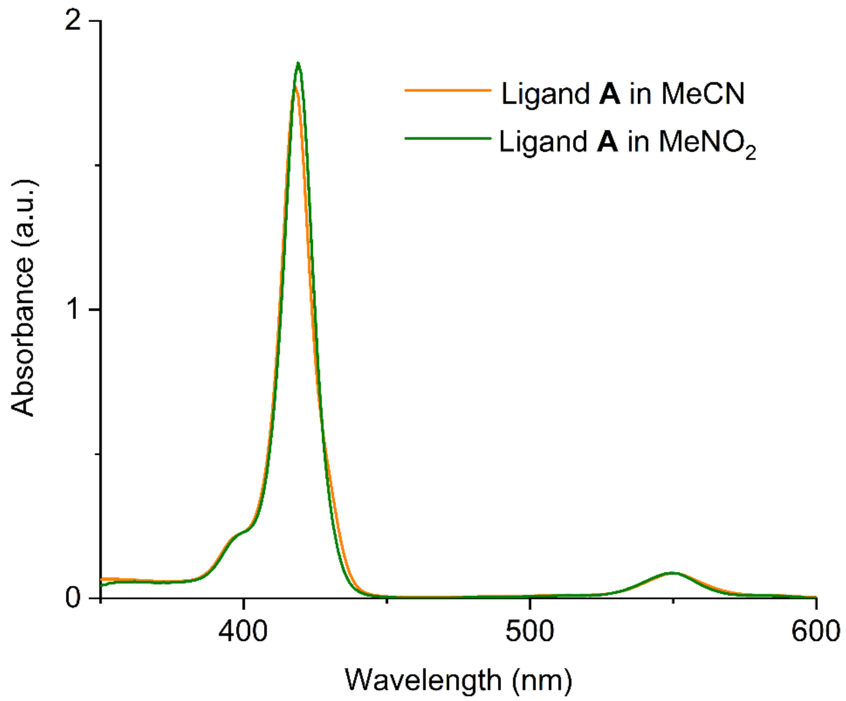

Figure S9. UV/Vis spectra of ligand **A** in MeCN and $MeNO_2$.

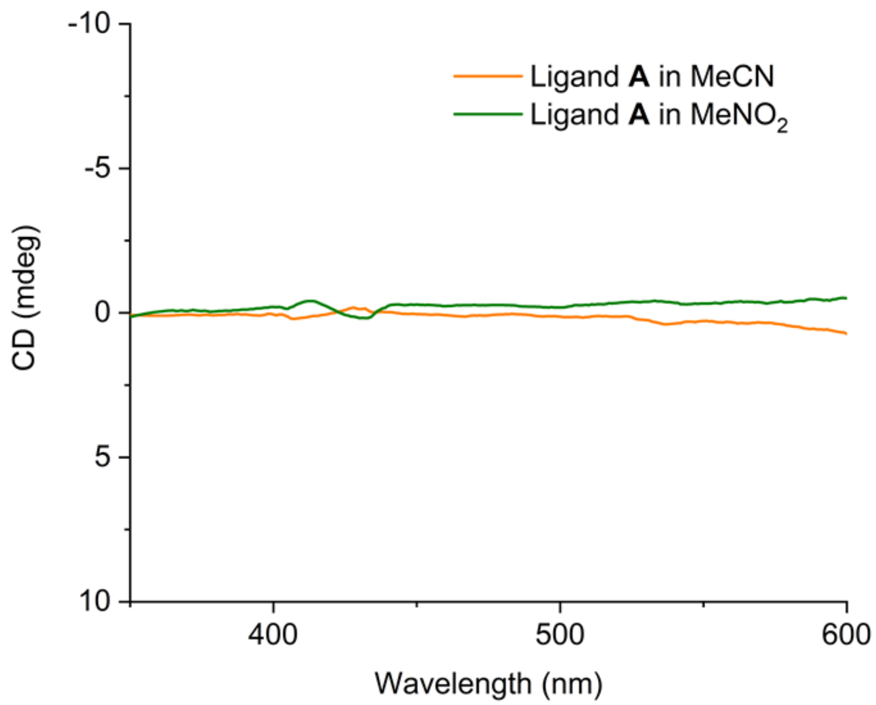

Figure S10. CD spectra of ligand **A** in MeCN and $MeNO_2$.

3 Self-Assembly and Characterization of Cages 1 and 2

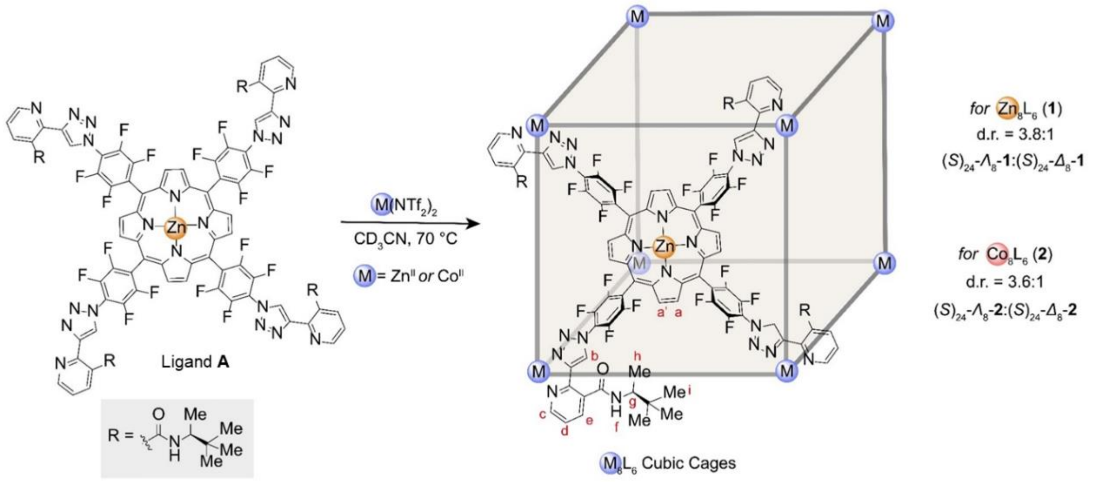

3.1 Self-Assembly of Cage 1 in MeCN

Ligand **A** (41.0 mg, 20.0 μmol, 1.0 equiv) and $Zn(NTf_2)_2$ (16.8 mg, 26.8 μmol, 1.34 equiv) were combined in MeCN (5 mL) in a 15 mL tube. The reaction mixture was stirred at 70 °C for 2 hours. The solvent was reduced to 2 mL, and Et_2O (30 mL) was then added. The precipitate was collected by centrifugation and washed with excess Et_2O, affording cage **1** as a purple solid (55.5 mg, 96%).

Cage **1** consists of a pair of diastereomers, with each having either eight Δ or eight Λ zinc vertices. According to the ^{1}H NMR spectrum, the accurate diastereomeric ratio (d.r.) was determined to be 3.8:1. Based on the assumption that the major diastereomer has crystallized from MeCN, X-ray crystallographic analysis allowed identification of the absolute configuration of the major diastereomer. The major diastereomer was identified to be (S)$_{24}$-Λ_8-**1**, while the minor diastereomer is (S)$_{24}$-Δ_8-**1**.

^{1}H NMR (500 MHz, CD_3CN): δ 1.06 (s, 171H, (S)$_{24}$-Λ_8-**1**), 1.09 (s, 45H, (S)$_{24}$-Δ_8-**1**),1.26 (d, J = 6.7 Hz, 15H, (S)$_{24}$-Δ_8-**1**), 1.36 (d, J = 6.7 Hz, 57 H, (S)$_{24}$-Λ_8-**1**), 4.20–4.30 (m, 24H, (S)$_{24}$-Λ_8-**1**+(S)$_{24}$-Δ_8-**1**), 7.25–7.38 (m, 24H, (S)$_{24}$-Λ_8-**1**+(S)$_{24}$-Δ_8-**1**), 7.86–7.97 (m, 24H, (S)$_{24}$-Λ_8-**1**+(S)$_{24}$-Δ_8-**1**), 8.40–8.55 (m, 48H, (S)$_{24}$-Λ_8-**1**+(S)$_{24}$-Δ_8-**1**), 9.02–9.10 (m, 24H, (S)$_{24}$-Λ_8-**1**+(S)$_{24}$-Δ_8-**1**), 9.17–9.25 (m, 24H, (S)$_{24}$-Λ_8-**1**+(S)$_{24}$-Δ_8-**1**),

9.26 (d, J = 2.3 Hz, 5H, (S)$_{24}$-Δ_8-**1**), 9.36 (d, J = 2.3 Hz, 19 H, (S)$_{24}$-Λ_8-**1**) ppm. **UV/Vis** (MeCN): λ_{max} 413 nm (Soret band), λ_{max} 548 nm (Q band). **ESI-MS**: m/z = 801.4 [**1**-16(NTf_2)]$^{16+}$, 873.8 [**1**-15(NTf_2)]$^{15+}$, 956.3 [**1**-14(NTf_2)]$^{14+}$, 1051.4 [**1**-13(NTf_2)]$^{13+}$, 1162.4 [**1**-12(NTf_2)]$^{12+}$, 1293.4 [**1**-11(NTf_2)]$^{11+}$, 1450.8 [**1**-10(NTf_2)]$^{10+}$,1643.4 [**1**-9(NTf_2)]$^{9+}$.

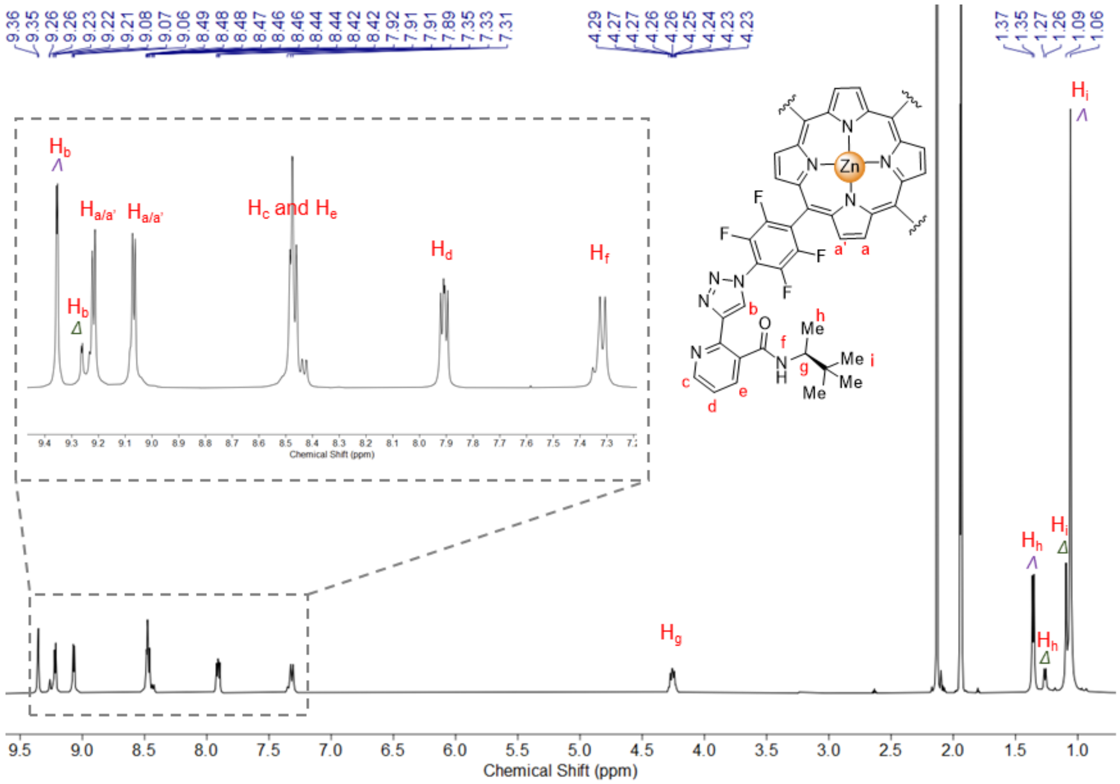

Figure S11. ^{1}H NMR spectrum of cage **1** (500 MHz, CD_3CN, 25 °C).

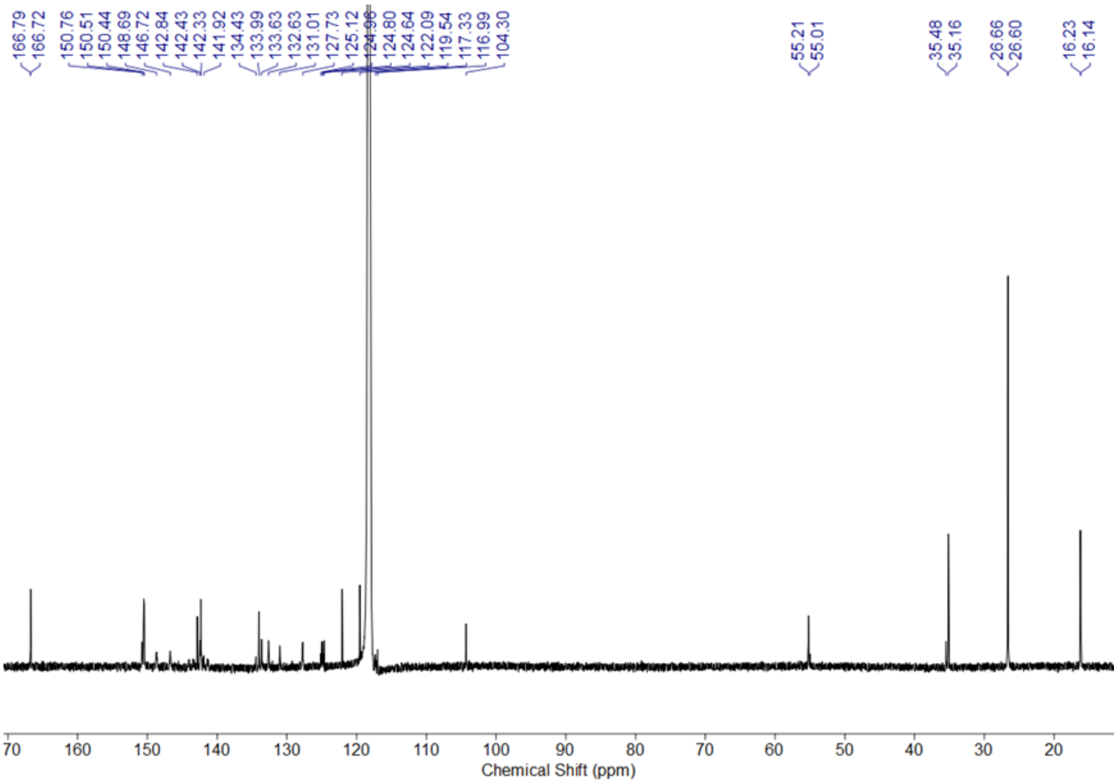

Figure S12. ^{13}C NMR spectrum of cage **1** (126 MHz, CD_3CN, 25 °C).

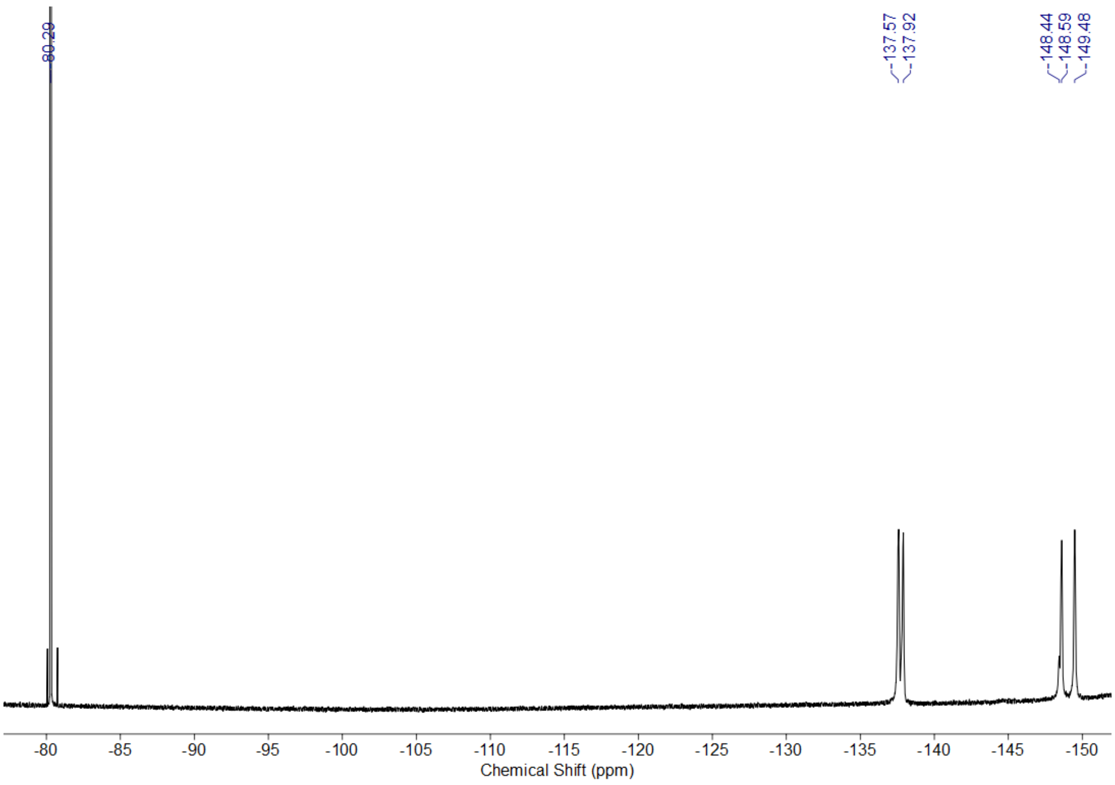

Figure S13. ^{19}F NMR spectrum of cage **1** (470 MHz, CD_3CN, 25 °C).

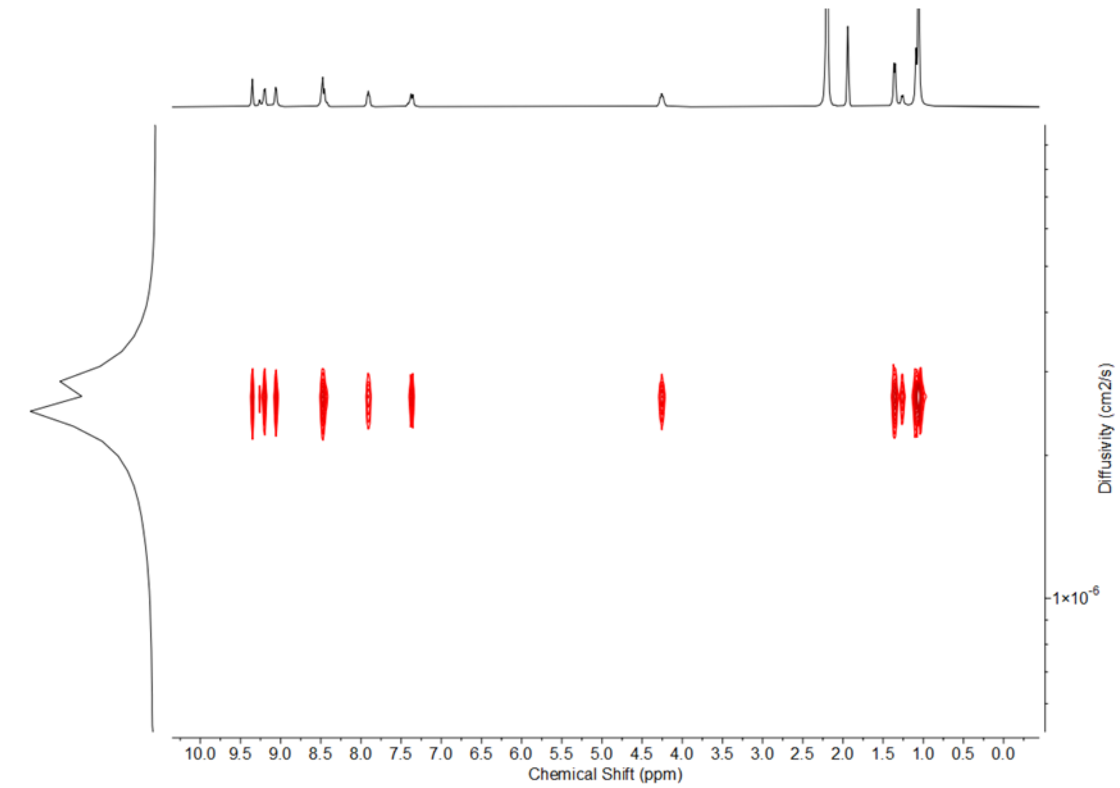

Figure S14. ^{1}H DOSY spectrum of cage **1** (400 MHz, CD_3CN, 25 °C). The diffusion coefficient for both diastereomers in CD_3CN was measured to be 2.65×10^{-6} cm^2/s.

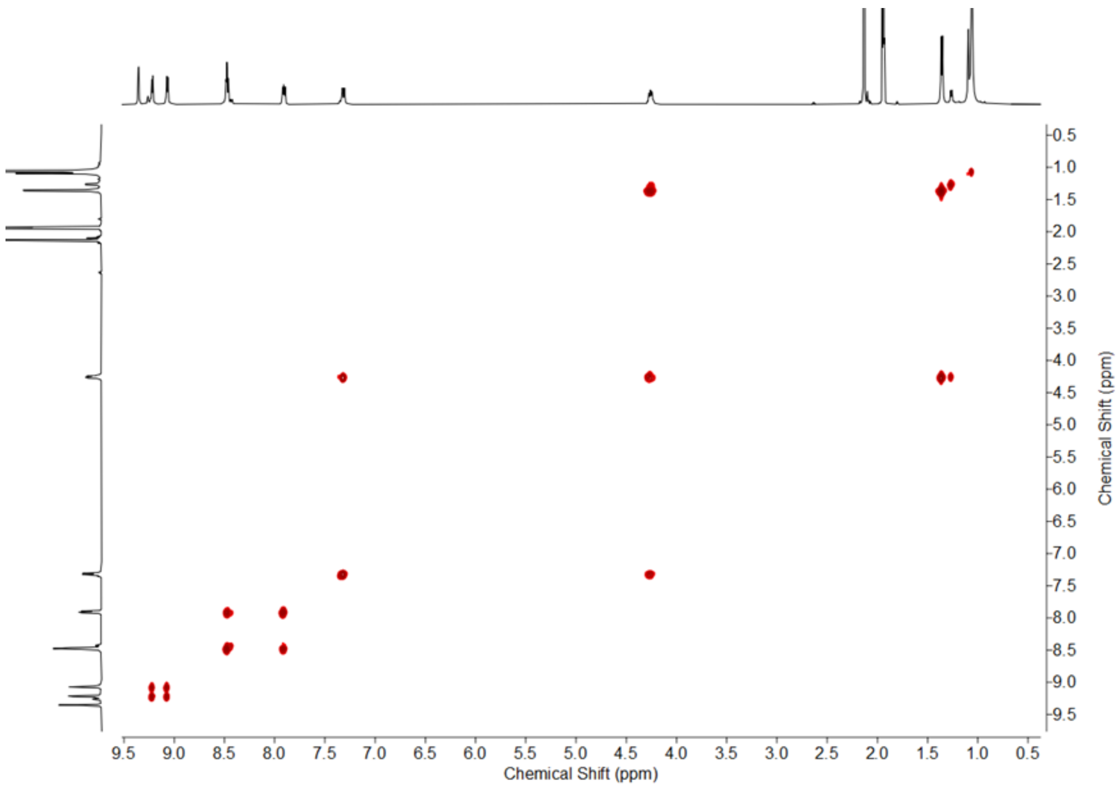

Figure S15. ^{1}H-^{1}H COSY NMR spectrum of cage **1** (500 MHz, CD_3CN, 25 °C).

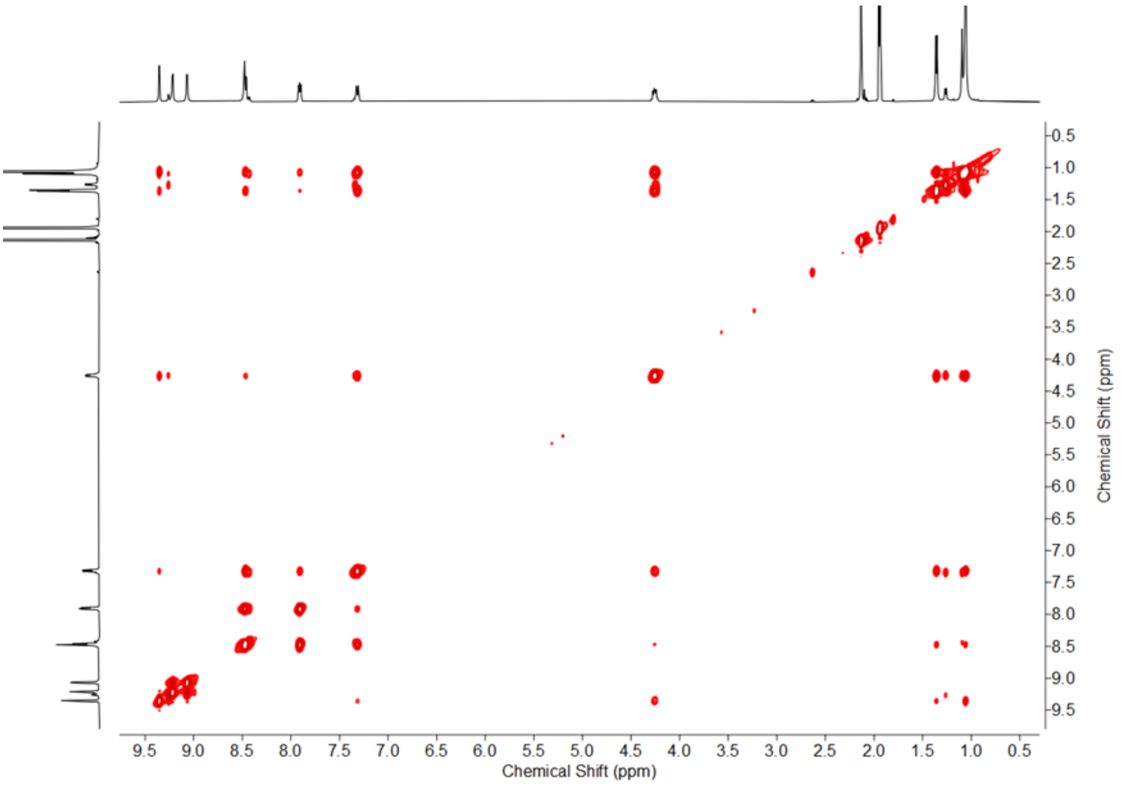

Figure S16. ^{1}H-^{1}H NOESY NMR spectrum of cage **1** (500 MHz, CD_3CN, 25 °C).

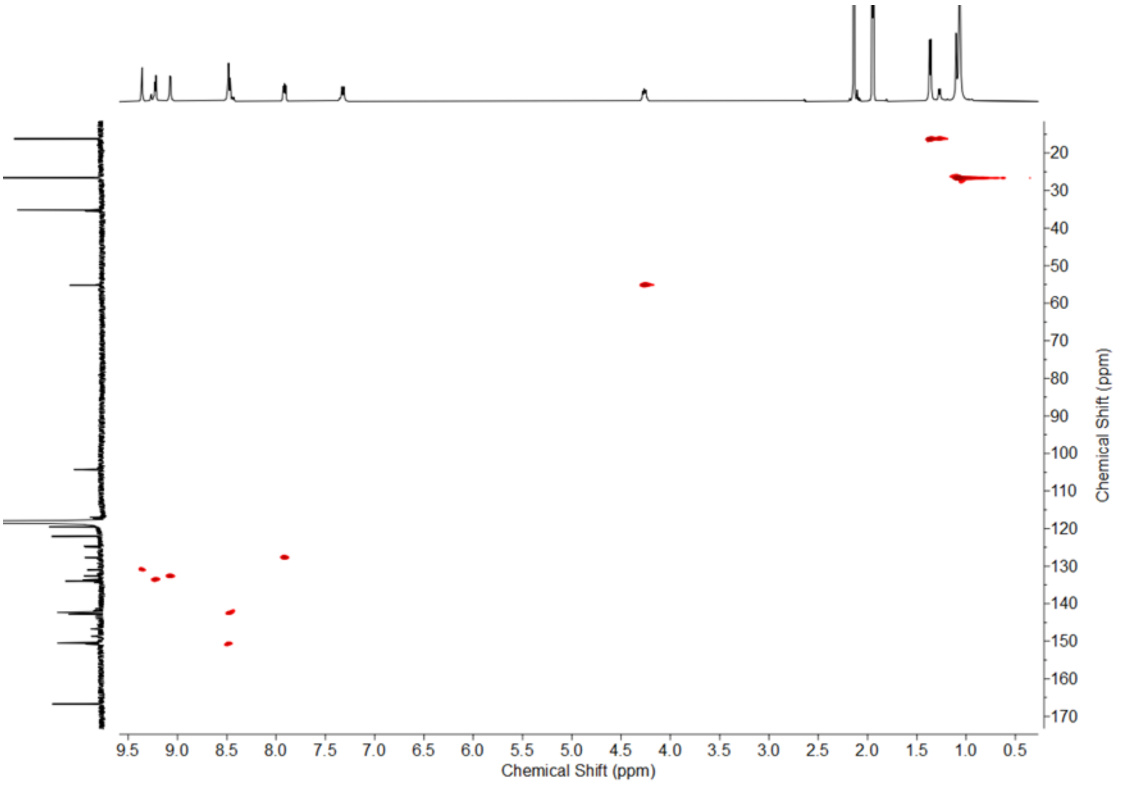

Figure S17. ^{1}H-^{13}C HSQC NMR spectrum of cage **1** (500 MHz, CD_3CN, 25 °C).

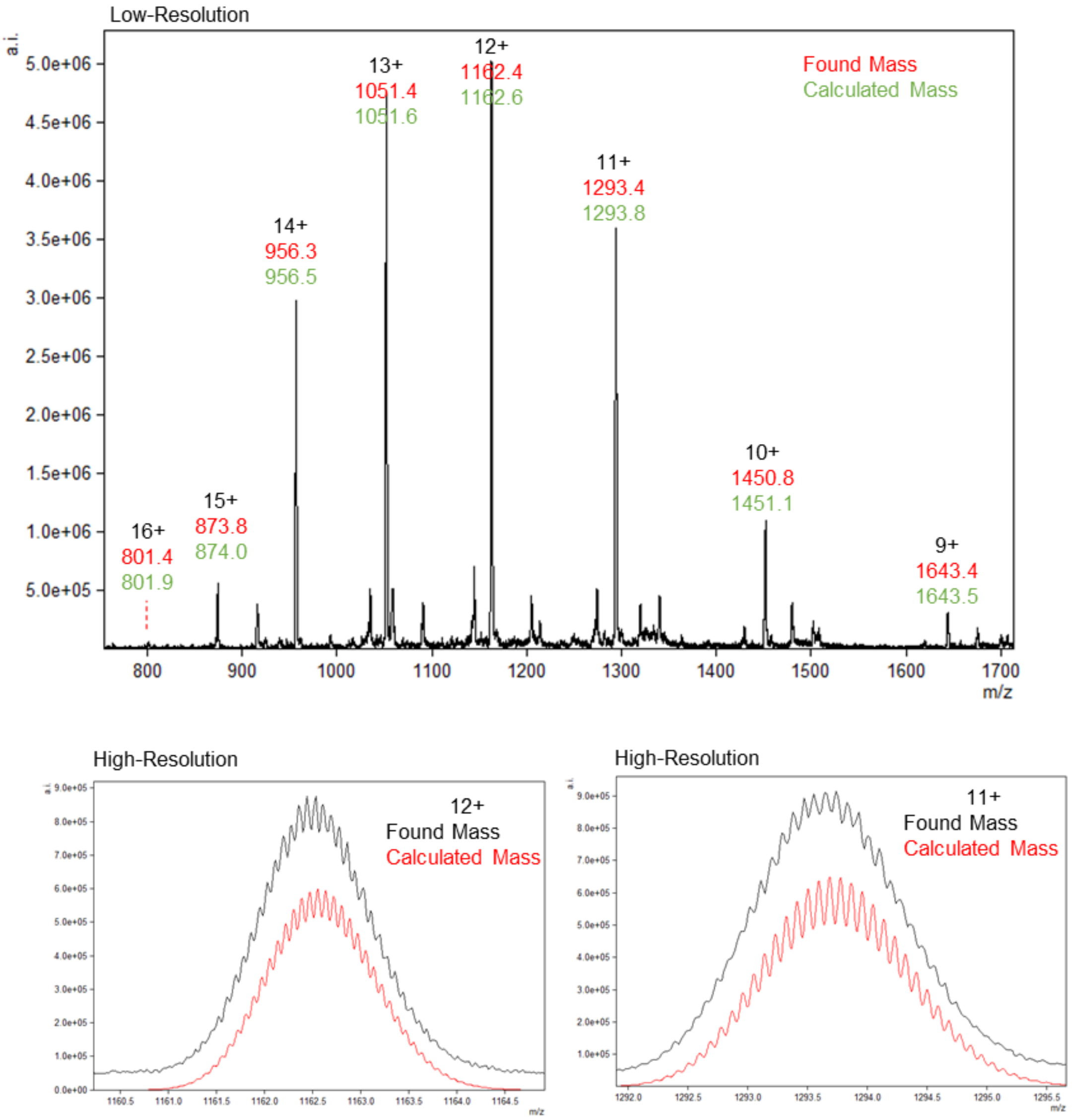

Figure S18. Low-resolution and high-resolution ESI-MS spectra of cage **1** in MeCN.

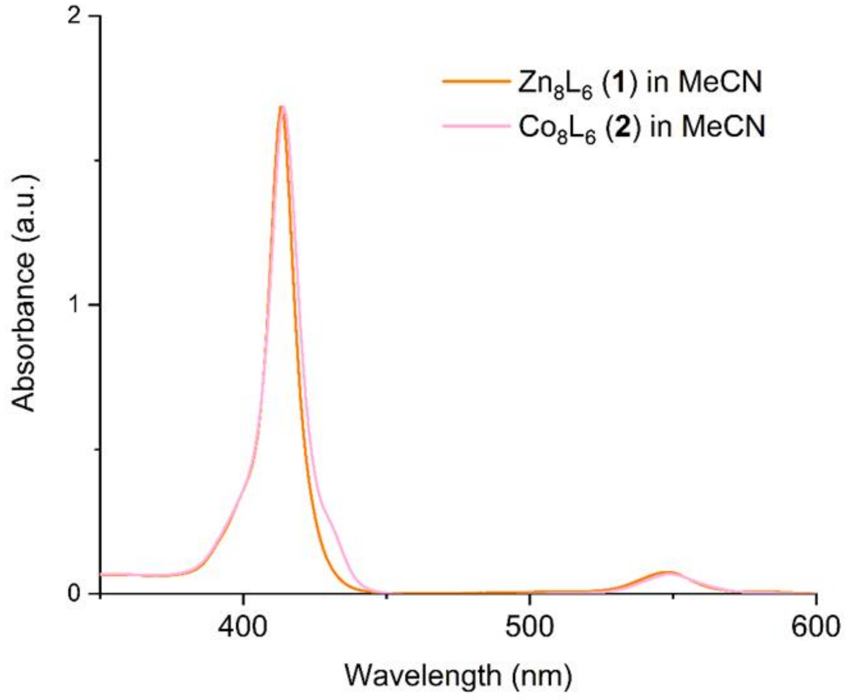

Figure S19. UV/Vis spectra of cages **1** and **2** in MeCN.

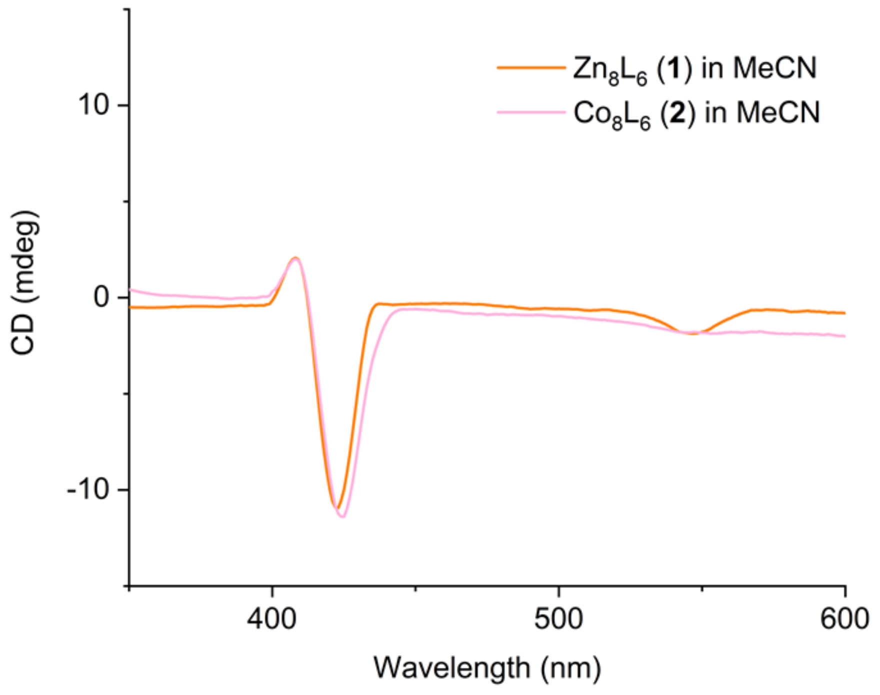

Figure S20. CD spectra of cages **1** and **2** in MeCN.

3.2 Self-Assembly of Cage 2 in MeCN

Ligand **A** (20.5 mg, 10.0 μmol, 1.0 equiv) and $Co(NTf_2)_2$ (8.30 mg, 13.4 μmol, 1.34 equiv) were combined in MeCN (2.5 mL) in a 15 mL tube. The reaction mixture was stirred at 70 °C for 2 hours. The solvent was reduced to 1 mL, and Et_2O (10 mL) was then added. The precipitate was collected by centrifugation and washed with excess diethyl ether, affording cage **2** as a purple solid (27.4 mg, 95%).

Cage **2** consists of a pair of diastereomers, with each having either eight *Δ* or eight *Λ* zinc vertices. According to the ^{1}H NMR spectrum, the accurate diastereomeric ratio (d.r.) was determined to be 3.6:1. Based on the assumption that the major diastereomer has crystallized in MeCN, X-ray crystallographic analysis allowed identification of the absolute configuration of the major diastereomer. The major diastereomer was identified to be $(S)_{24}$-$Λ_8$-**1**, while the minor diastereomer is $(S)_{24}$-$Δ_8$-**1**.

^{1}H NMR (500 MHz, CD_3CN): δ 3.63 [$(S)_{24}$-$Δ_8$-**1**], 4.05 [$(S)_{24}$-$Λ_8$-**1**], 4.16–4.50 [$(S)_{24}$-$Λ_8$-**1**+$(S)_{24}$-$Δ_8$-**1**], 5.10–5.50 [$(S)_{24}$-$Λ_8$-**1**+$(S)_{24}$-$Δ_8$-**1**], 5.72–6.30 [$(S)_{24}$-$Λ_8$-**1**+$(S)_{24}$-$Δ_8$-**1**], 8.59 [$(S)_{24}$-$Λ_8$-**1**], 8.84 [$(S)_{24}$-$Δ_8$-**1**], 12.56 [$(S)_{24}$-$Λ_8$-**1**], 13.17 [$(S)_{24}$-$Δ_8$-**1**], 14.56 [$(S)_{24}$-$Λ_8$-**1**], 14.92 [$(S)_{24}$-$Δ_8$-**1**], 38.84 [$(S)_{24}$-$Λ_8$-**1**+$(S)_{24}$-$Δ_8$-**1**], 43.67 [$(S)_{24}$-$Λ_8$-**1**+$(S)_{24}$-$Δ_8$-**1**], 88.02 [$(S)_{24}$-$Λ_8$-**1**+$(S)_{24}$-$Δ_8$-**1**] ppm. **UV/Vis** (MeCN): $λ_{max}$ 414 nm (Soret band), $λ_{max}$ 549 nm (Q band). **ESI-MS**: m/z = 798.6 [**2**-16(NTf_2)]$^{16+}$, 870.5 [**2**-15(NTf_2)]$^{15+}$, 952.7 [**2**-14(NTf_2)]$^{14+}$, 1047.5 [**2**-13(NTf_2)]$^{13+}$, 1158.1 [**2**-12(NTf_2)]$^{12+}$, 1288.8 [**2**-11(NTf_2)]$^{11+}$, 1445.9 [**2**-10(NTf_2)]$^{10+}$,1637.5 [**2**-9(NTf_2)]$^{9+}$.

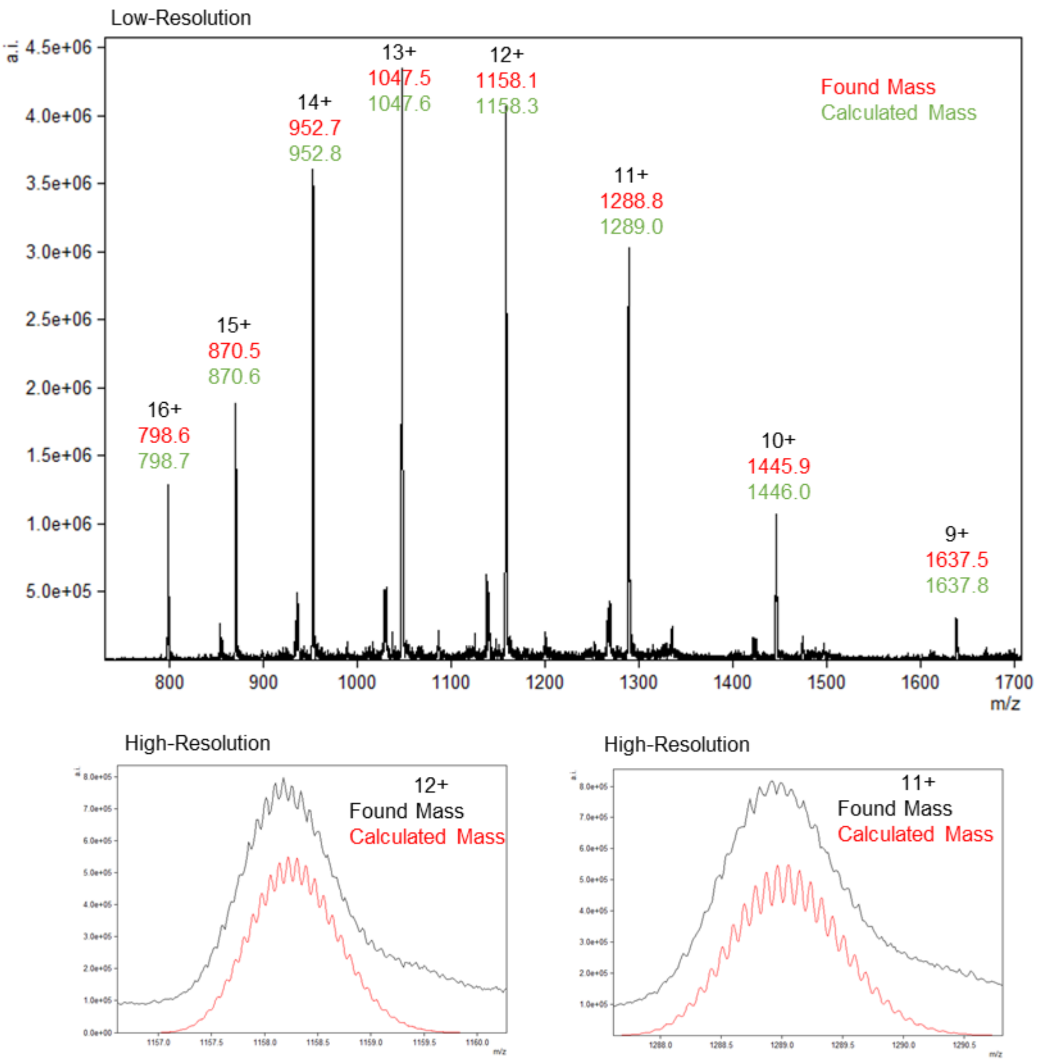

Figure S21. Low-resolution and high-resolution ESI-MS spectra of cage **2** in MeCN.

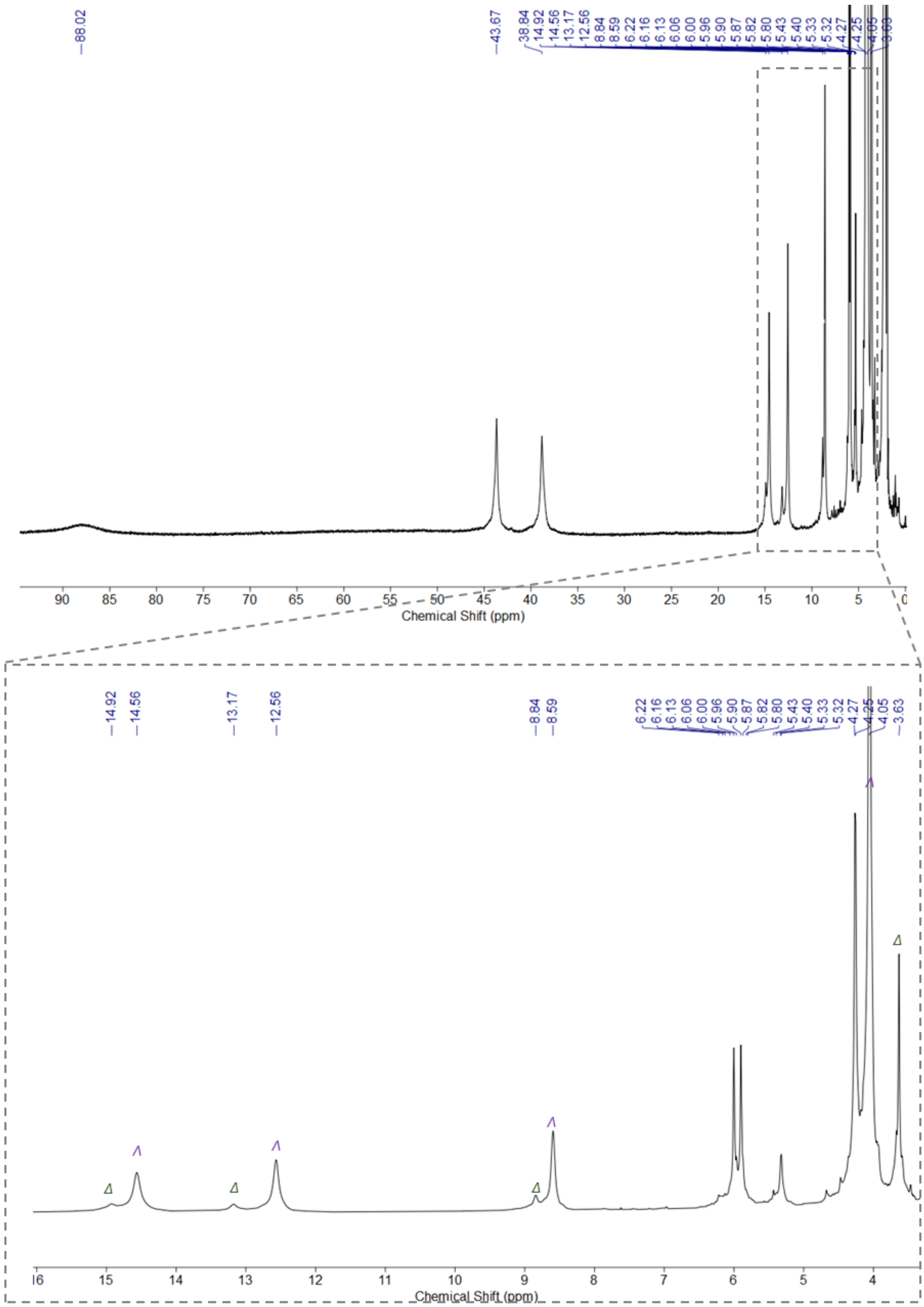

Figure S22. Wide sweep ^{1}H NMR spectrum of cage **2** (500 MHz, CD_3CN, 25 °C).

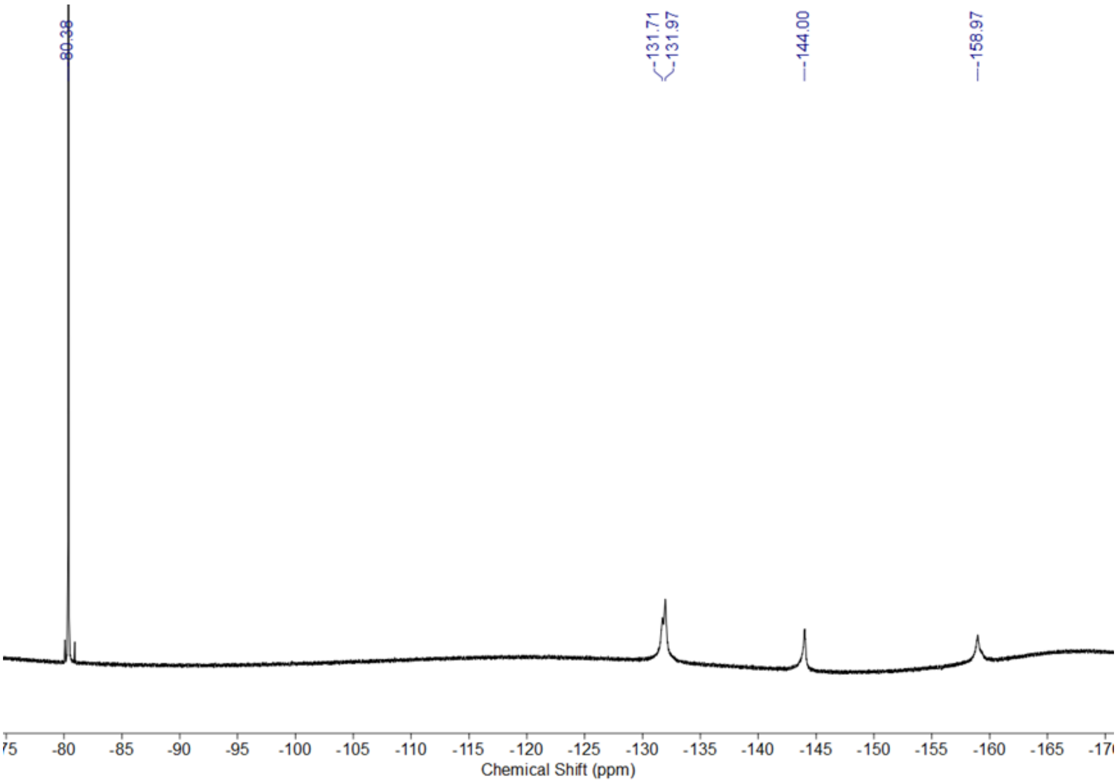

Figure S23. ^{19}F NMR spectrum of cage **2** (376 MHz, CD_3CN, 25 °C).

4 Investigation of Diastereocontrol for Cage 1

4.1 Solvent Effect

Self-assembly in single solvent. Ligand **A** (2.06 mg, 1.0 μmol, 1.0 equiv) and $Zn(NTf_2)_2$ (0.84 mg, 1.34 μmol, 1.34 equiv) were parallelly combined in the corresponding deuterated solvents (0.5 mL) in NMR tubes. The reaction mixtures were heated at 70 °C for 2 hours. After cooling down to room temperature, ^{1}H NMR spectra were measured within 4 hours.

In $CDCl_3$, CD_3OD, CD_3SOCD_3 and $(CD_3)_2NCOD$, cage **1** was not formed; in CD_3CN, CD_3NO_2 and CD_3COCD_3, cage **1** was formed, confirmed by ^{1}H NMR and low-resolution ESI-MS. Different diastereoselectivities were observed dependent on reaction solvents (Table S1 and Figure S24). In CD_3CN, $(S)_{24}$-Λ_8-**1** was predominantly formed, while $(S)_{24}$-Δ_8-**1** was the major diastereomer in both CD_3NO_2 and CD_3COCD_3. The diastereomeric ratios were determined by ^{1}H NMR integration. Stereochemical outcomes were further confirmed by CD spectra (Figure S25). Cage **1** formed in MeCN displayed negative Cotton effects corresponding to Soret and Q bands, whereas positive signals were observed for cage **1** in both $MeNO_2$ and acetone.

Table S1. Self-Assembly of Cage 1 in Different Solvents.

Entry	Reaction Condition	Solvent	d.r. = $(S)_{24}$-Λ_8-**1**:$(S)_{24}$-Δ_8-**1**
1	70 °C, 2h	CD_3CN	3.8:1
2	70 °C, 2h	CD_3NO_2	1:6
3	70 °C, 2h	CD_3COCD_3	1:2.4
4	70 °C, 2h	$CDCl_3$	no self-assembly
5	70 °C, 2h	CD_3OD	no self-assembly
6	70 °C, 2h	$(CD_3)_2NCOD$	no self-assembly
7	70 °C, 2h	CD_3SOCD_3	no self-assembly

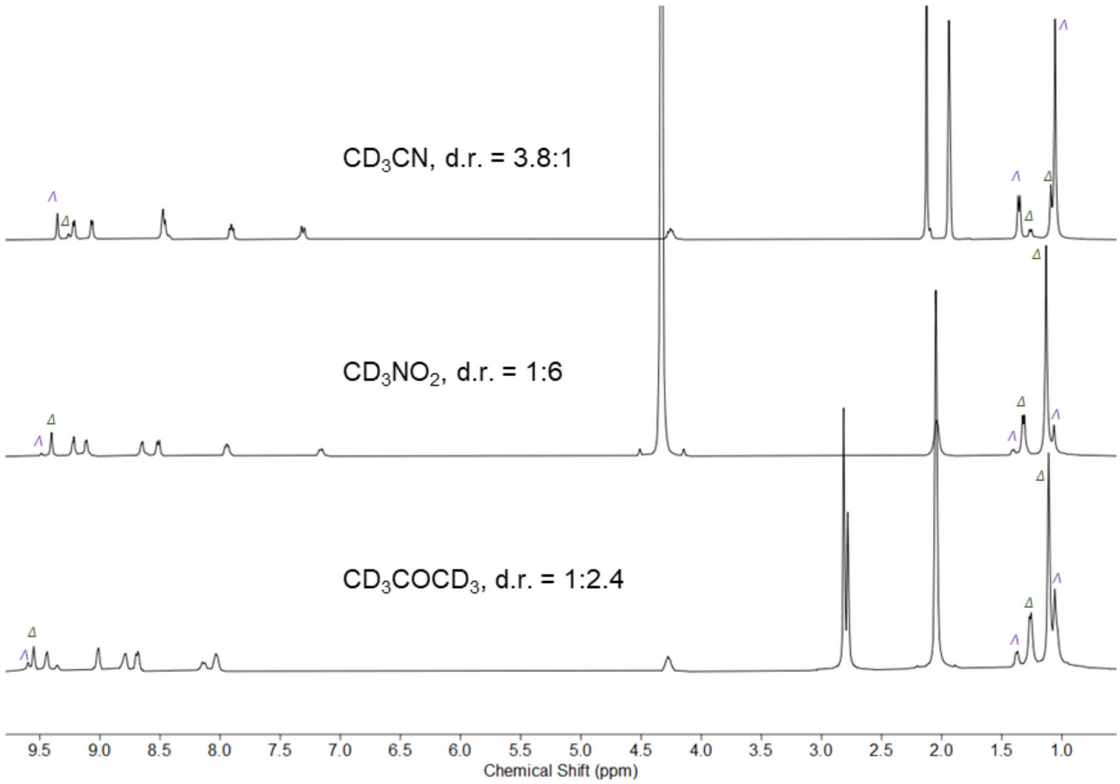

Figure S24. ^{1}H NMR spectra of cage **1** formed in different solvents (400 MHz, 25 °C)

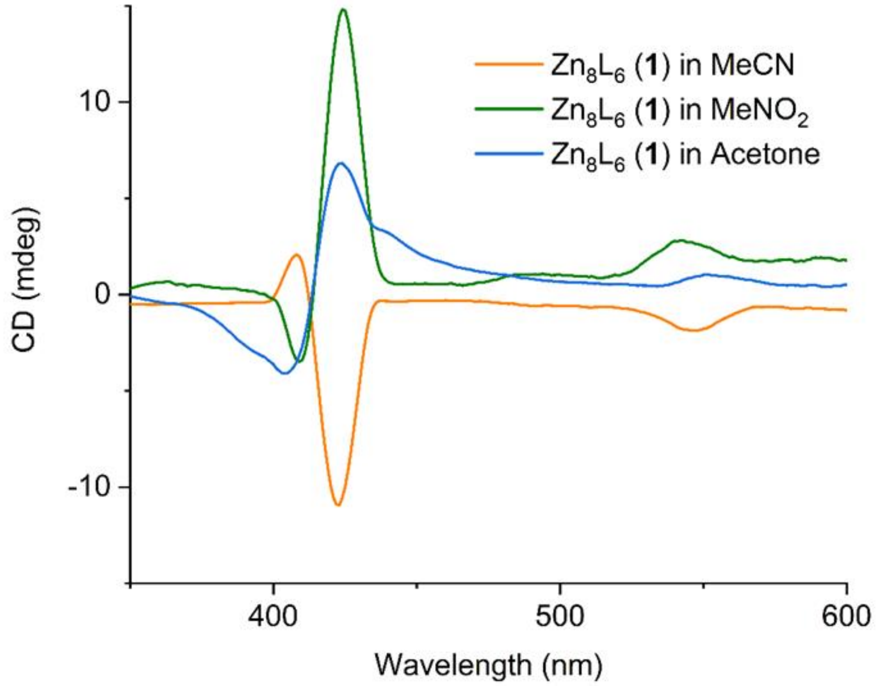

Figure S25. CD spectra of cage **1** in different solvents for solutions of equal concentration.

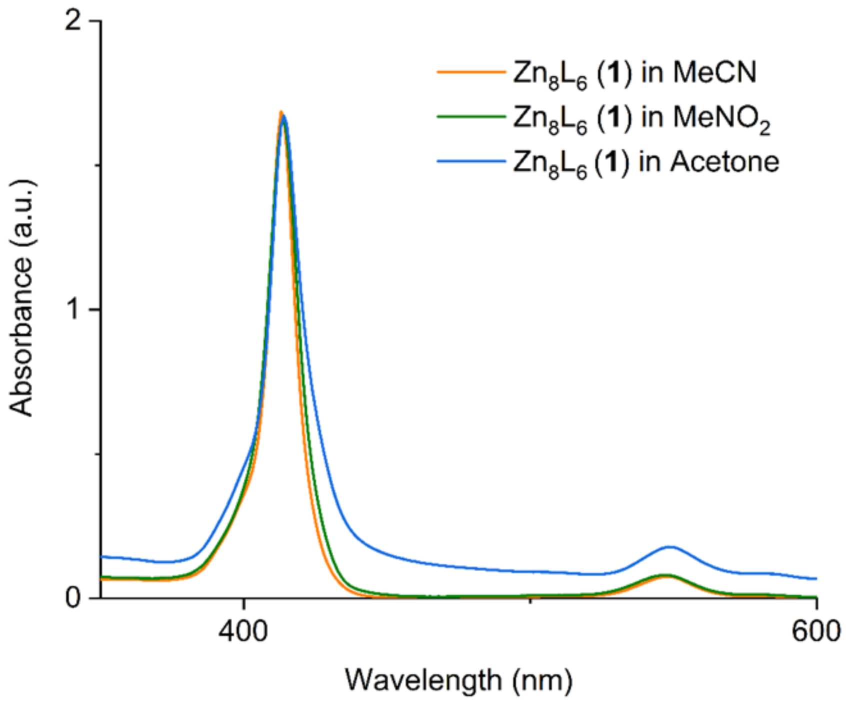

Figure S26. UV/Vis spectra of cage **1** in different solvents.

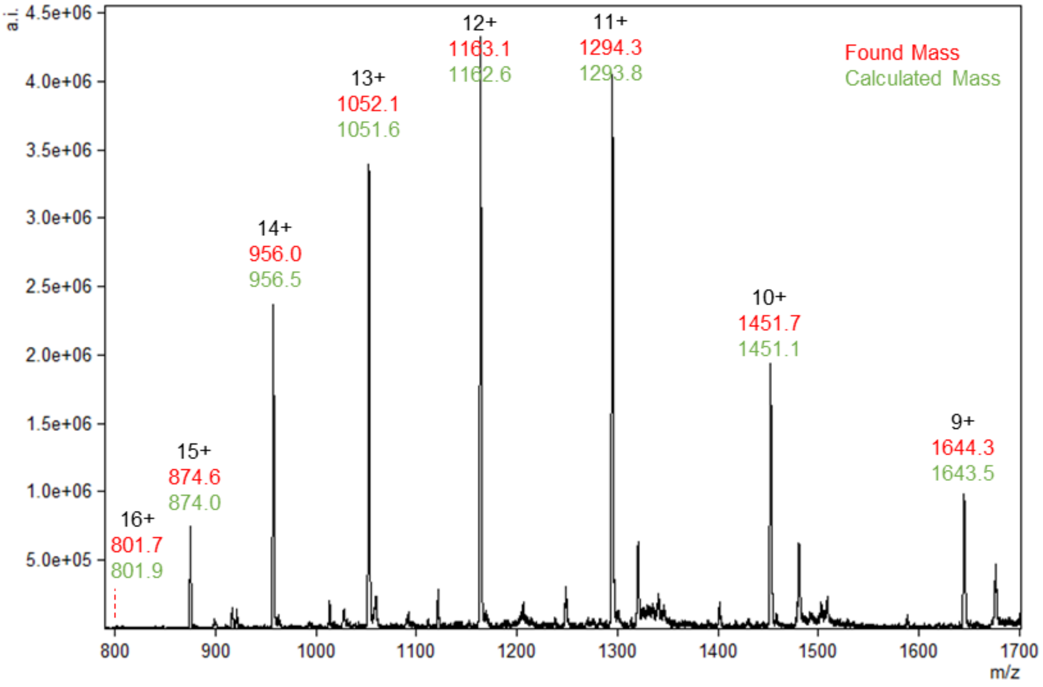

Figure S27. Low-resolution ESI-MS spectrum of cage **1** in $MeNO_2$.

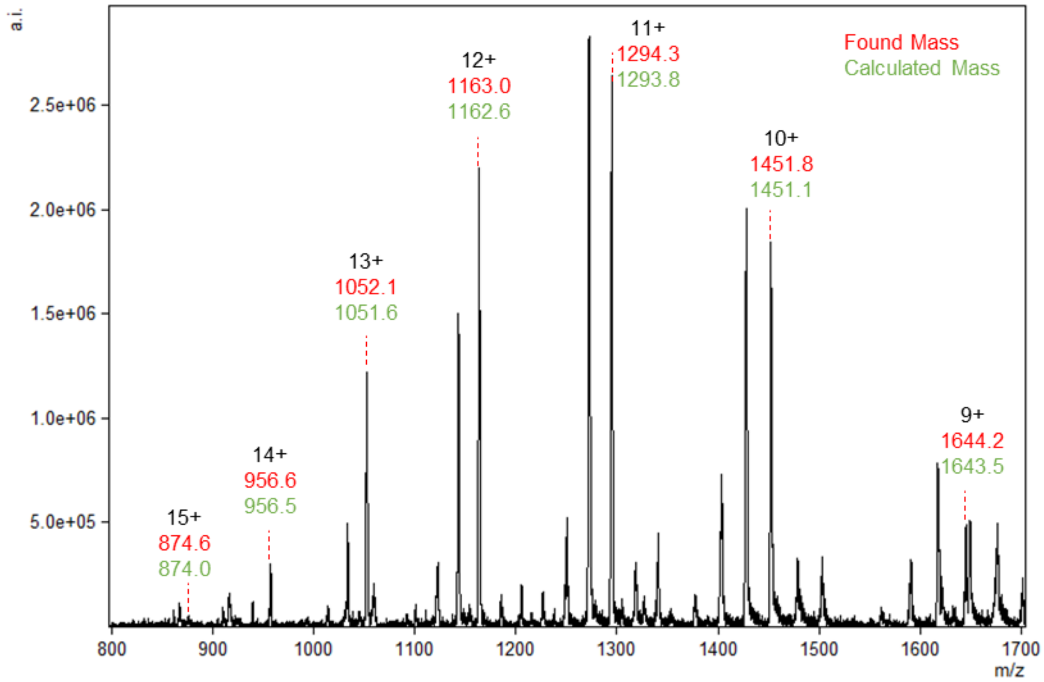

Figure S28. Low-resolution ESI-MS spectrum of cage **1** in Acetone. Fragments correspond to the loss of a porphyrin metal center under test conditions.

Self-assembly in mixed solvent. Ligand **A** (2.06 mg, 1.0 μmol, 1.0 equiv) and $Zn(NTf_2)_2$ (0.84 mg, 1.34 μmol, 1.34 equiv) were parallelly combined in mixed solvents (0.5 mL) with different ratios of CD_3CN/CD_3NO_2 in NMR tubes. The reaction mixtures were heated at 70 °C for 2 hours. After cooling down to room temperature, ^{1}H NMR spectra were measured within 4 hours.

It was observed that diastereomeric ratios differed with the ratio of CD_3CN/CD_3NO_2 (Figure S29). In general, *Λ*-Zn centers are favored in CD_3CN while *Δ*-Zn centers are preferred in CD_3NO_2. Cage **1** with NTf_2^- as counter anion is highly soluble in both acetonitrile and nitromethane; both solvents have similar polarities. Hence, the solubility and polarity are unlikely to account for the solvent-dependent diastereoselectivity.

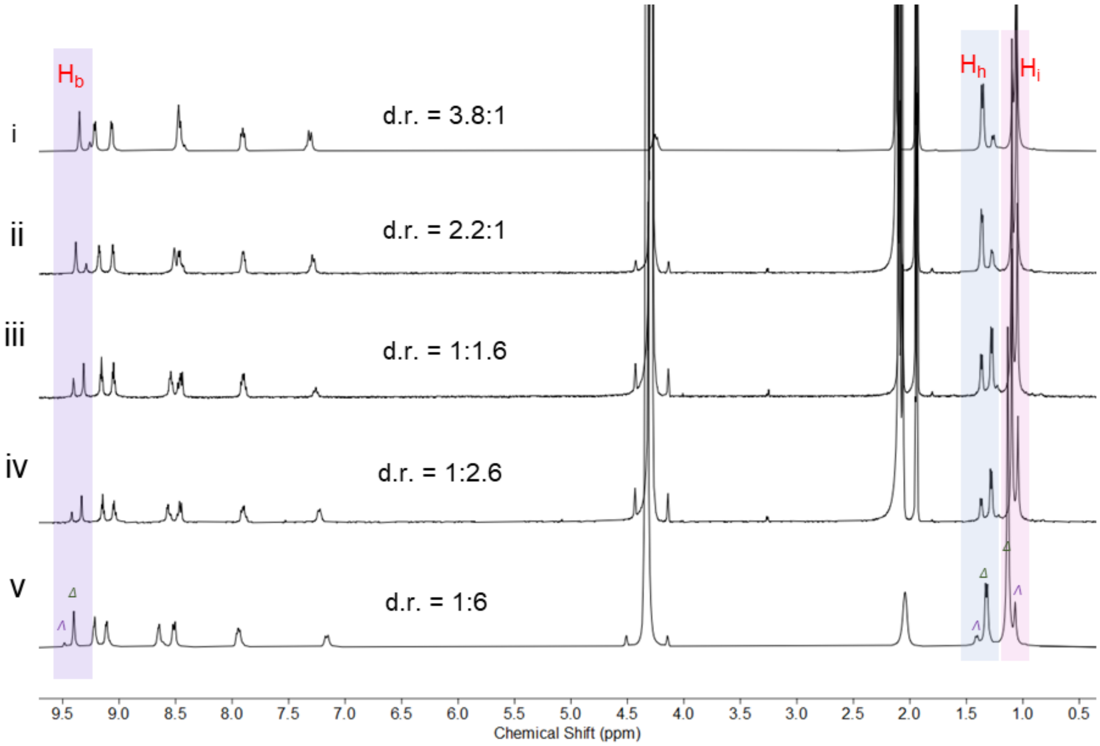

Figure S29. ^{1}H NMR spectra of cage **1** formed in solvent mixtures. (i) CD_3CN (400 MHz, 25 °C); (ii) $CD_3CN:CD_3NO_2$ = 7:3 (500 MHz, 25 °C); (iii) $CD_3CN:CD_3NO_2$ = 1:1 (500 MHz, 25 °C); (iv) $CD_3CN:CD_3NO_2$ = 3:7 (500 MHz, 25 °C); (v) CD_3NO_2 (400 MHz, 25 °C).

Solvent exchange experiments. *Step 1:* Cage **1** (2.60 mg, 0.15 μmol) was dissolved in CD_3CN (0.5 mL) in an NMR tube, and the ^1H NMR spectrum was measured (d.r. = 3.8:1). *Step 2:* CD_3CN was evaporated and CD_3NO_2 (0.5 mL) was added; the ^1H NMR spectrum was then measured (d.r. = 3.8:1) after being kept at 25 °C for 10 minutes. *Step 3:* The cage solution was heated at 70 °C for 2 hours; the ^1H NMR spectrum was then measured (d.r. = 1:6). *Step 4:* CD_3NO_2 was evaporated and CD_3CN was readded; the ^1H NMR spectrum was then measured (d.r. = 3.8:1) after being kept at 25 °C for 10 minutes.

It was observed that the diastereoselectivity could be easily switched by changing solvents (Figure S30). These observations indicated that the (*S*)$_{24}$-Λ_8-**1**$\rightleftarrows$(*S*)$_{24}$-Δ_8-**1** interconversion is a reversible process controlled by the solvent.

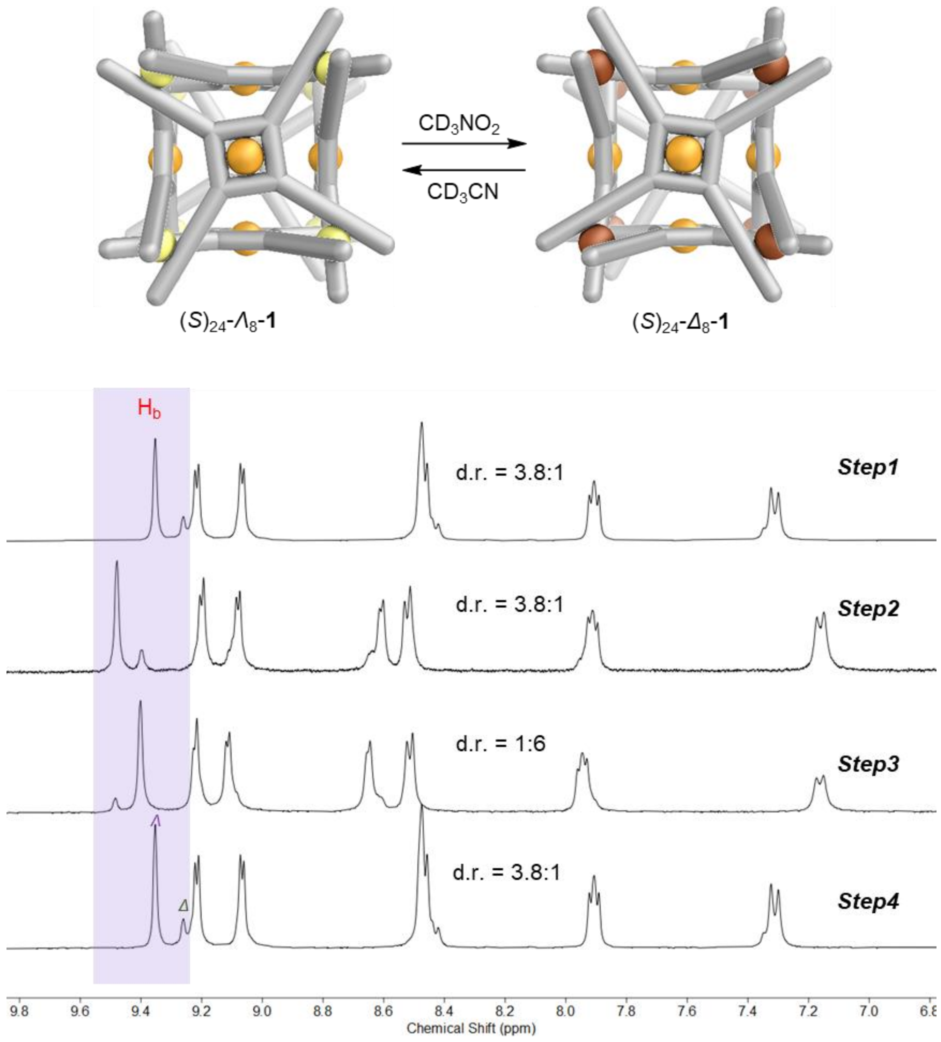

Figure S30. Reversible diastereoselectivities controlled by solvent.

Characterization of Cage 1 in CD_3NO_2:

^{1}H NMR (700 MHz, CD_3NO_2): δ 1.07 (s, 31H, (S)$_{24}$-Λ_8-**1**), 1.13 (s, 185H, (S)$_{24}$-Δ_8-**1**),1.32 (d, J = 6.7 Hz, 10.3H, (S)$_{24}$-Δ_8-**1**), 1.36 (d, J = 6.7 Hz, 61.7 H, (S)$_{24}$-Λ_8-**1**), 4.31–4.35 (m, 24H, (S)$_{24}$-Λ_8-**1**+(S)$_{24}$-Δ_8-**1**, overlaid with CD_3NO_2), 7.11–7.23 (m, 24H, (S)$_{24}$-Λ_8-**1**+(S)$_{24}$-Δ_8-**1**), 7.87–8.00 (m, 24H, (S)$_{24}$-Λ_8-**1**+(S)$_{24}$-Δ_8-**1**), 8.45–8.55 (m, 24H, (S)$_{24}$-Λ_8-**1**+(S)$_{24}$-Δ_8-**1**), 8.58–8.72 (m, 24H, (S)$_{24}$-Λ_8-**1**+(S)$_{24}$-Δ_8-**1**), 9.02–9.13 (m, 24H, (S)$_{24}$-Λ_8-**1**+(S)$_{24}$-Δ_8-**1**), 9.15–9.26 (m, 24H, (S)$_{24}$-Λ_8-**1**+(S)$_{24}$-Δ_8-**1**), 9.40 (d, J = 2.2 Hz, 5H, (S)$_{24}$-Δ_8-**1**), 9.48 (d, J = 2.2 Hz, 19 H, (S)$_{24}$-Λ_8-**1**) ppm. **UV/Vis** ($MeNO_2$): λ_{max} 414 nm (Soret band), λ_{max} 546 nm (Q band). **ESI-MS**: m/z = 801.7 [**1**-16(NTf_2)]$^{16+}$, 874.6 [**1**-15(NTf_2)]$^{15+}$, 956.0 [**1**-14(NTf_2)]$^{14+}$, 1052.1 [**1**-13(NTf_2)]$^{13+}$, 1163.1 [**1**-12(NTf_2)]$^{12+}$, 1294.3 [**1**-11(NTf_2)]$^{11+}$, 1451.7 [**1**-10(NTf_2)]$^{10+}$,1644.3 [**1**-9(NTf_2)]$^{9+}$.

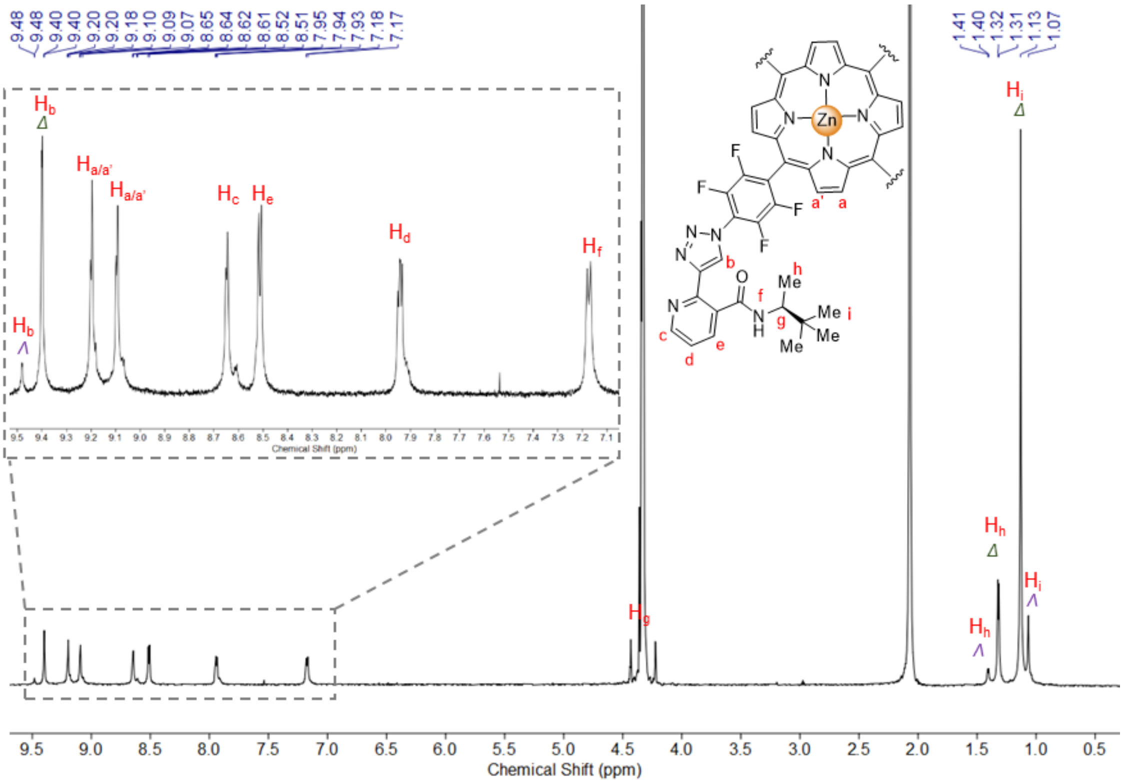

Figure S31. ^{1}H NMR spectrum of cage **1** (700 MHz, CD_3NO_2, 25 °C).

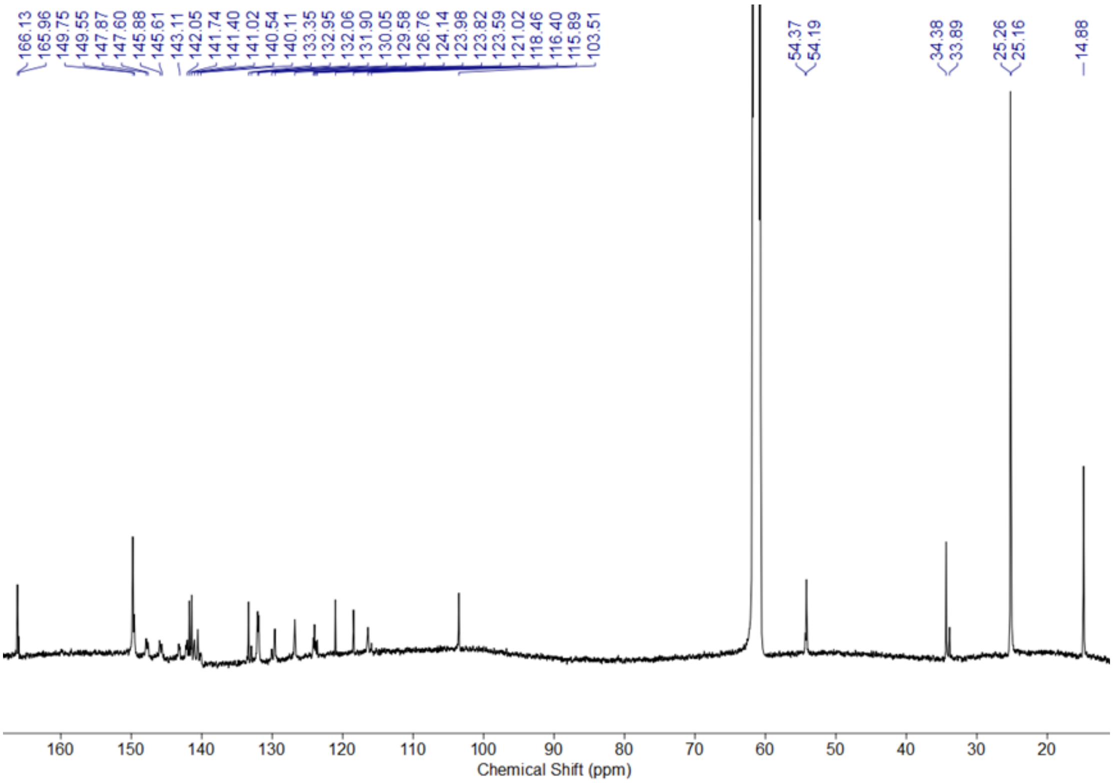

Figure S32. ^{13}C NMR spectrum of cage **1** (126 MHz, CD_3NO_2, 25 °C).

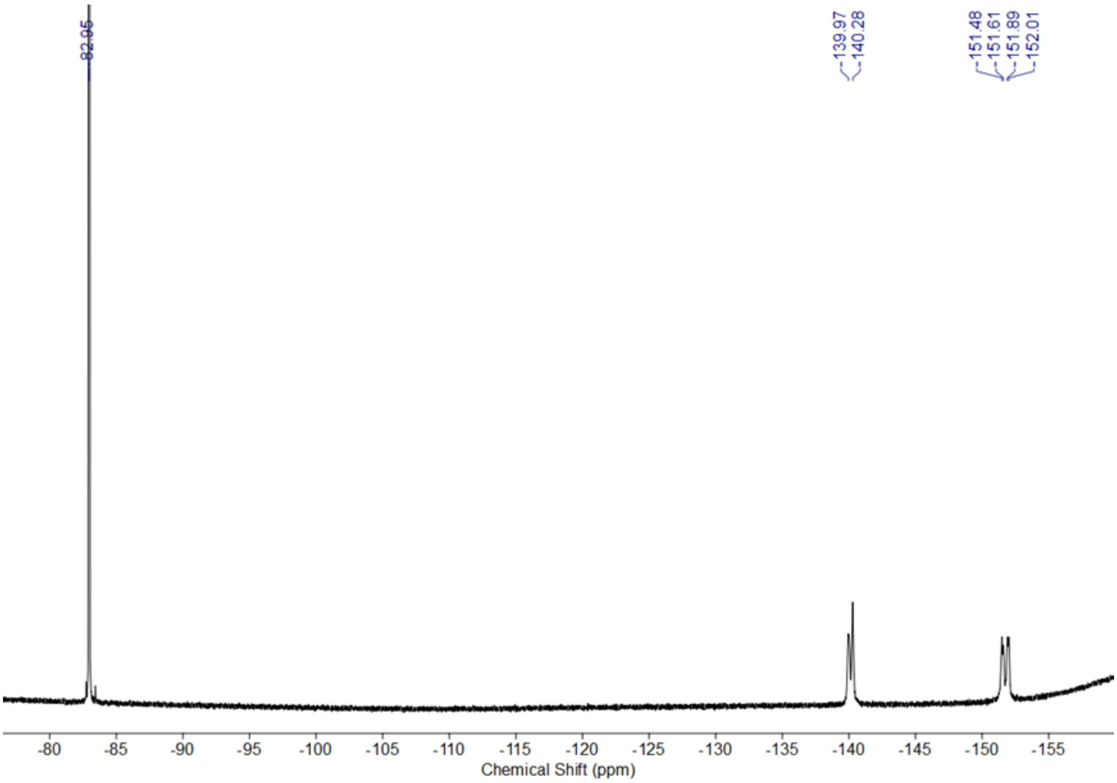

Figure S33. ^{19}F NMR spectrum of cage **1** (470 MHz, CD_3NO_2, 25 °C).

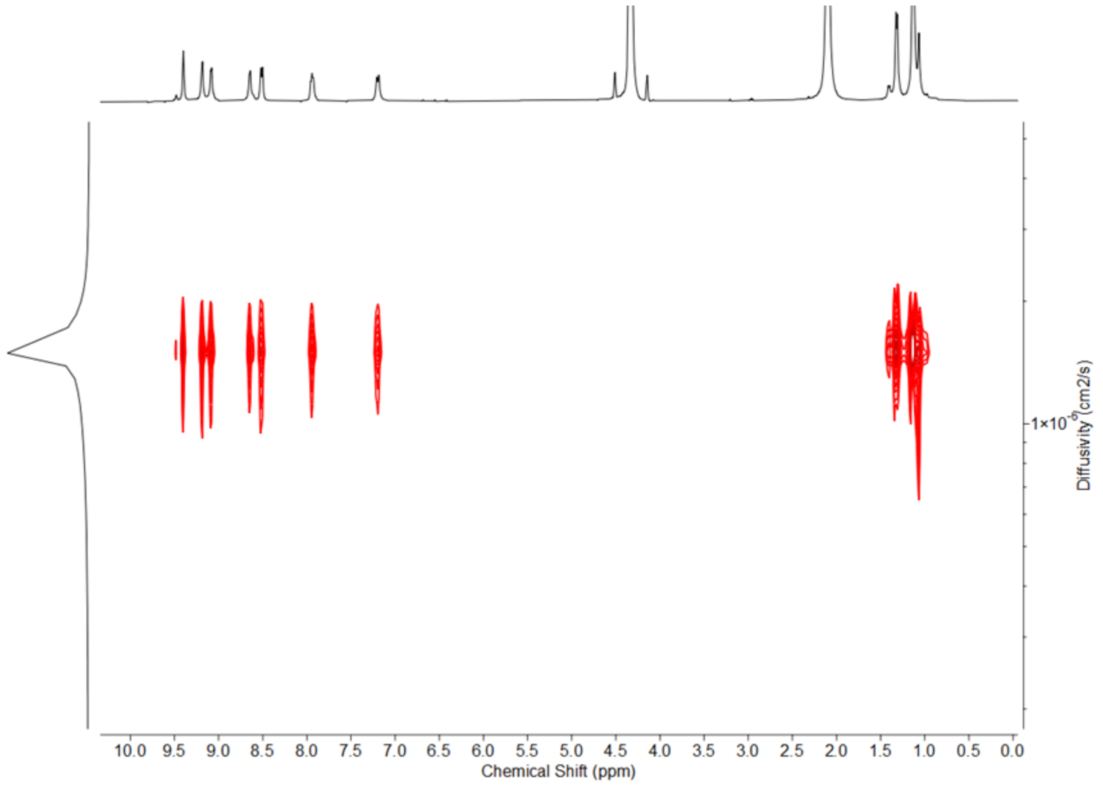

Figure S34. ^{1}H DOSY spectrum of cage **1** (400 MHz, CD_3NO_2, 25 °C). The diffusion coefficient for both diastereomers in CD_3NO_2 was measured to be 1.52×10^{-6} cm^2/s.

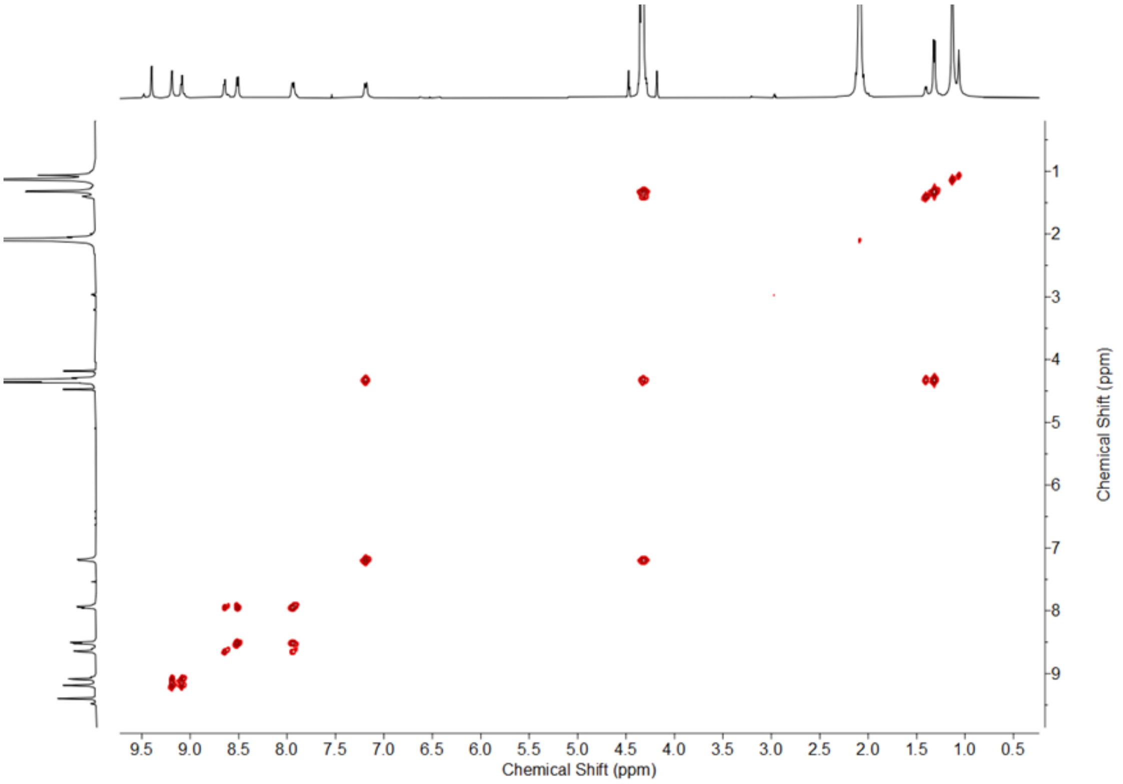

Figure S35. ^{1}H-^{1}H COSY NMR spectrum of cage **1** (500 MHz, CD_3NO_2, 25 °C).

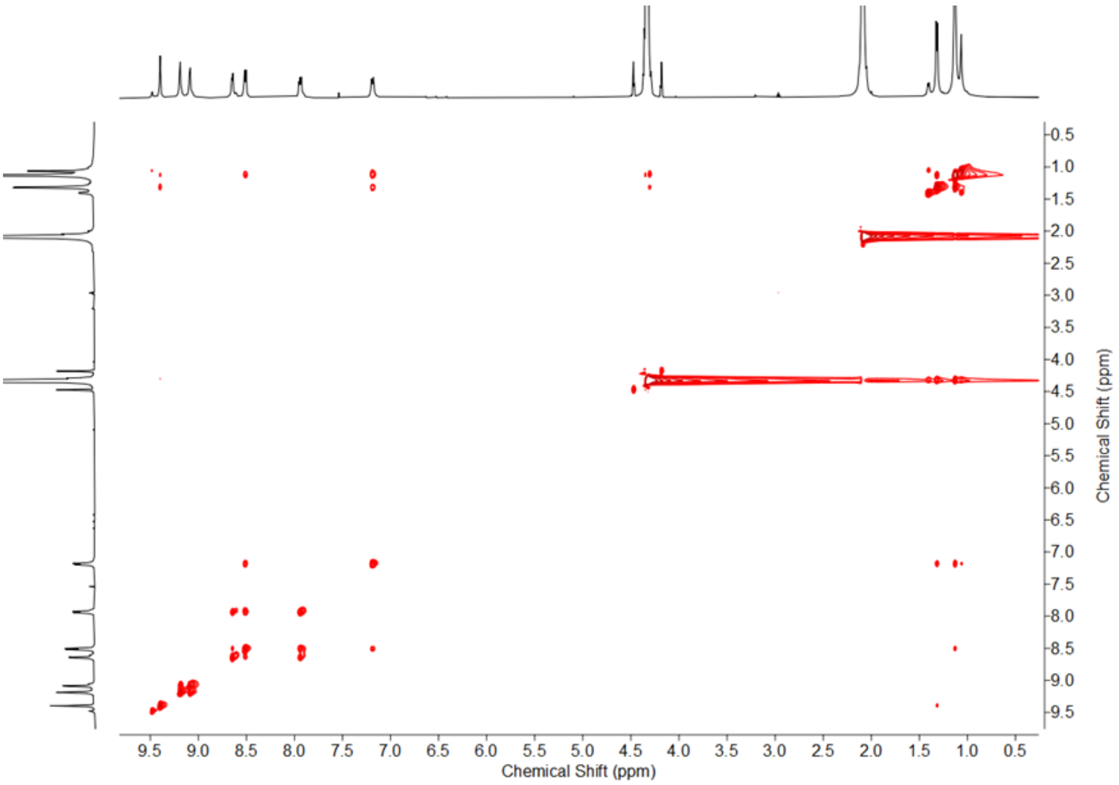

Figure S36. ^{1}H-^{1}H NOESY NMR spectrum of cage **1** (500 MHz, CD_3NO_2, 25 °C).

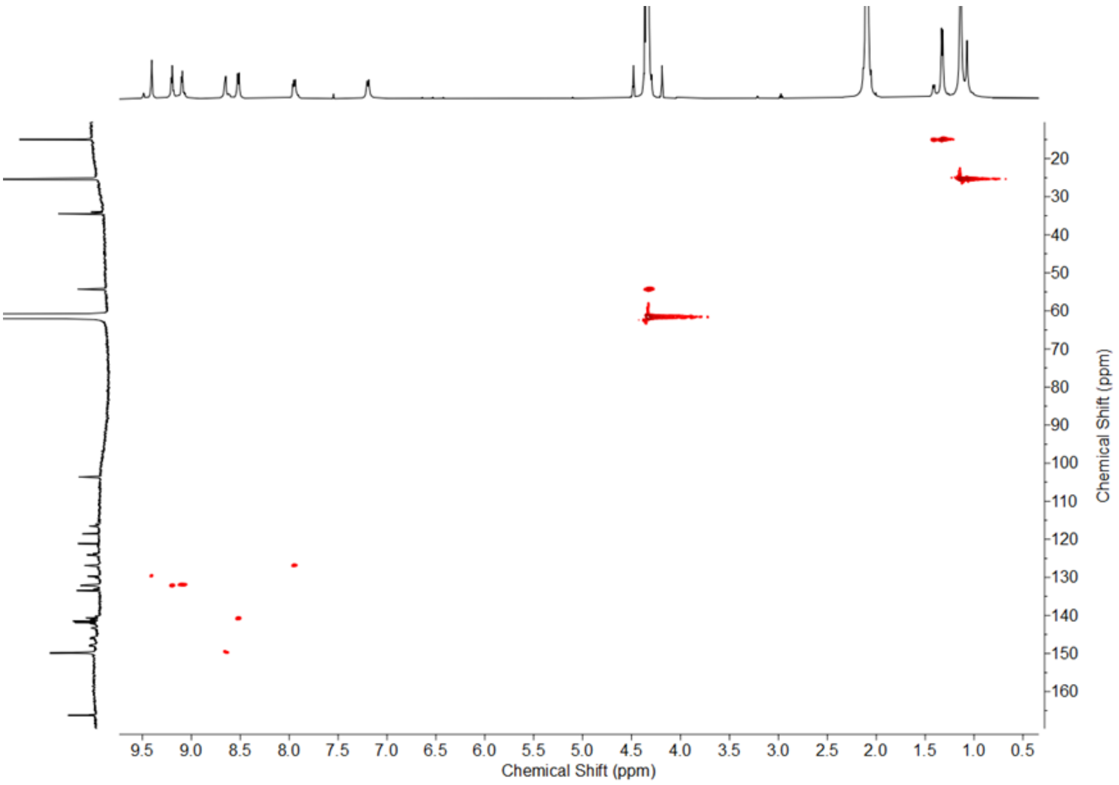

Figure S37. ^{1}H-^{13}C HSQC NMR spectrum of cage **1** (500 MHz, CD_3NO_2, 25 °C).

4.2 Temperature Effect

The self-assembly of ligand **A** (2.06 mg, 1.0 μmol, 1.0 equiv) with $Zn(NTf_2)_2$ (0.84 mg, 1.34 μmol, 1.34 equiv) were parallelly run at different temperatures (80°C, 100°C and 120 °C) in an oil bath in NMR tubes. After cooling down to room temperature, ^{1}H NMR spectra were measured within 4 hours. In both CD_3NO_2 and CD_3CN, the same diastereoselectivities were observed as the previous results (d.r. = 3.8:1 in CD_3CN and d.r. = 1:6 in CD_3NO_2) at 70 °C.

Cage was formed in acetonitrile with a diastereomeric ratio of $(S)_{24}$-Λ_8-**1**:$(S)_{24}$-Δ_8-**1** = 3.8:1. The acetonitrile solution of cage **1** could be stored at 25 °C for six months with no changes in the diastereomeric ratio, and no decomposition was observed for the cage.

Cage **1** was formed in nitromethane with a diastereomeric ratio of $(S)_{24}$-Λ_8-**1**:$(S)_{24}$-Δ_8-**1** = 1:6. However, after being kept at 25 °C for 7 days, a proportion of $(S)_{24}$-Δ_8-**1** converted into $(S)_{24}$-Λ_8-**1** reaching an equilibrium with a diastereomeric ratio of $(S)_{24}$-Λ_8-**1**:$(S)_{24}$-Δ_8-**1** = 1:1.5. Reequilibration back to the original diastereomeric ratio of 1:6 could be achieved by heating the diastereomeric cage mixtures.

Having identified the temperature-dependent equilibrium of $(S)_{24}$-Λ_8-**1**$\rightleftharpoons$$(S)_{24}$-$\Delta_8$-**1** in nitromethane, variable temperature ^{1}H NMR experiments ranging from 25 °C to 85 °C were carried out (Figure S38). At 25 °C, the diastereomeric ratio of $(S)_{24}$-Λ_8-**1**:$(S)_{24}$-Δ_8-**1** was 1:1.5. After being heated to 85 °C, the diastereomeric ratio of $(S)_{24}$-Λ_8-**1**:$(S)_{24}$-Δ_8-**1** was rebalanced to 1:6, and no further changes were observed at 100 °C. No decomposition was observed for the cage throughout the whole temperature range. Based on the variable temperature ^{1}H NMR experiments, van ’t Hoff equation analysis was conducted to gain mechanistic insight into the interconversion of $(S)_{24}$-Λ_8-**1** and$(S)_{24}$-Δ_8-**1** in nitromethane (Figure 39). The results revealed that the conversion of $(S)_{24}$-Λ_8-**1** into $(S)_{24}$-Δ_8-**1** in nitromethane is an endothermic and entropically favored process with ΔH = 24.6 ± 0.8 kJ mol^{-1} and ΔS = 83.1 ± 2.4 J K^{-1} mol^{-1}.

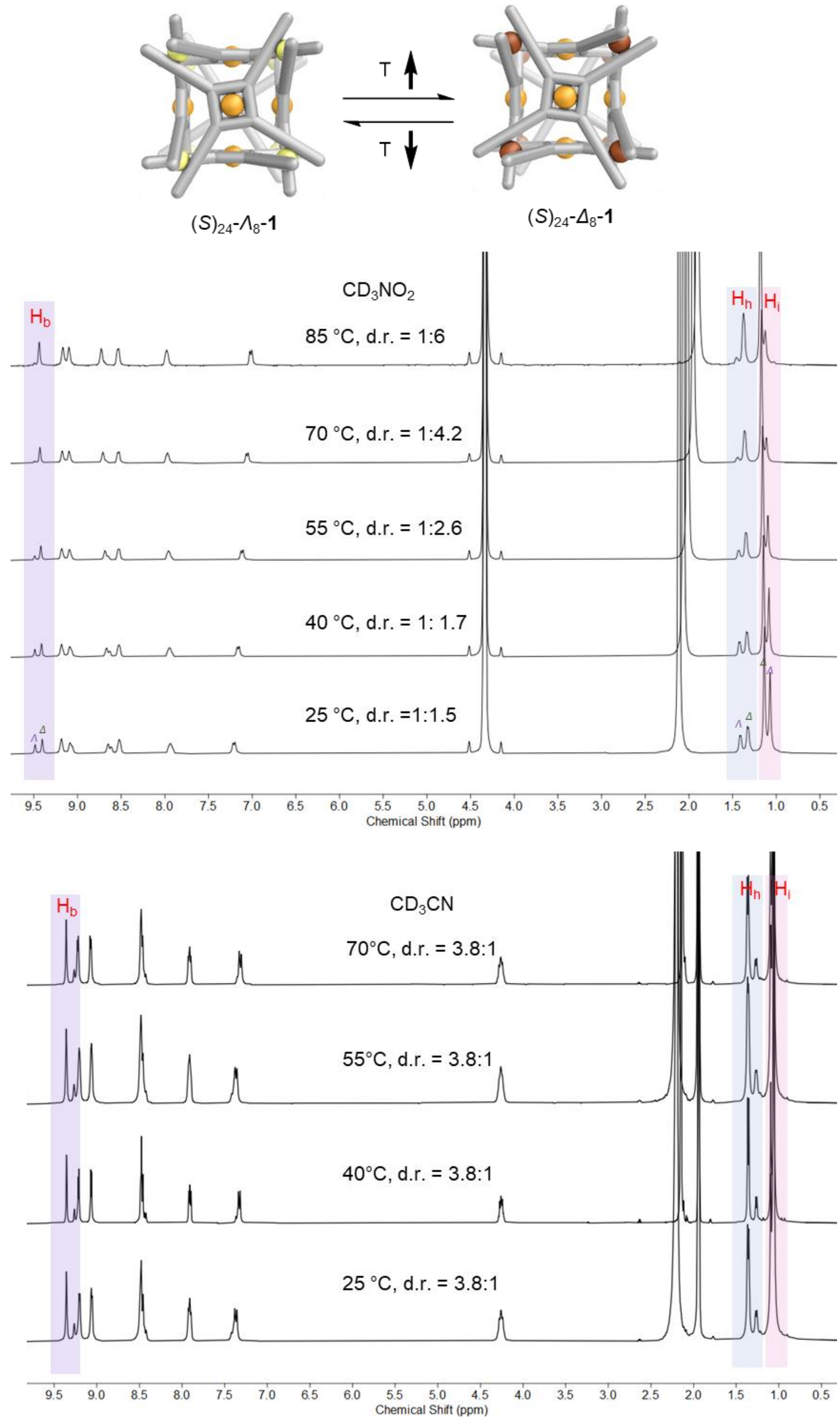

Figure S38. ^{1}H NMR spectra (400 MHz) of cage **1** at different temperatures in both CD_3NO_2 (400 MHz) and CD_3CN (400 MHz).

In solution at a particular temperature (T), the equilibrium between (S)$_{24}$-Λ_8-**1** and (S)$_{24}$-Δ_8-**1** is described by the following equation

$$(S)_{24}\text{-}\Lambda_8\text{-}\mathbf{1} \leftrightharpoons (S)_{24}\text{-}\Delta_8\text{-}\mathbf{1}$$

The equilibrium constant K_{eq} at this temperature is described by the following equation

$$K_{eq} = [(S)_{24}\text{-}\Delta_8\text{-}\mathbf{1}]/[(S)_{24}\text{-}\Lambda_8\text{-}\mathbf{1}]$$

K_{eq} can be calculated by the ratio of (S)$_{24}$-Δ_8-**1**:(S)$_{24}$-Λ_8-**1** at each temperature (T). A linear regression of these data was then undertaken using an integrated form of the van't Hoff equation, where

$$\Delta G = \Delta H\text{-}T\Delta S$$

$$\Delta G = \text{-}RT \ln K_{eq}$$

and thus

$$\ln K_{eq} = \text{-}\Delta H/RT + \Delta S/R$$

where R is the ideal gas constant (8.314 J mol^{-1}), ΔH is the change in enthalpy and ΔS is the change in entropy.

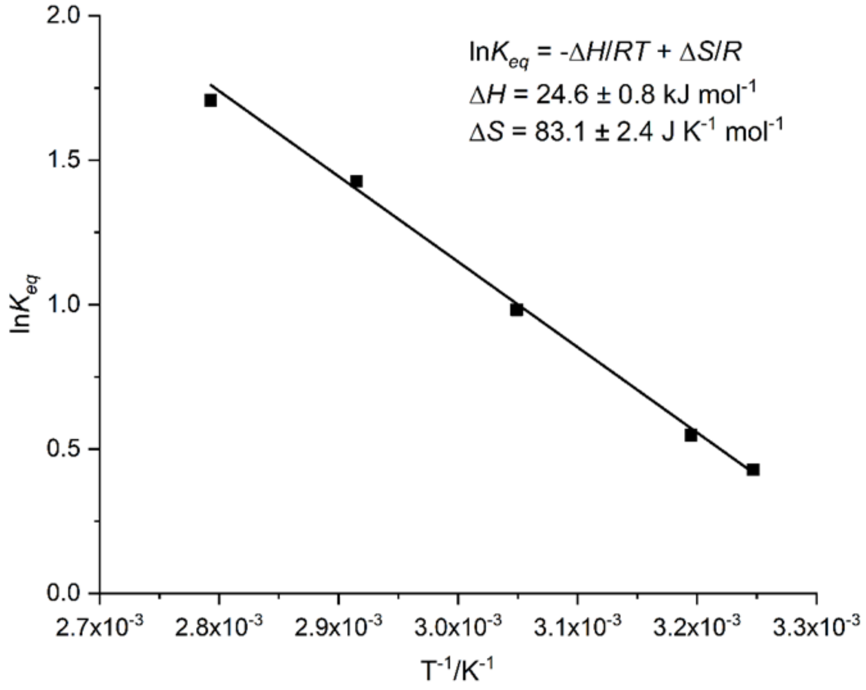

Figure S39. van ’t Hoff analysis of the equilibrium for the interconversion between (S)$_{24}$-Δ_8-**1** and (S)$_{24}$-Λ_8-**1**.

4.3 Concentration Effect

Ligand **A** (1.0 equiv) and $Zn(NTf_2)_2$ (1.34 equiv) were parallelly combined in the corresponding deuterated solvents (0.5 mL) in NMR tubes. The reaction mixtures were heated at 70 °C for 2 hours. After cooling down to room temperature, ^{1}H NMR spectra were measured within 4 hours.

At different ligand concentrations (1 to 8 mM), the same diastereomeric ratio (d.r. = 3.8:1) was observed for the formation of cage **1** in CD_3CN (Figure S40). At different concentrations (1 to 8 mM), the same diastereomeric ratio (d.r. = 1:6) was also observed for the formation of cage **1** in CD_3NO_2 (Figure S41)

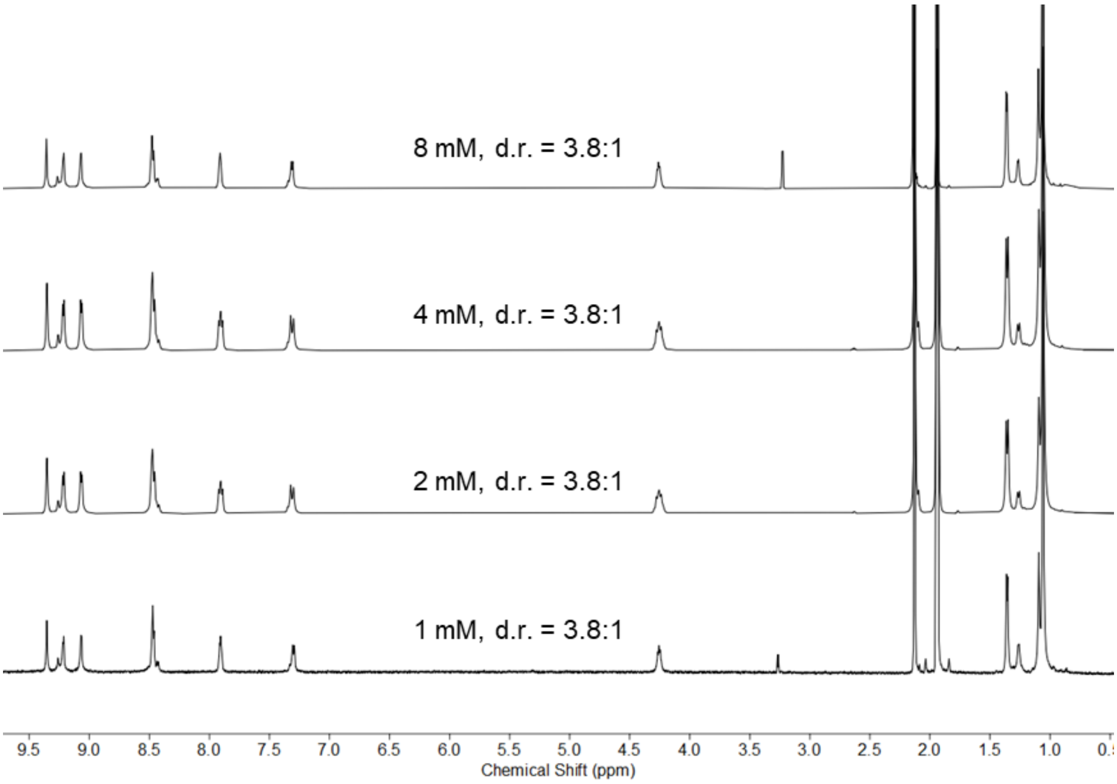

Figure S40. ^{1}H NMR spectra (CD_3CN) of cage **1** formed at different concentrations.

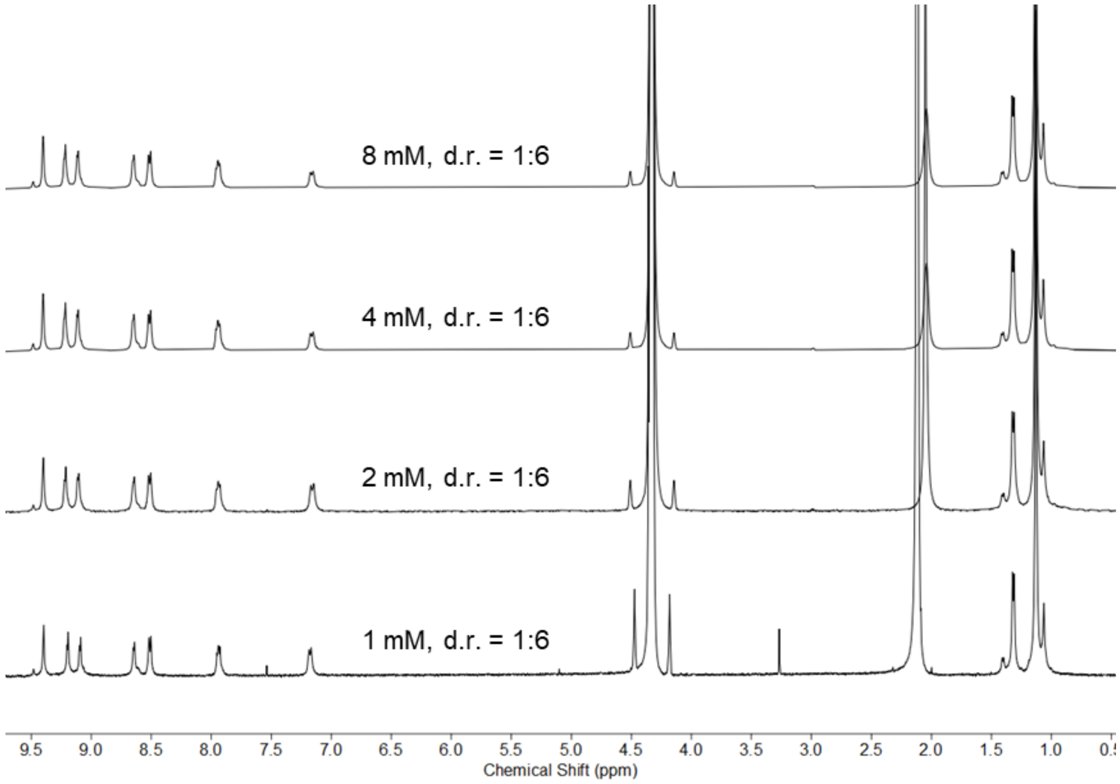

Figure S41. ^{1}H NMR spectra (CD_3NO_2) of cage **1** formed at different concentrations.

5 Host-Guest Studies Using Cage 1

5.1 Encapsulation of Guest 1

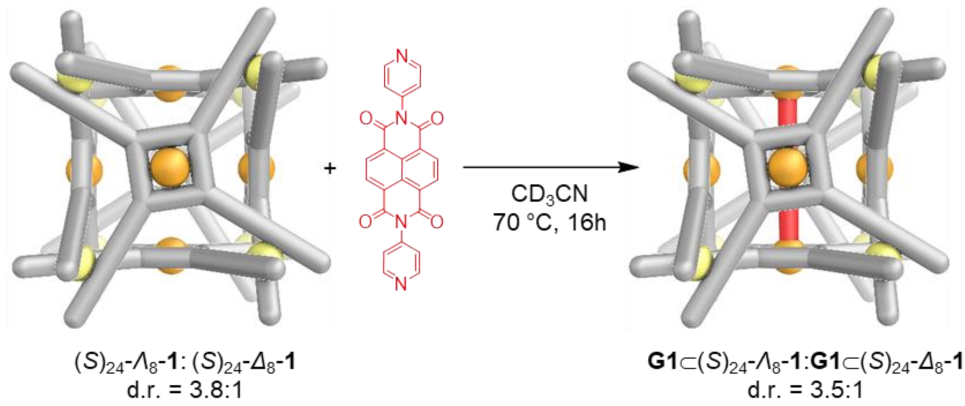

Cage **1** (17.3 mg, 1.0 μmol, 1.0 equiv) and 4,4′-dipyridyl naphthalenediimide (**G1**, 0.42 mg, 1.0 μmol, 1.0 equiv) were combined in MeCN (3 mL) in a 15 mL tube. The reaction mixture was stirred at 70 °C for 16 hours. The solvent was reduced to 1 mL, and Et_2O (10 mL) was then added. The precipitate was collected by centrifugation and washed with excess diethyl ether, affording the host-guest complex **G1**⊂**1** as a purple solid (16.5 mg, 93%).

G1⊂**1** consists of a pair of diastereomers. According to the ^{1}H NMR spectrum, the accurate diastereomeric ratio (d.r.) was determined to be 3.5:1. Negative Cotton effects were observed in CD spectrum (Figure S50), which is in line with the observations of parent cage **1** in MeCN (Figure S25). The major diastereomer was considered to be **G1**⊂(S)$_{24}$-Λ_8-**1**, while the minor diastereomer is **G1**⊂(S)$_{24}$-Δ_8-**1**.

^{1}H NMR (500 MHz, CD_3CN): δ 0.98–1.15 (m, 216H), 1.21–1.29 (m, 16H, **G1**⊂(S)$_{24}$-Δ_8-**1**), 1.30–1.40 (m, 56H, **G1**⊂(S)$_{24}$-Λ_8-**1**), 1.93-1.95 (4H, overlaid with CD_3CN), 4.15–4.35 (m, 24H), 4.61–4.69 (m, 4H), 6.00 (s, 3.1H, **G1**⊂(S)$_{24}$-Λ_8-**1**), 6.03 (s, 0.9H, **G1**⊂(S)$_{24}$-Δ_8-**1**), 7.24–7.41 (m, 24H), 7.85–7.98 (m, 24H), 8.38–8.63 (m, 48H), 8.90–9.48 (m, 72H) ppm. **UV/Vis** (MeCN): λ_{max} 414 nm (Soret band), λ_{max} 549 nm (Q band). **ESI-MS**: m/z = 828.0 [**G1**⊂**1**-16(NTf_2)]$^{16+}$, 901.8 [**G1**⊂**1**-15(NTf_2)]$^{15+}$, 986.4 [**G1**⊂**1**-14(NTf_2)]$^{14+}$, 1083.7 [**G1**⊂**1**-13(NTf_2)]$^{13+}$, 1197.4 [**G1**⊂**1**-12(NTf_2)]$^{12+}$, 1331.6 [**G1**⊂**1**-11(NTf_2)]$^{11+}$, 1492.8 [**G1**⊂**1**-10(NTf_2)]$^{10+}$,1690.2 [**G1**⊂**1**-9(NTf_2)]$^{9+}$.

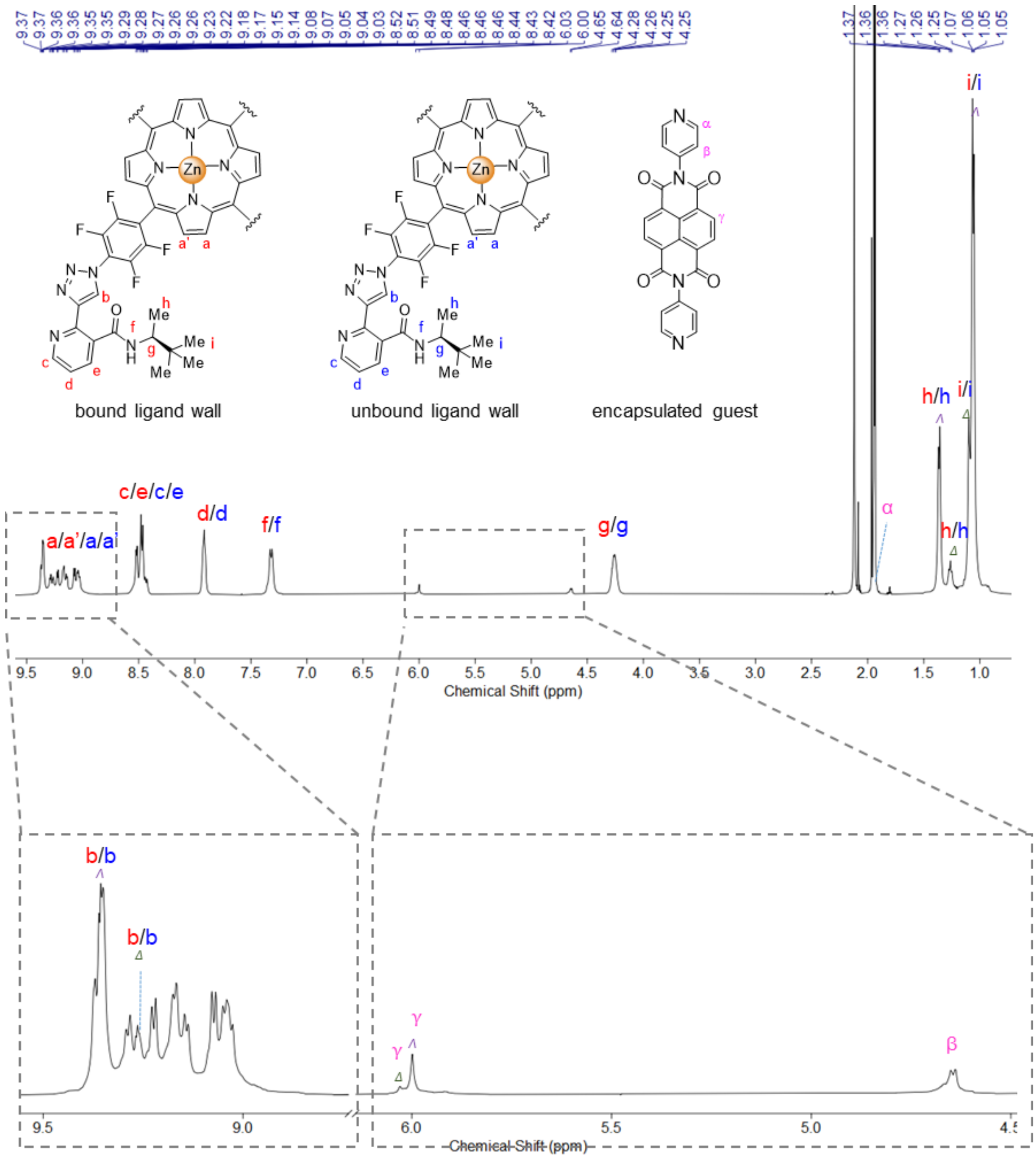

Figure S42. ^1H NMR spectrum of **G1⊂1** (500 MHz, CD_3CN, 25 °C).

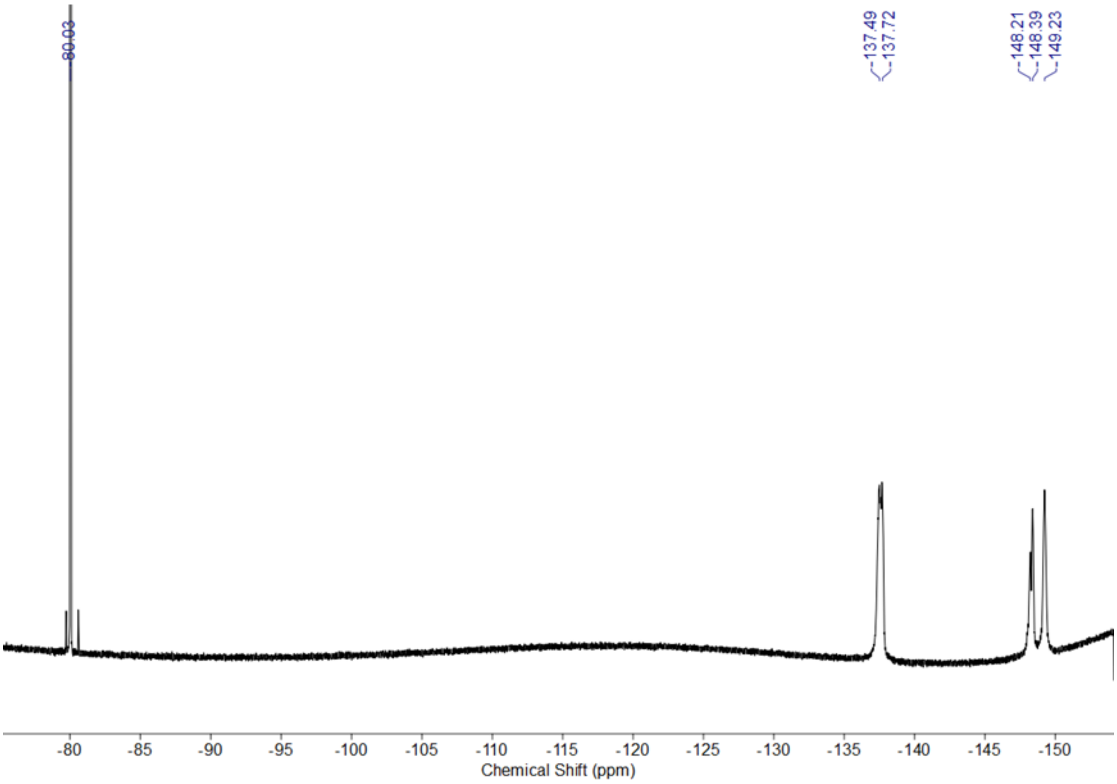

Figure S43. ^{19}F NMR spectrum of **G1⊂1** (376 MHz, CD_3CN, 25 °C).

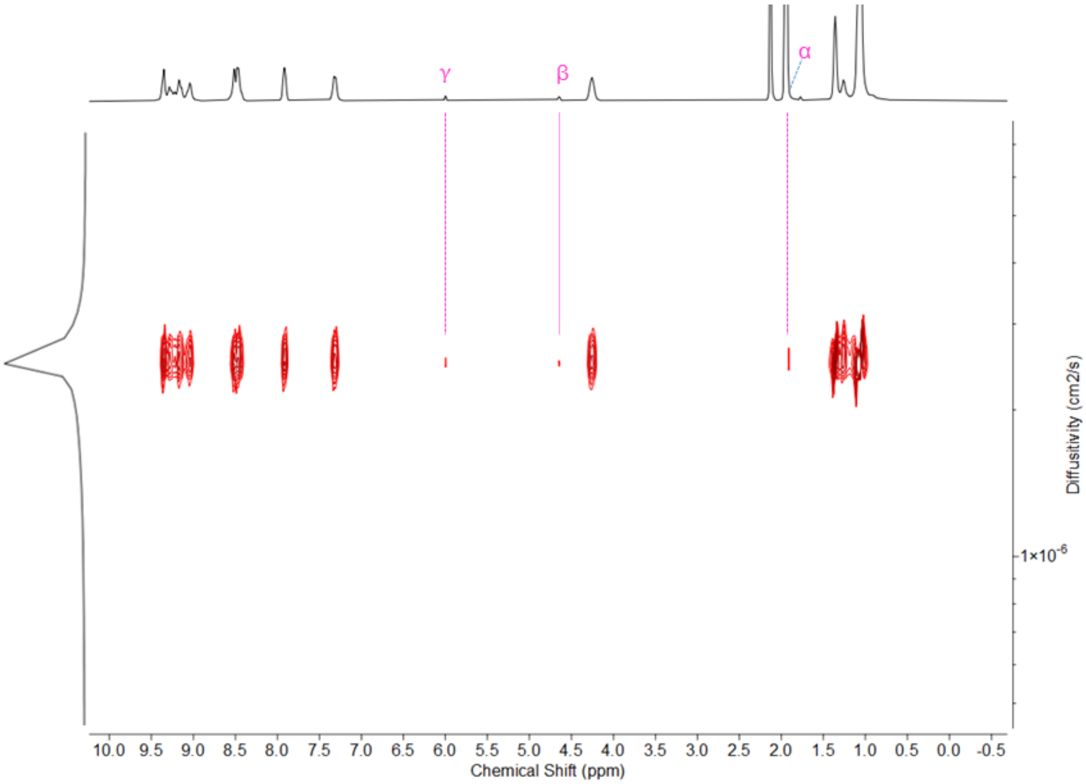

Figure 44. ^{1}H DOSY spectrum of **G1⊂1** (400 MHz, CD_3CN, 25 °C). The diffusion coefficient for both diastereomers in CD_3CN was measured to be 2.50×10^{-6} cm^2/s.

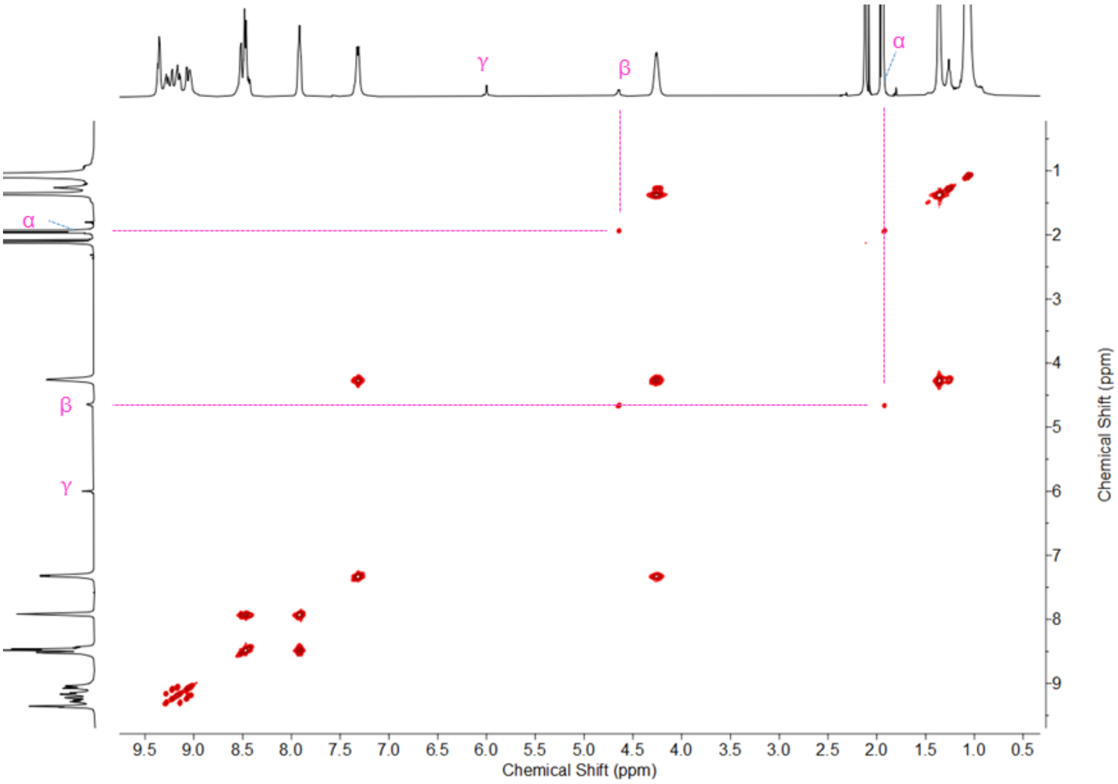

Figure S45. ^{1}H-^{1}H COSY NMR spectrum of **G1⊂1** (500 MHz, CD_3CN, 25 °C).

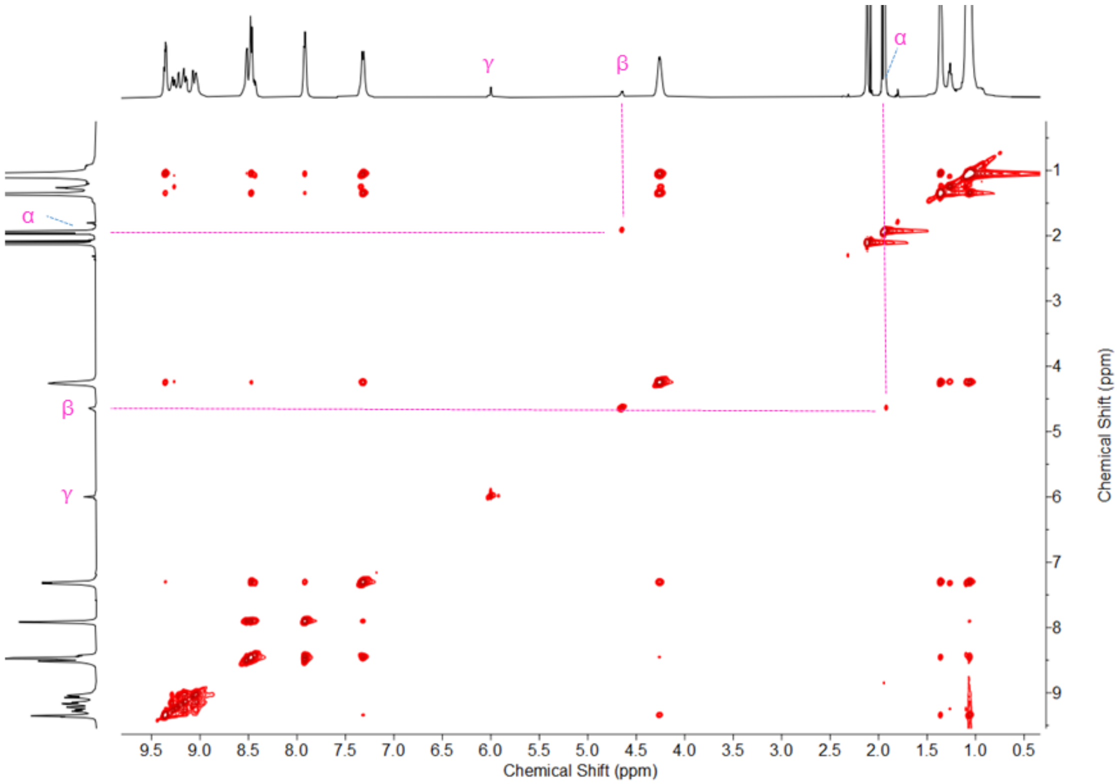

Figure S46. ^{1}H-^{1}H NOESY NMR spectrum of **G1⊂1** (500 MHz, CD_3CN, 25 °C).

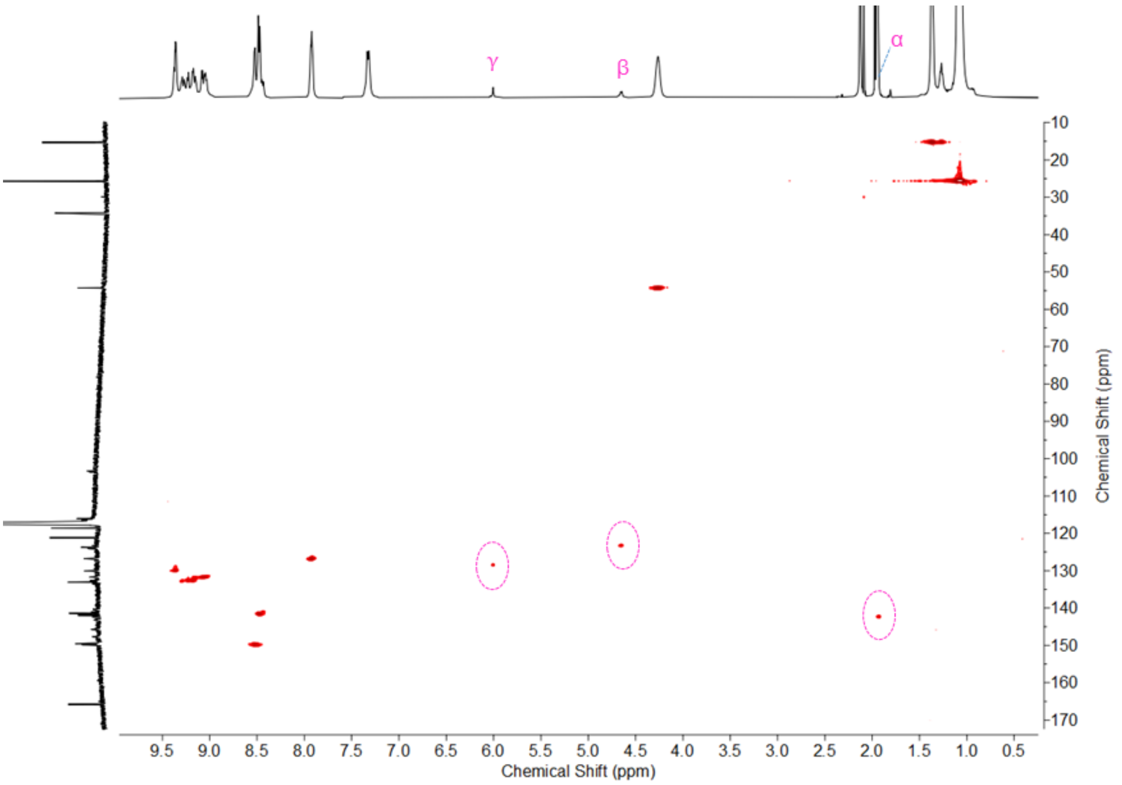

Figure S47. ^{1}H-^{13}C HSQC NMR spectrum of **G1⊂1** (500 MHz, CD_3CN, 25 °C).

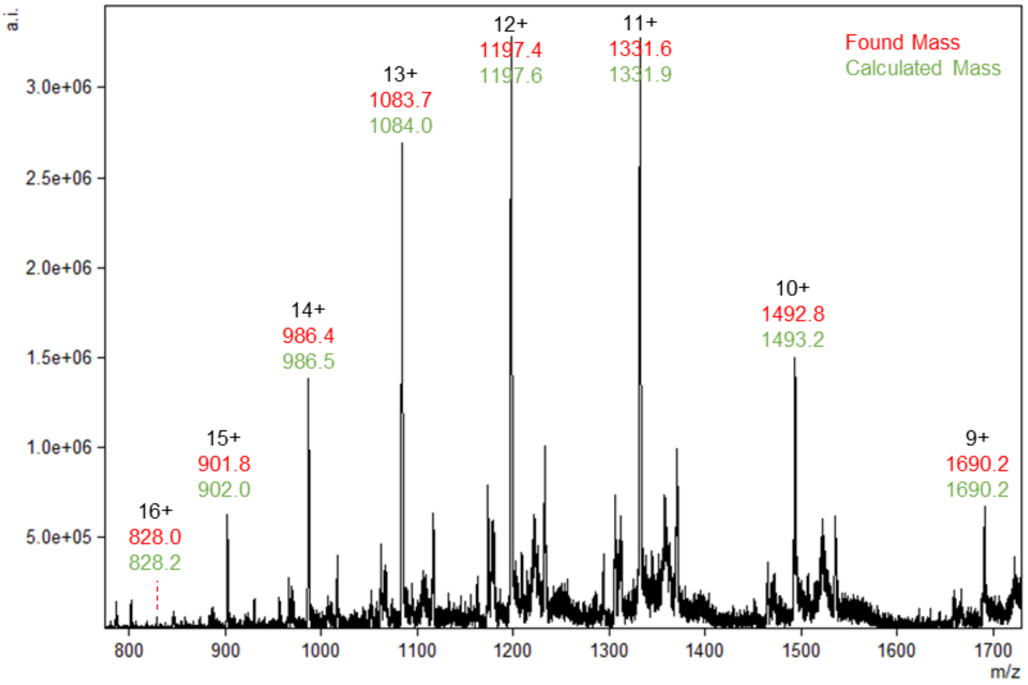

Figure S48. Low-resolution ESI-MS spectrum of **G1⊂1** in MeCN.

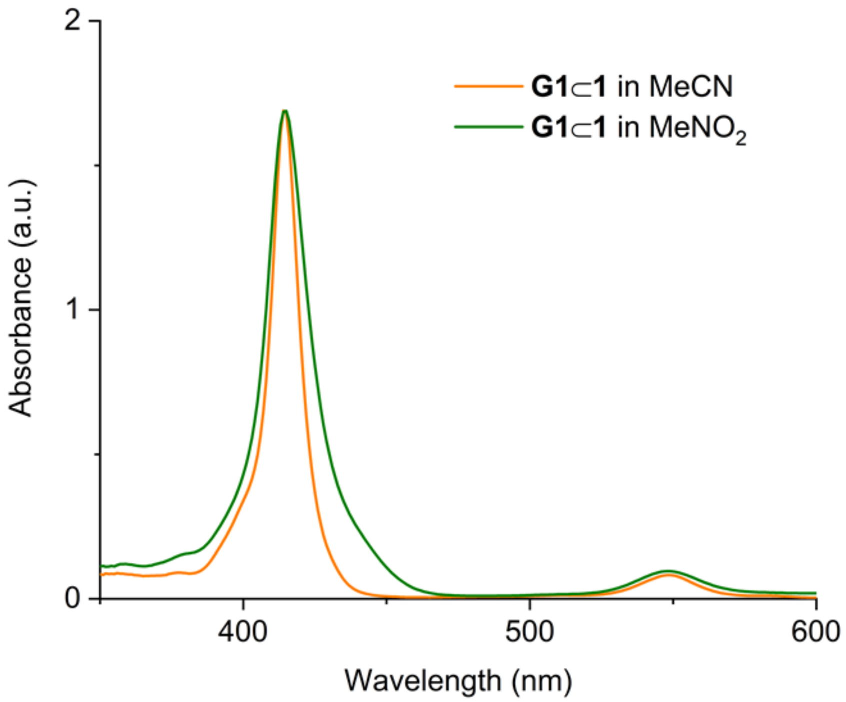

Figure S49. UV/Vis spectra of **G1⊂1** in MeCN and $MeNO_2$.

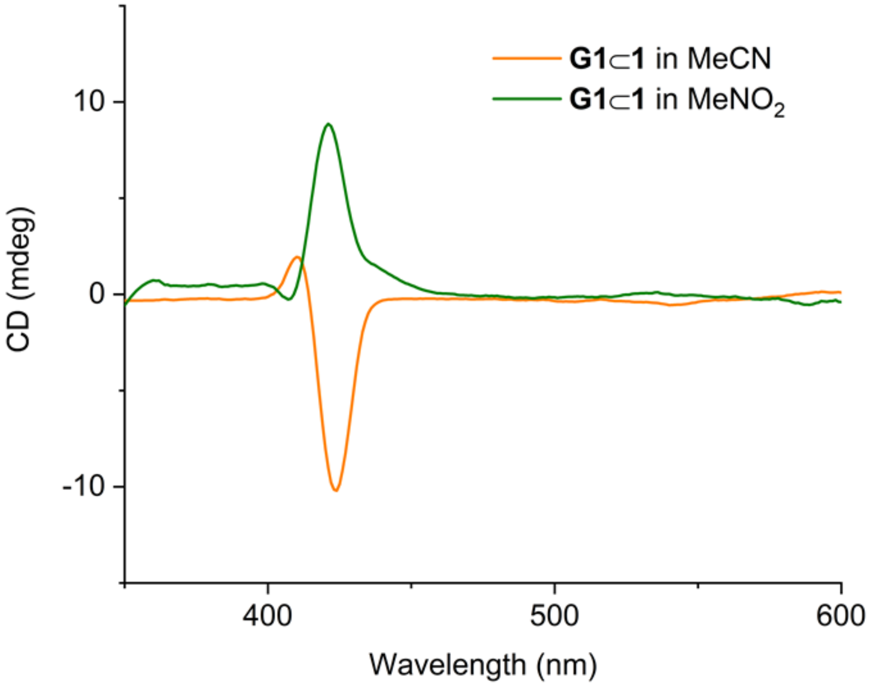

Figure S50. CD spectra of **G1⊂1** in MeCN and $MeNO_2$.

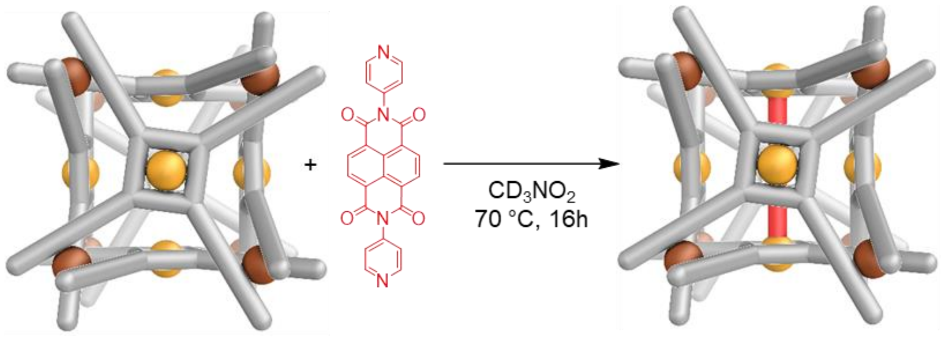

(*S*)$_{24}$-*Λ*$_8$-**1**: (*S*)$_{24}$-*Δ*$_8$-**1**
d.r. = 1:6

G1⊂(*S*)$_{24}$-*Λ*$_8$-**1**:**G1**⊂(*S*)$_{24}$-*Δ*$_8$-**1**
d.r. = 1:3.2

Cage **1** (17.3 mg, 1.0 μmol, 1.0 equiv) and 4,4′-dipyridyl naphthalenediimide (**G1**, 0.42 mg, 1.0 μmol, 1.0 equiv) were combined in $MeNO_2$ (3 mL) in a 15 mL tube. The reaction mixture was stirred at 70 °C for 16 hours. The solvent was reduced to 1 mL, and Et_2O (10 mL) was then added. The precipitate was collected by centrifugation and washed with excess diethyl ether, affording the host-guest complex **G1⊂1** as a purple solid (17.0 mg, 96%).

G1⊂1 consists of a pair of diastereomers. According to the ^{1}H NMR spectrum, the accurate diastereomeric ratio (d.r.) was determined to be 1:3.2. Positive Cotton effects were observed in CD spectrum (Figure S50), which is in line with the observations of parent cage **1** in $MeNO_2$ (Figure S25). The major diastereomer was inferred to be **G1**⊂(*S*)$_{24}$-*Δ*$_8$-**1**, while the minor diastereomer is **G1**⊂(*S*)$_{24}$-*Λ*$_8$-**1**.

^{1}H NMR (500 MHz, CD_3NO_2): δ 0.99–1.20 (m, 216H), 1.26–1.36 (m, 55H, **G1**⊂(*S*)$_{24}$-*Δ*$_8$-**1**), 1.37–1.44 (m, 17H, **G1**⊂(*S*)$_{24}$-*Λ*$_8$-**1**), 2.09–2.14 (4H, overlaid with H_2O), 4.26–4.35 (m, 24H, overlaid with CD_3NO_2), 4.65–4.73 (m, 4H), 5.94–6.02 （m, 4H）, 7.09–7.23 (m, 24H), 7.86–7.99 (m, 24H), 8.46–8.56 (m, 24H), 8.58–8.73 (m, 24H), 8.98–9.28 (m, 48H), 9.35–9.43 (m, 18.3H, **G1**⊂(*S*)$_{24}$-*Δ*$_8$-**1**), 9.45–9.51 (m, 5.7H, **G1**⊂(*S*)$_{24}$-*Λ*$_8$-**1**) ppm. **UV/Vis** ($MeNO_2$): λ_{max} 414 nm (Soret band), λ_{max} 549 nm (Q band). **ESI-MS**: m/z = 902.4 [**G1⊂1**-15(NTf_2)]$^{15+}$, 987.1 [**G1⊂1**-14(NTf_2)]$^{14+}$, 1084.0 [**G1⊂1**-13(NTf_2)]$^{13+}$, 1198.2 [**G1⊂1**-12(NTf_2)]$^{12+}$, 1332.6 [**G1⊂1**-11(NTf_2)]$^{11+}$, 1494.1 [**G1⊂1**-10(NTf_2)]$^{10+}$,1691.0 [**G1⊂1**-9(NTf_2)]$^{9+}$.

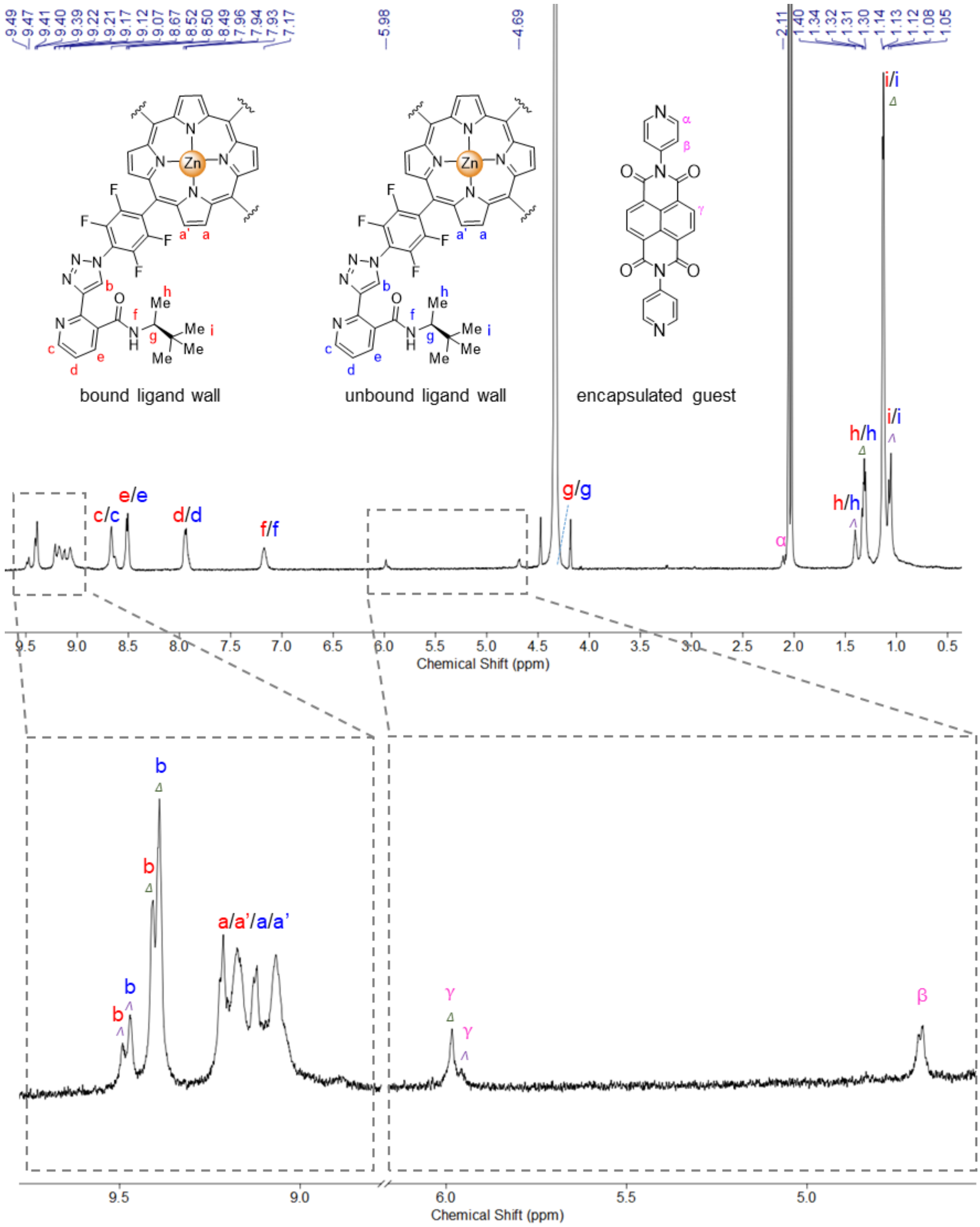

Figure S51. ^1H NMR spectrum of **G1⊂1** (500 MHz, CD_3NO_2, 25 °C).

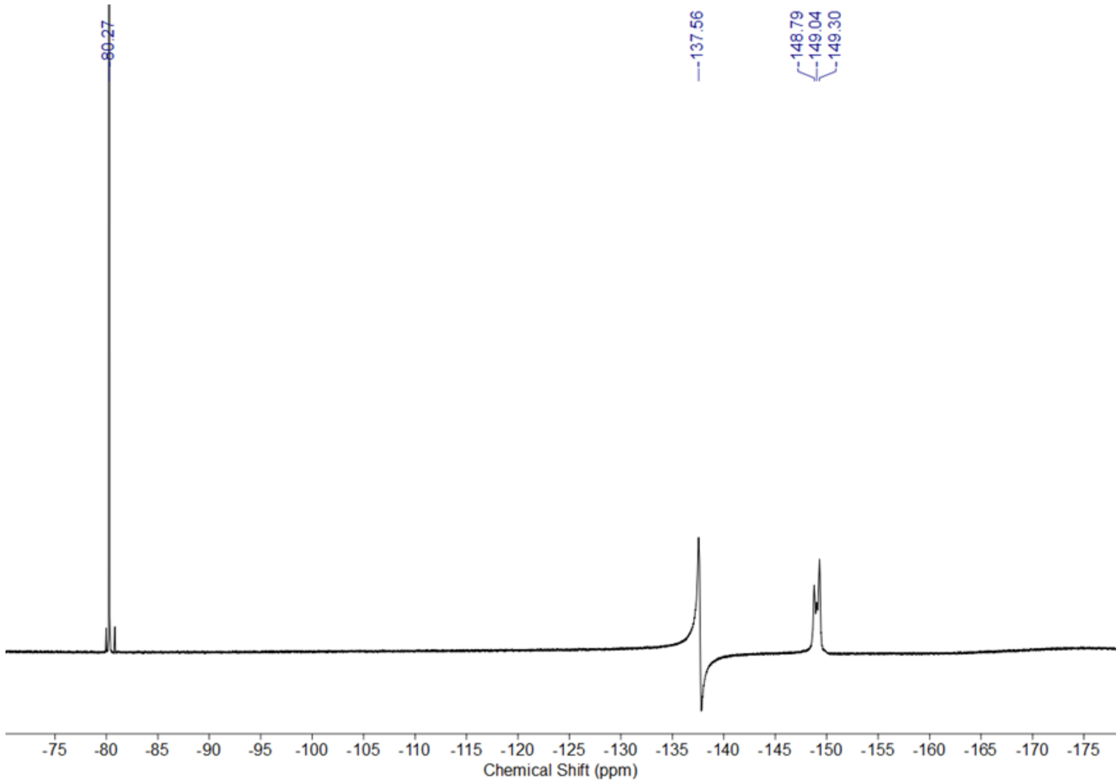

Figure S52. ^{19}F NMR spectrum of **G1⊂1** (376 MHz, CD_3NO_2, 25 °C).

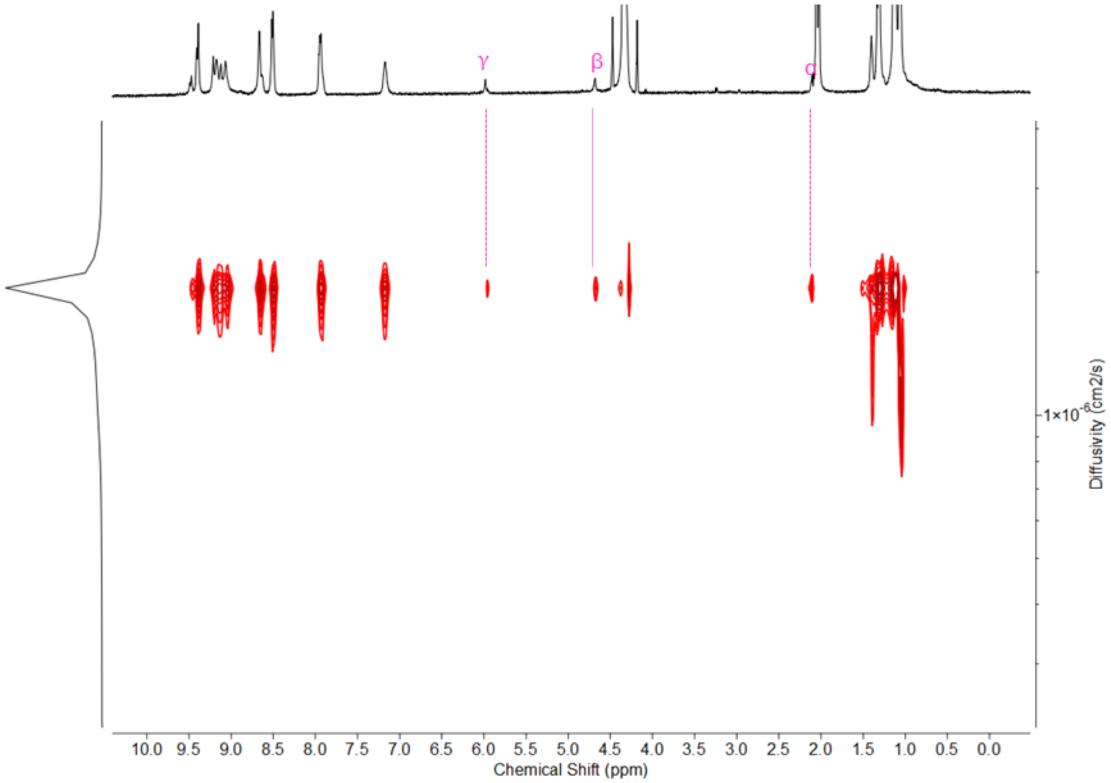

Figure 53. ^{1}H DOSY spectrum of **G1⊂1** (400 MHz, CD_3NO_2, 25 °C). The diffusion coefficient for both diastereomers in CD_3NO_2 was measured to be 1.86×10^{-6} cm^2/s.

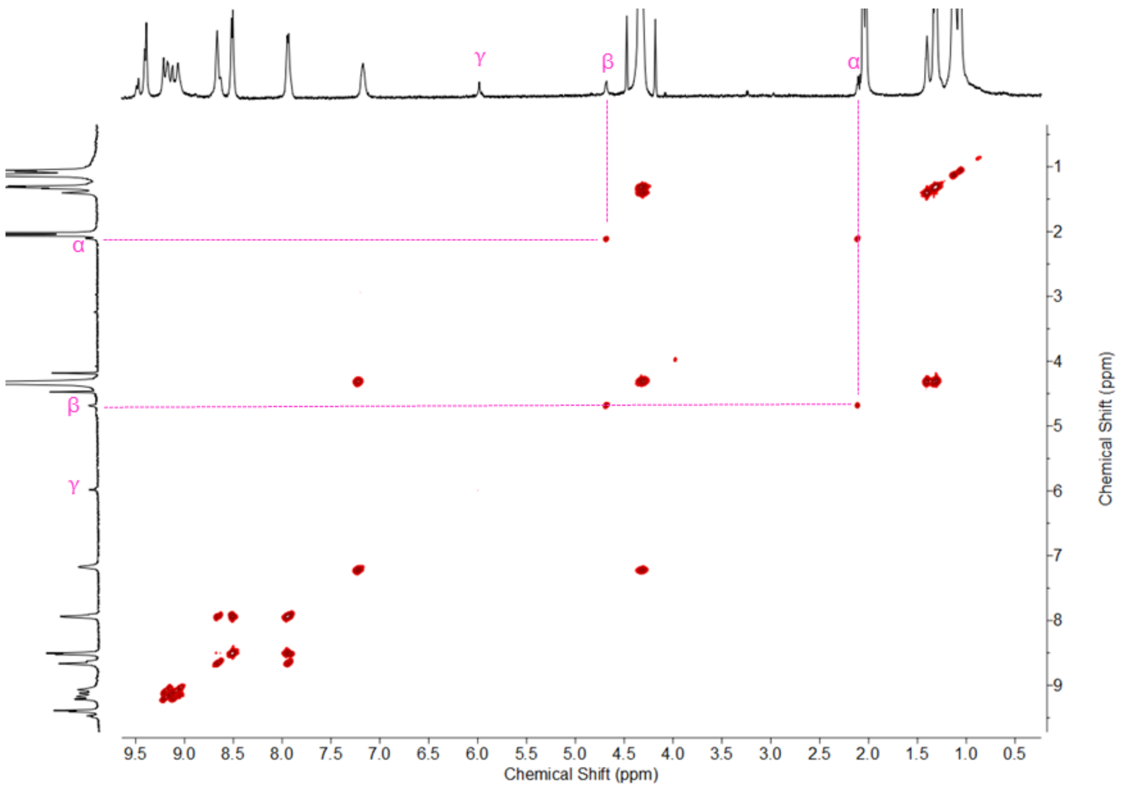

Figure S54. ^{1}H-^{1}H COSY NMR spectrum of **G1⊂1** (500 MHz, CD_3NO_2, 25 °C).

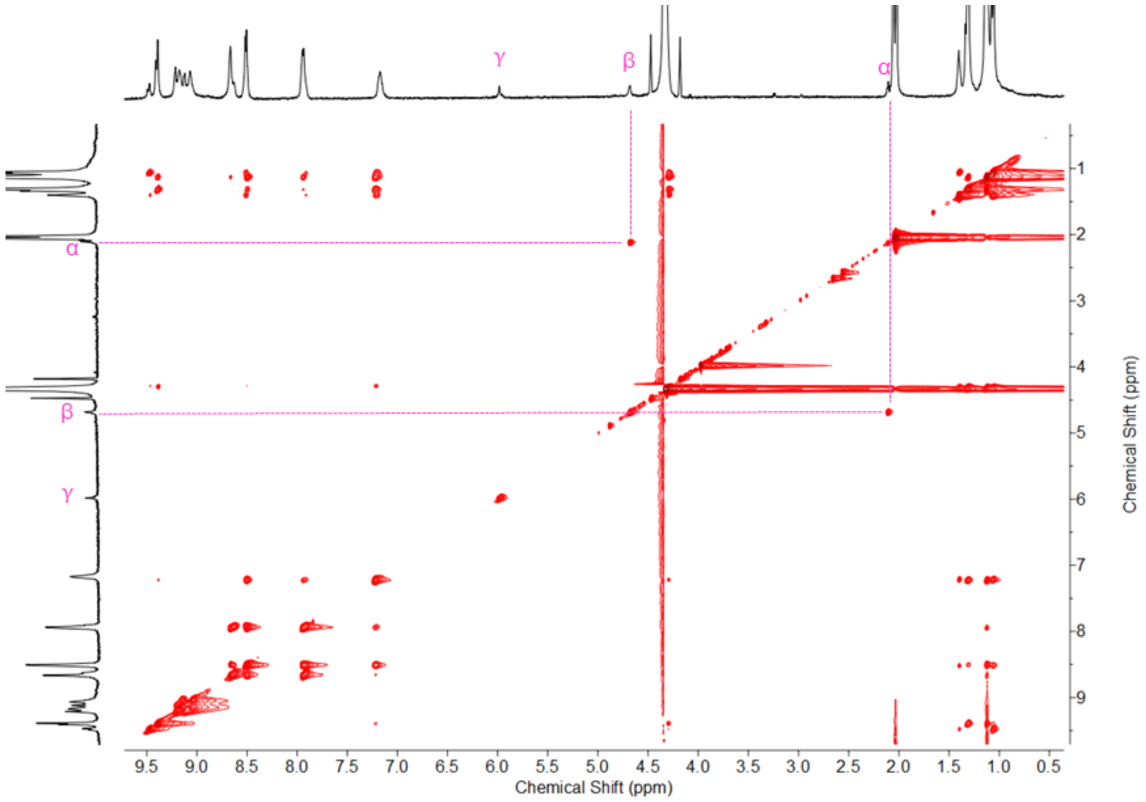

Figure S55. ^{1}H-^{1}H NOESY NMR spectrum of **G1⊂1** (500 MHz, CD_3NO_2, 25 °C).

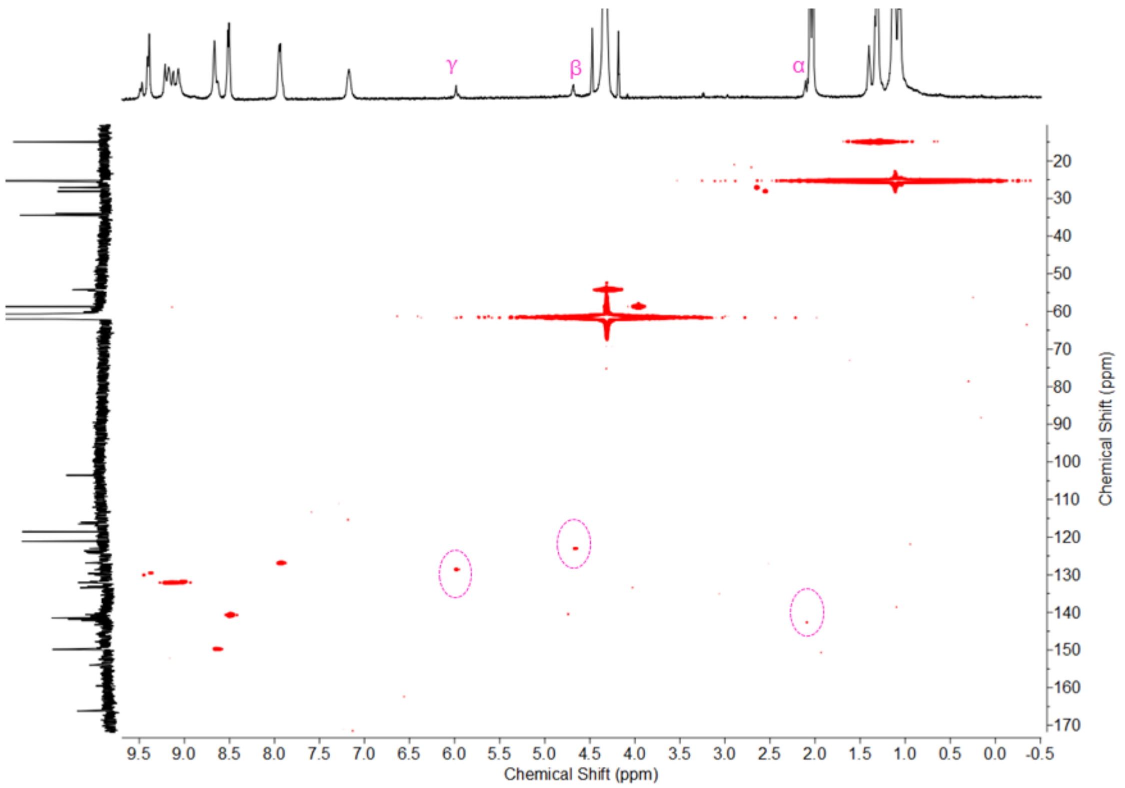

Figure S56. ${}^{1}H$-${}^{13}C$ HSQC NMR spectrum of **G1⊂1** (500 MHz, CD_3NO_2, 25 °C).

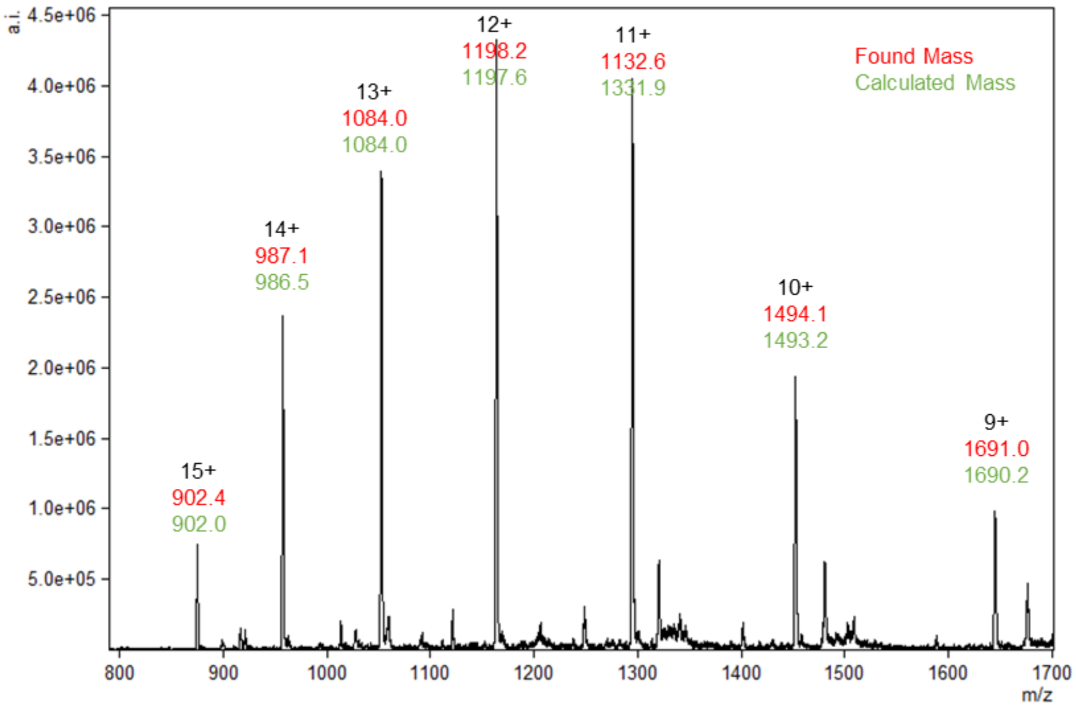

Figure S57. Low-resolution ESI-MS spectrum of **G1⊂1** in $MeNO_2$.

5.2 Encapsulation of Guest 2

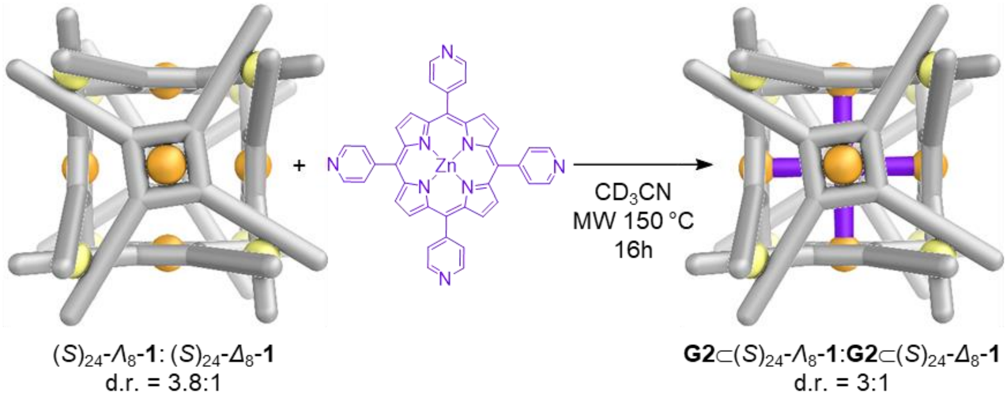

Cage **1** (17.3 mg, 1.0 µmol, 1.0 equiv) and tetrapyridyl Zn-porphyrin (**G2**, 0.75 mg, 1.1 µmol, 1.0 equiv) were combined in MeCN (3 mL) in a sealed tube. The reaction mixture was stirred at 150 °C for 2 hours in the microwave reactor. The suspension was filtered, and the filtrate reduced to 1 mL. Et_2O (10 mL) was then added. The precipitate was collected by centrifugation and washed with excess diethyl ether, affording the host-guest complex **G2**⊂**1** as a purple solid (16.9 mg, 94%).

G2⊂**1** consists of a pair of diastereomers. According to the ^{1}H NMR spectrum, the diastereomeric ratio (d.r.) was determined to be 3:1. Negative Cotton effects were observed in the CD spectrum (Figure S65), which is in line with the observations of parent cage **1** in MeCN (Figure S25). The major diastereomer was inferred to be **G2**⊂$(S)_{24}$-Λ_8-**1**, while the minor diastereomer is **G2**⊂$(S)_{24}$-Δ_8-**1**.

^{1}H NMR (500 MHz, CD_3CN): δ 0.94–1.15 (m, 216H), 1.24–1.43 (m, 72H), 2.16–2.20 (m, 8H, overlaid with H_2O), 4.08–4.39 (m, 24H), 5.33–5.38 (m, 8H), 6.16 (s, 6H, **G2**⊂$(S)_{24}$-Λ_8-**1**), 6.18 (s, 2H, **G2**⊂$(S)_{24}$-Δ_8-**1**), 7.17–7.40 (m, 24H), 7.82–7.98 (m, 24H), 8.34–8.61 (m, 48H), 8.71–9.50 (m, 72H) ppm. **UV/Vis** (MeCN): λ_{max} 414 nm (Soret band), λ_{max} 551 nm (Q band). **ESI-MS**: m/z = 919.2 [**G2**⊂**1**-15(NTf_2)]$^{15+}$, 1005.0 [**G2**⊂**1**-14(NTf_2)]$^{14+}$, 1104.0 [**G2**⊂**1**-13(NTf_2)]$^{13+}$, 1219.3 [**G2**⊂**1**-12(NTf_2)]$^{12+}$, 1355.6 [**G1**⊂**2**-11(NTf_2)]$^{11+}$, 1519.2 [**G2**⊂**1**-10(NTf_2)]$^{10+}$, 1719.4 [**G2**⊂**1**-9(NTf_2)]$^{9+}$.

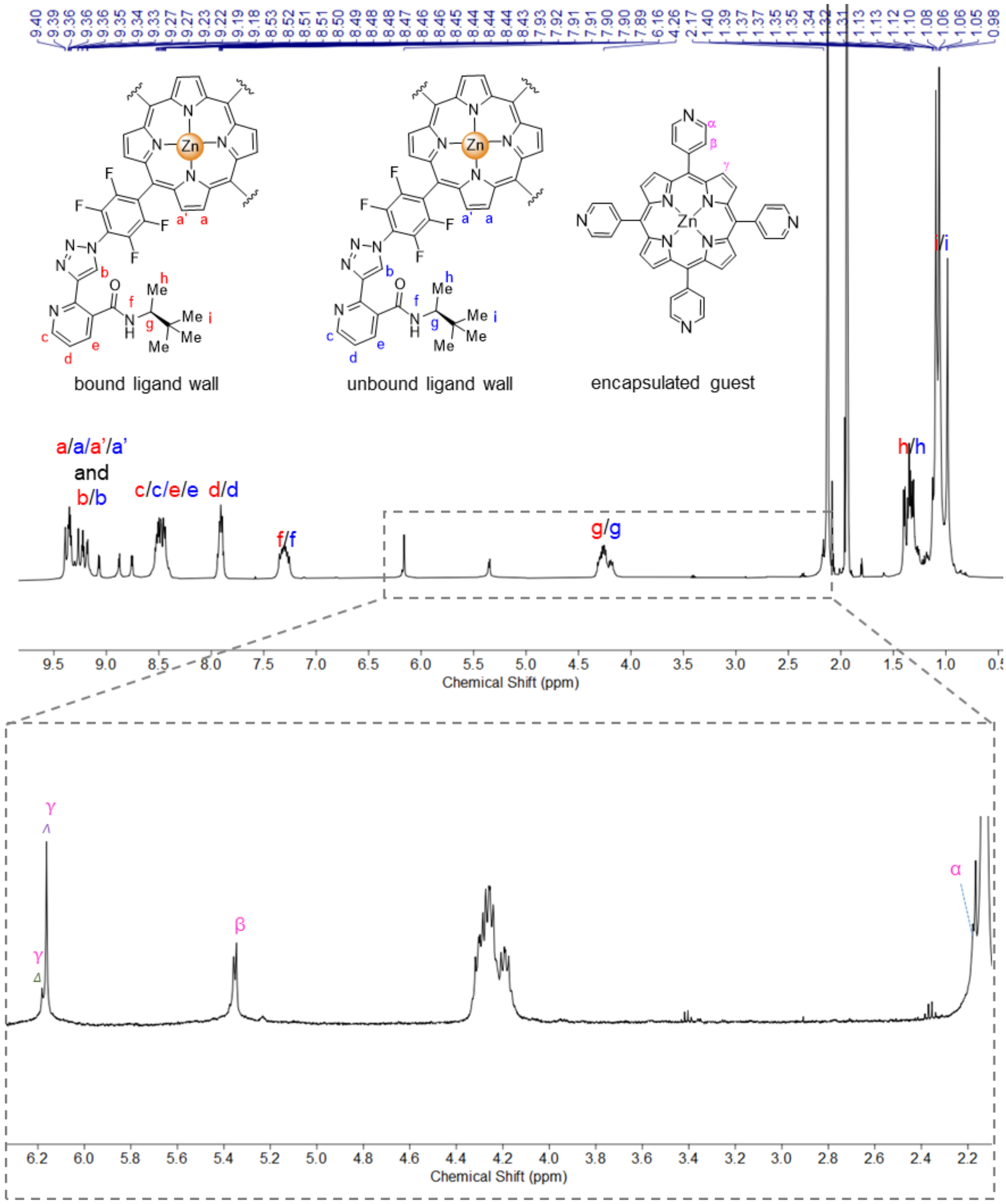

Figure S58. ^{1}H NMR spectrum of **G2⊂1** (500 MHz, CD_3CN, 25 °C).

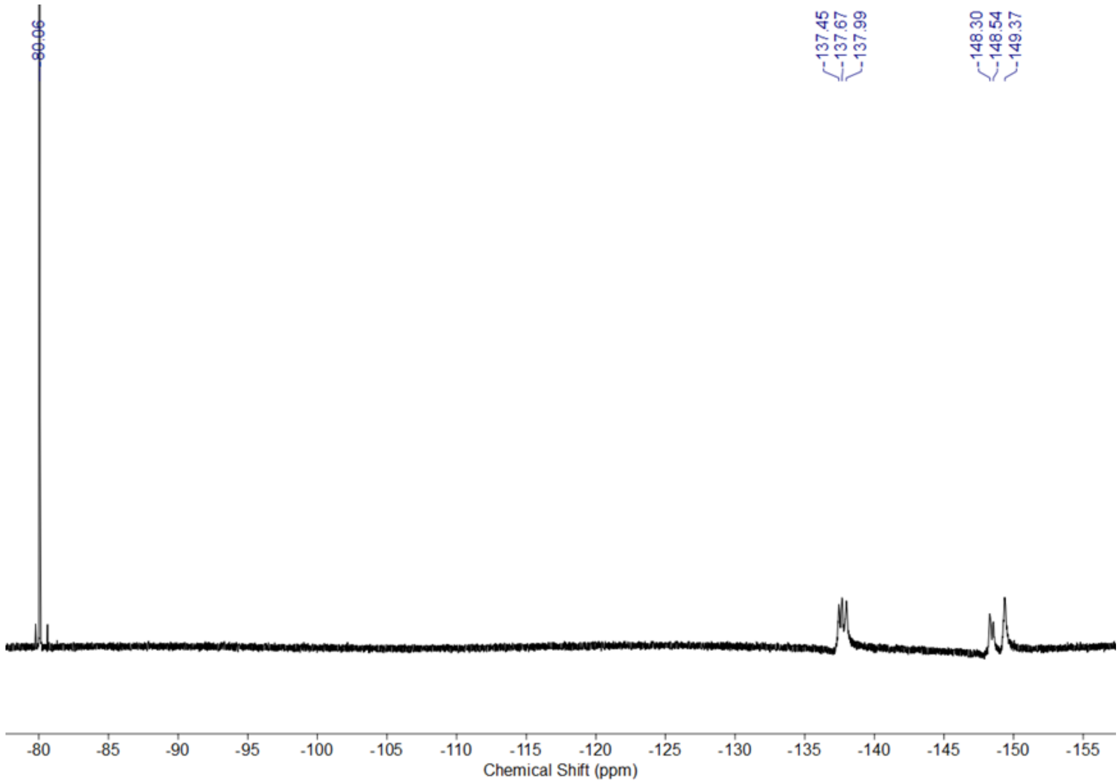

Figure S59. ^{19}F NMR spectrum of **G2⊂1** (376 MHz, CD_3CN, 25 °C).

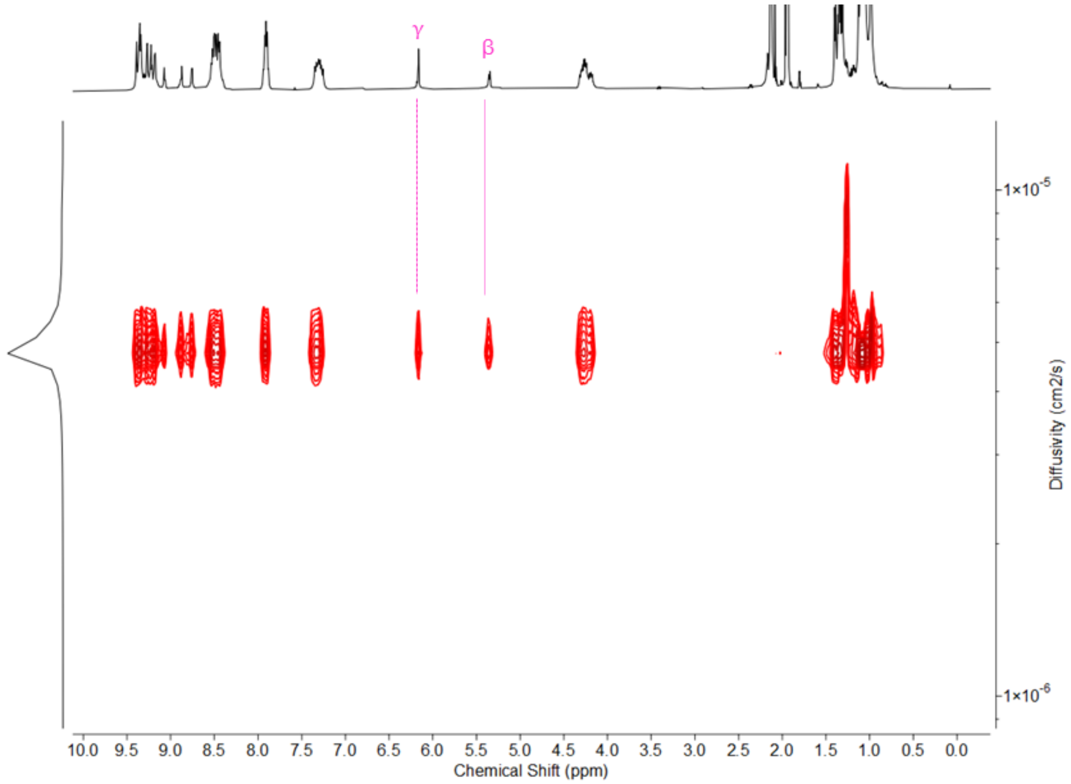

Figure 60. ^{1}H DOSY spectrum of **G2⊂1** (400 MHz, CD_3CN, 25 °C). The diffusion coefficient for both diastereomers in CD_3CN was measured to be 4.85×10^{-6} cm^2/s.

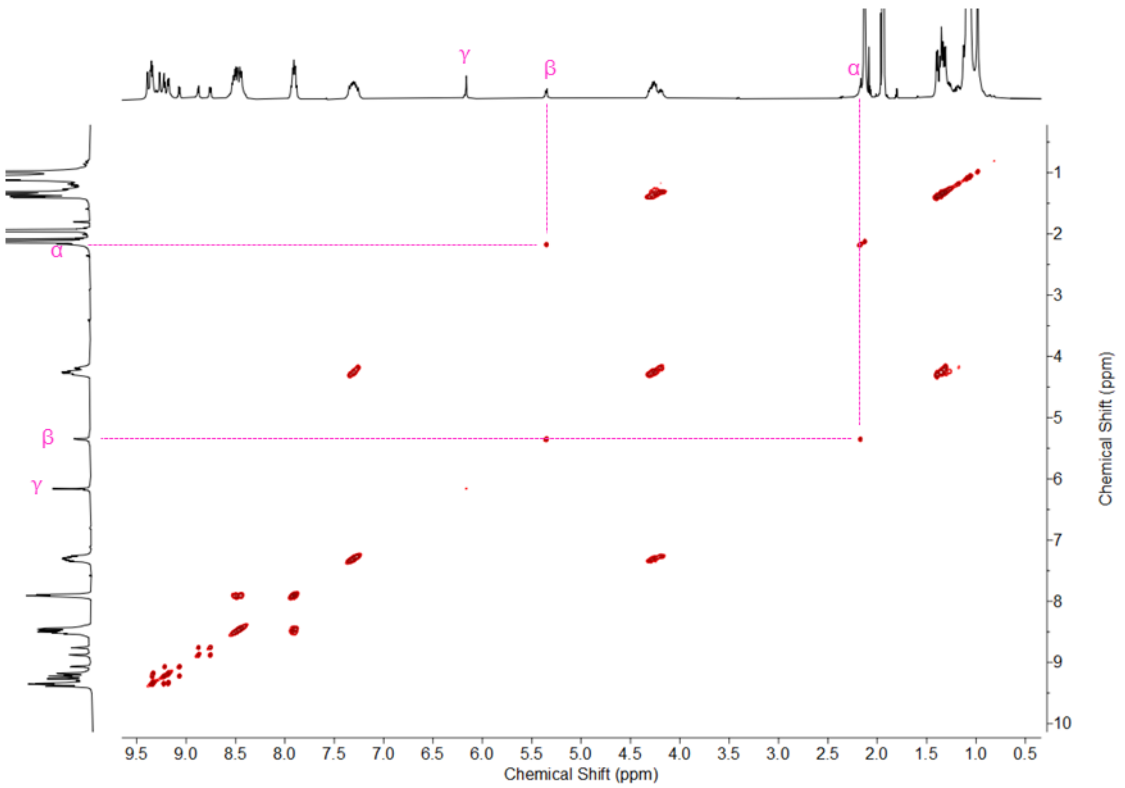

Figure S61. ^{1}H-^{1}H COSY NMR spectrum of **G2⊂1** (500 MHz, CD_3CN, 25 °C).

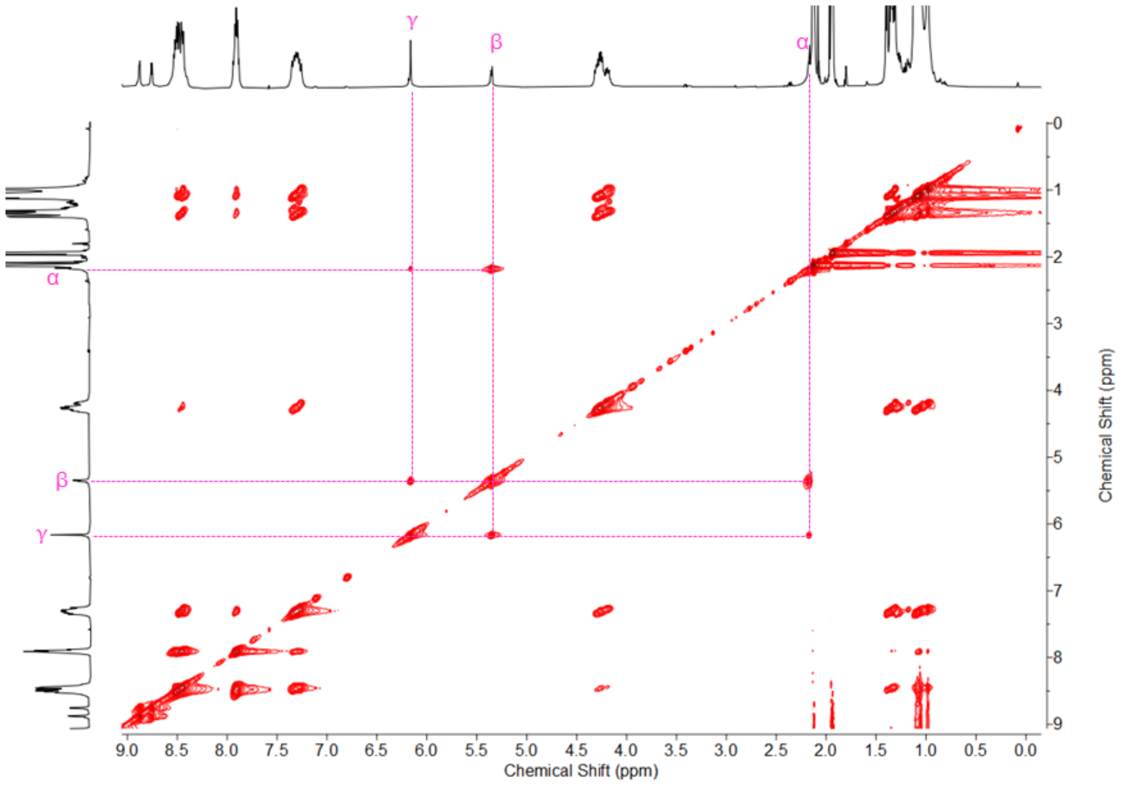

Figure S62. ^{1}H-^{1}H NOESY NMR spectrum of **G2⊂1** (500 MHz, CD_3CN, 25 °C).

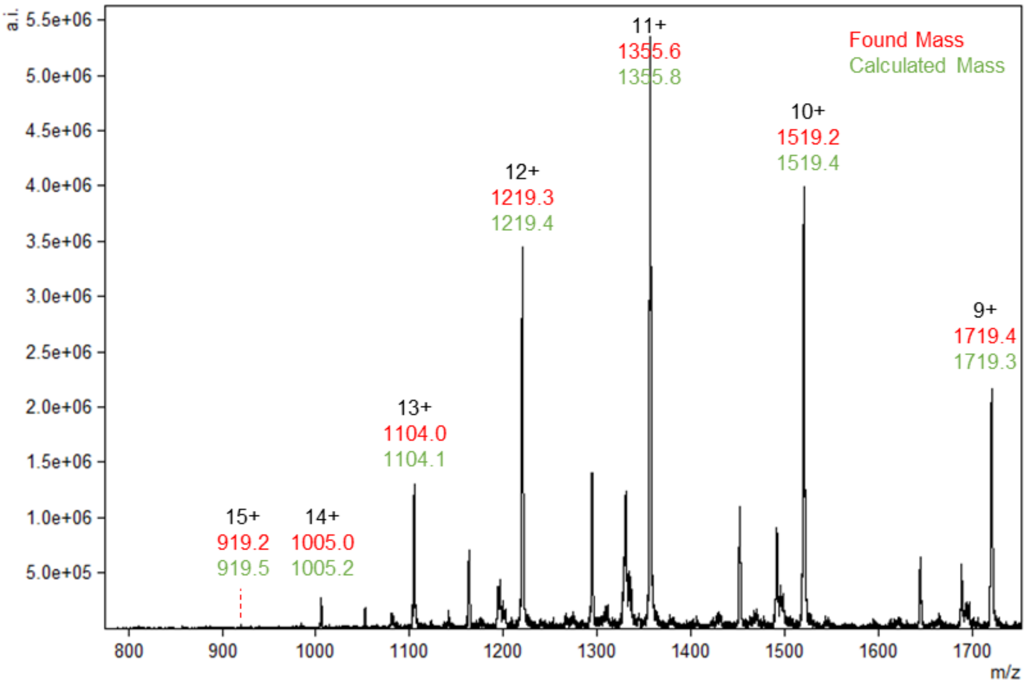

Figure S63. Low-resolution ESI-MS spectrum of **G2**⊂**1** in MeCN.

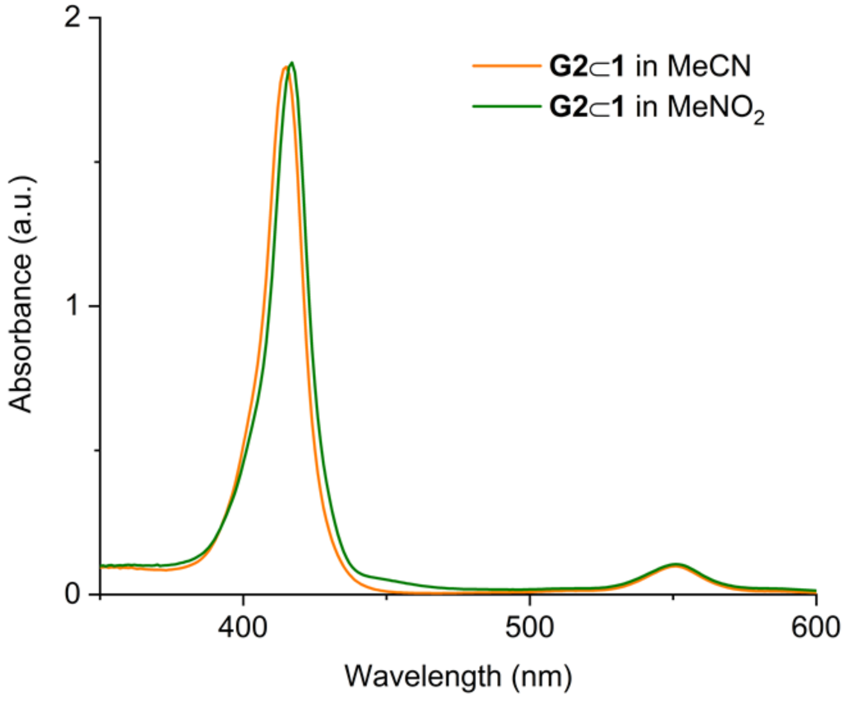

Figure S64. UV/Vis spectra of **G2**⊂**1** in MeCN and $MeNO_2$.

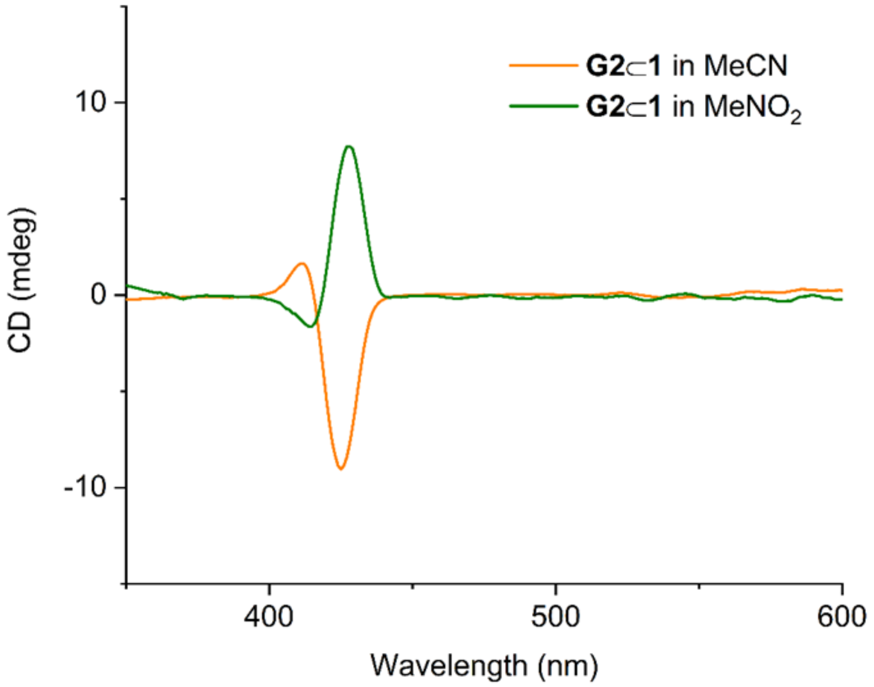

Figure S65. CD spectra of **G2⊂1** in MeCN and $MeNO_2$.

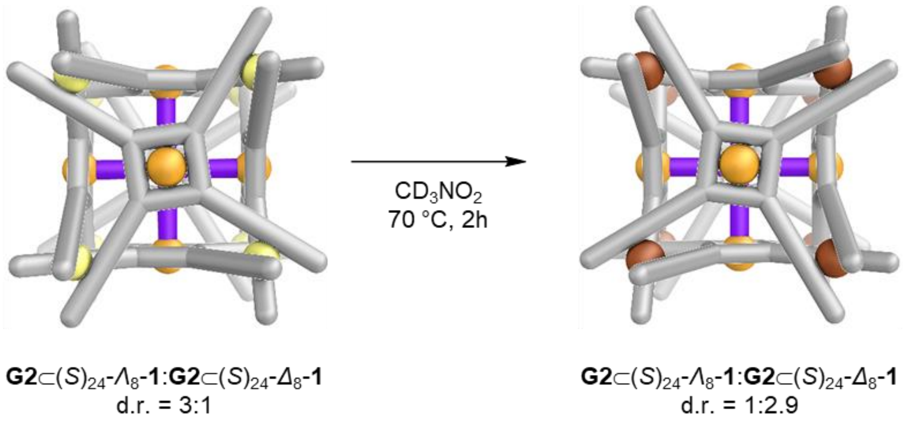

Cage **1** was not able to encapsulate tetrapyridyl Zn-porphyrin (**G2**) in nitromethane, due to the insolubility of **G2** in nitromethane. Inspired by the solvent-dependent diastereocontrol for this dynamic system, we attempted to dissolve the solid-state **G2⊂1** in nitromethane. As expected, a reverse diastereoselectivity was observed. According to the ^{1}H NMR spectrum, the diastereomeric ratio (d.r.) was determined to be 3:1. Positive Cotton effects were observed in CD spectrum (Figure S65), which is in line with the observations of parent cage **1** in $MeNO_2$ (Figure S25). The major

diastereomer was inferred to be **G2**⊂(*S*)$_{24}$-*Δ*$_8$-**1**, while the minor diastereomer is **G2**⊂(*S*)$_{24}$-*Λ*$_8$-**1**.

^{1}**H NMR** (500 MHz, CD_3CN): δ 0.80–1.54 (m, 288H), 2.32 (s, 8H), 4.21–4.40 (m, 24H, overlaid with CD_3NO_2), 5.22 (s, 2H, **G2**⊂(*S*)$_{24}$-*Λ*$_8$-**1**), 5.41 (s, 6H, **G2**⊂(*S*)$_{24}$-*Δ*$_8$-**1**), 6.19 (s, 8H), 7.84–8.01 (m, 24H), 8.42–8.58 (m, 24H), 8.60–8.73 (m, 24H), 8.74–9.56 (m, 72H) ppm. **UV/Vis** (MeCN): $λ_{max}$ 417 nm (Soret band), $λ_{max}$ 551 nm (Q band).

Note: Activated H_f (NH) is not shown in CD_3NO_2 due to H/D exchange.

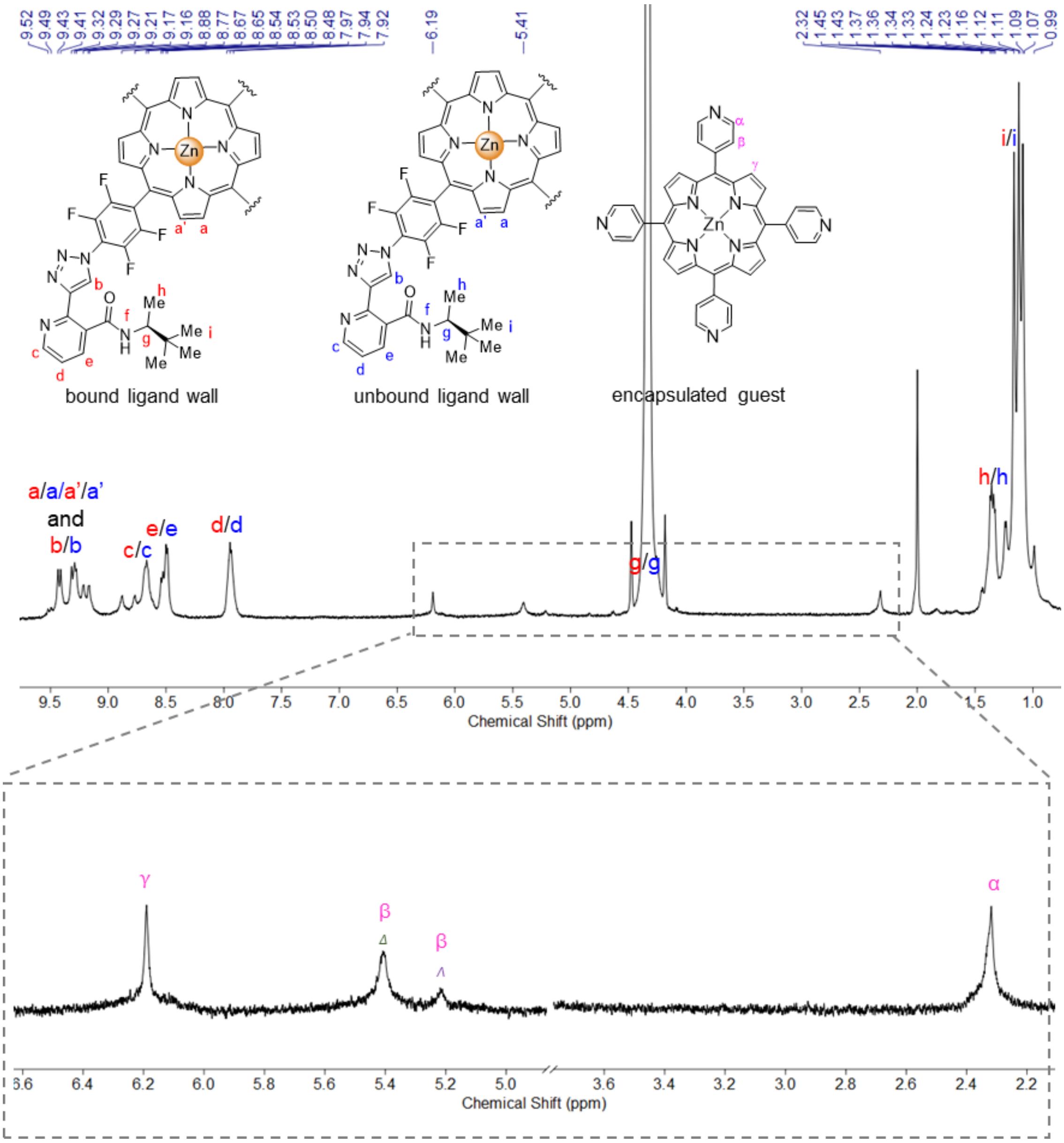

Figure S66. ^{1}H NMR spectrum of **G2**⊂**1** (500 MHz, CD_3NO_2, 25 °C).

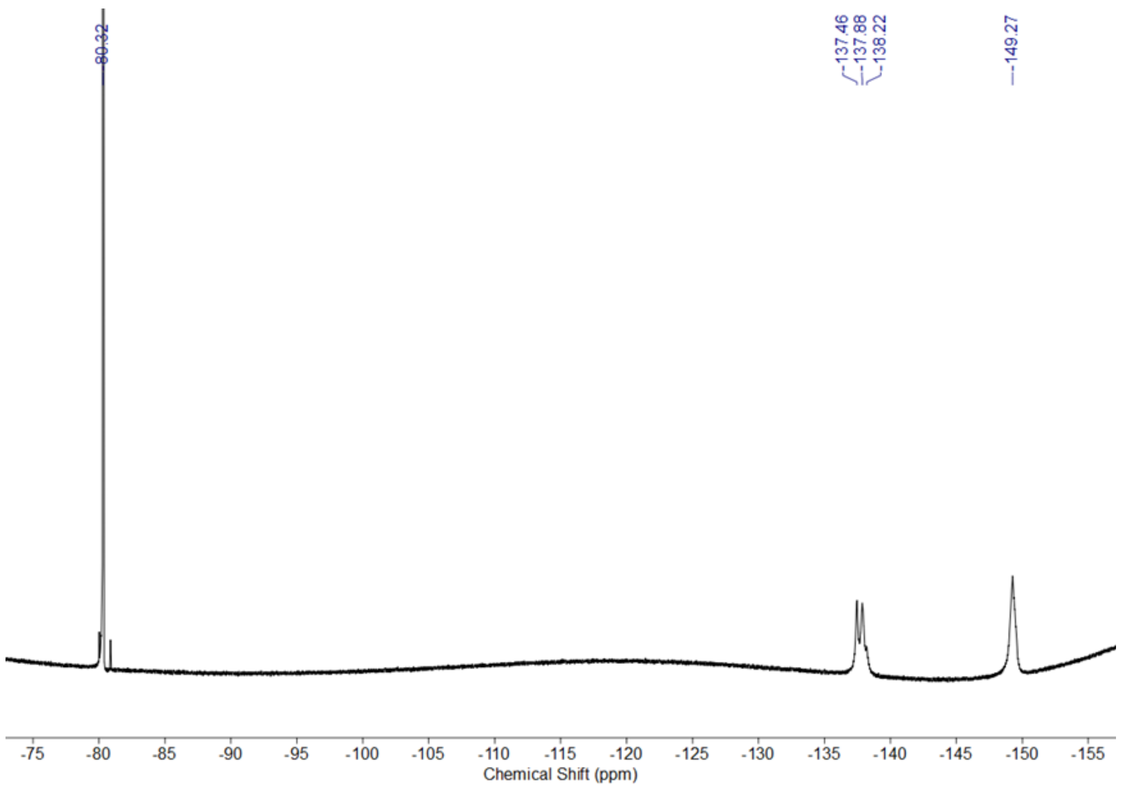

Figure S67. ^{19}F NMR spectrum of **G2⊂1** (376 MHz, CD_3NO_2, 25 °C).

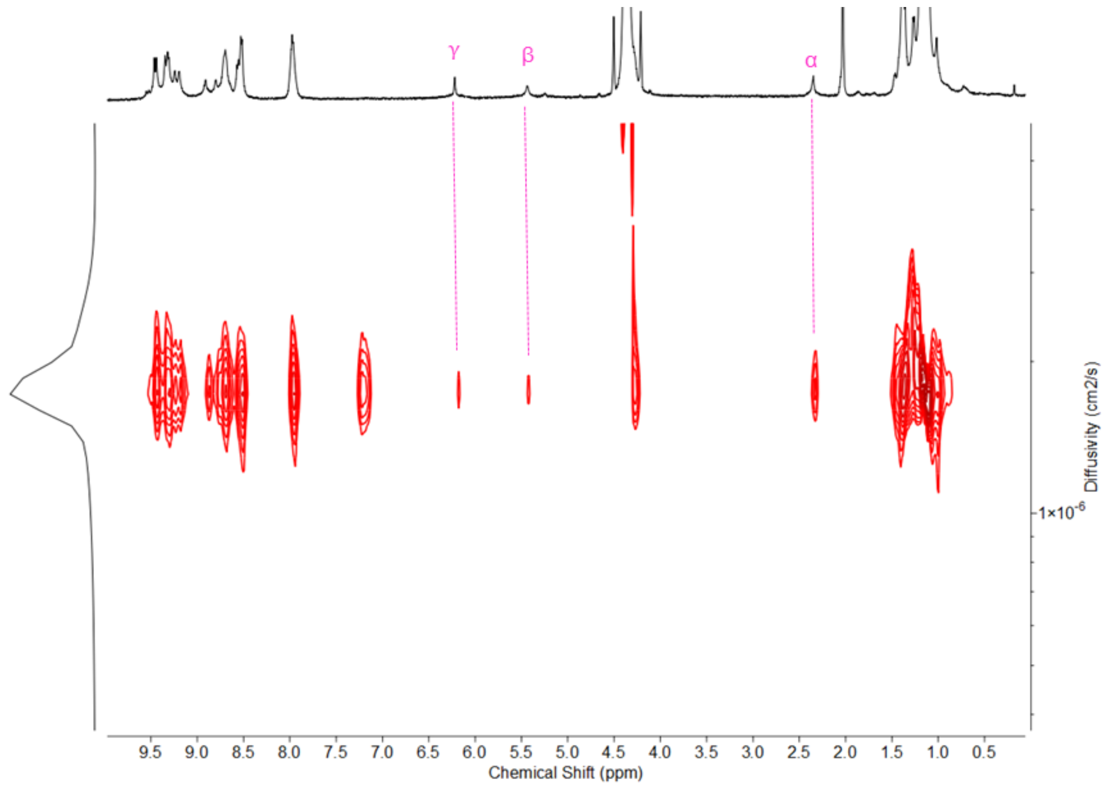

Figure 68. ^{1}H DOSY spectrum of **G2⊂1** (400 MHz, CD_3NO_2, 25 °C). The diffusion coefficient for both diastereomers in CD_3CN was measured to be 1.76×10^{-6} cm^2/s.

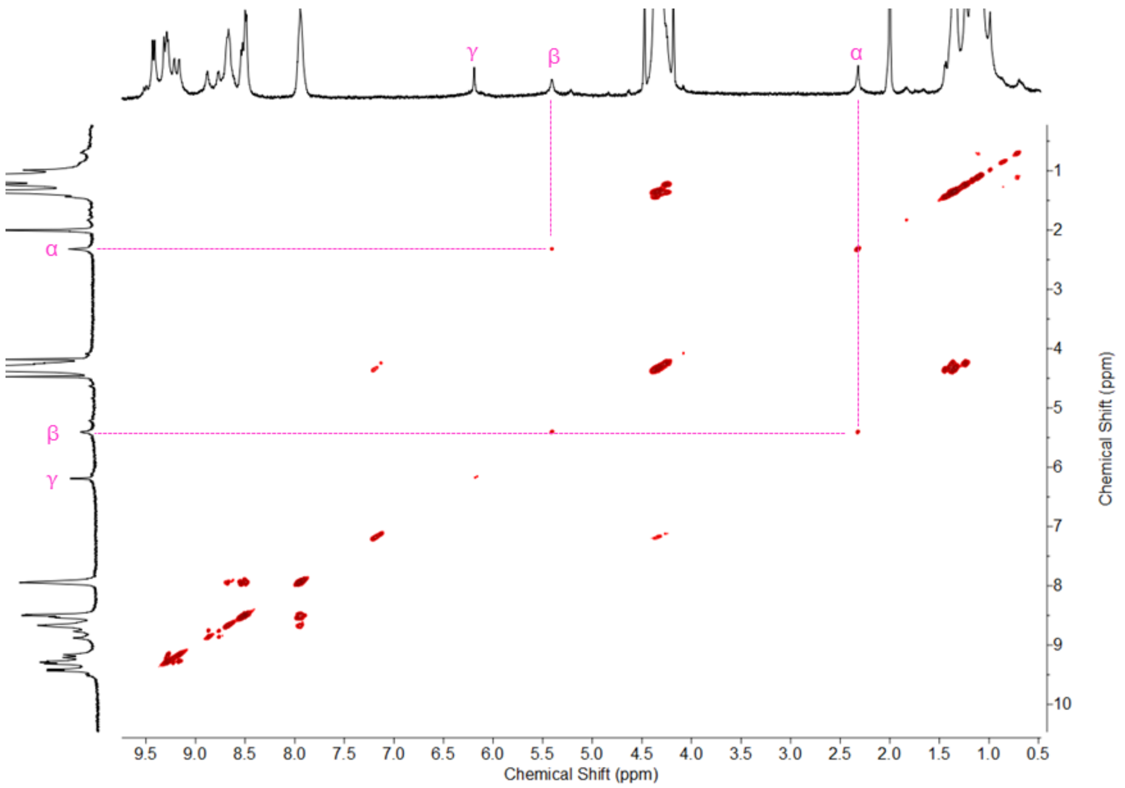

Figure S69. ^{1}H-^{1}H COSY NMR spectrum of **G2⊂1** (500 MHz, CD_3NO_2, 25 °C).

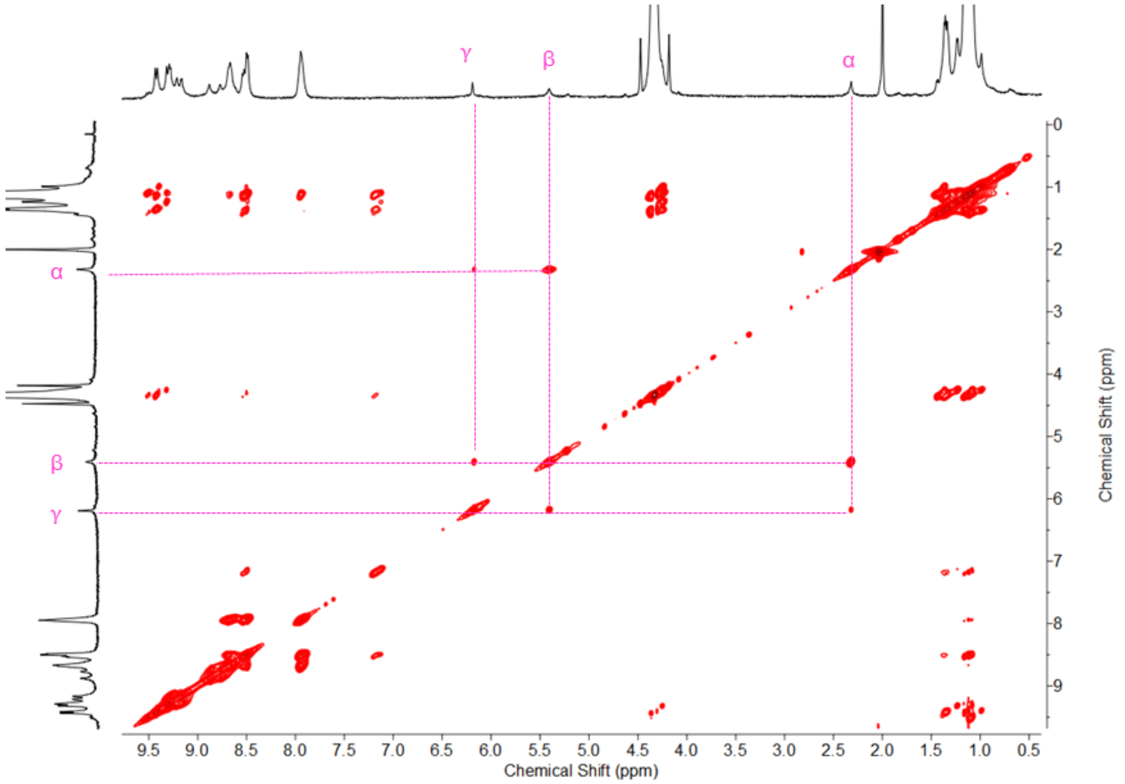

Figure S70. ^{1}H-^{1}H NOESY NMR spectrum of **G2⊂1** (500 MHz, CD_3NO_2, 25 °C).

5.3 Binding 4,4'-Bipyridine by G2⊂1

Upon addition of 4,4'-bipyridine into **G2⊂1**, it was found that 4,4'-bipyridine was bound by complex **G2⊂1** at 25 °C (Figure 71). NMR peaks of guest became broad. Clear chemical shifts for H_β and H_γ of bound **G2** and $H_{a/a'}$ of ligand walls were observed. The shifts of H_β and H_γ indicated that the binding process is within complex **G2⊂1**. However, cage **1** was not able to bind 4,4'-bipyridine with no changes in chemical shifts for the host (Figure 72).

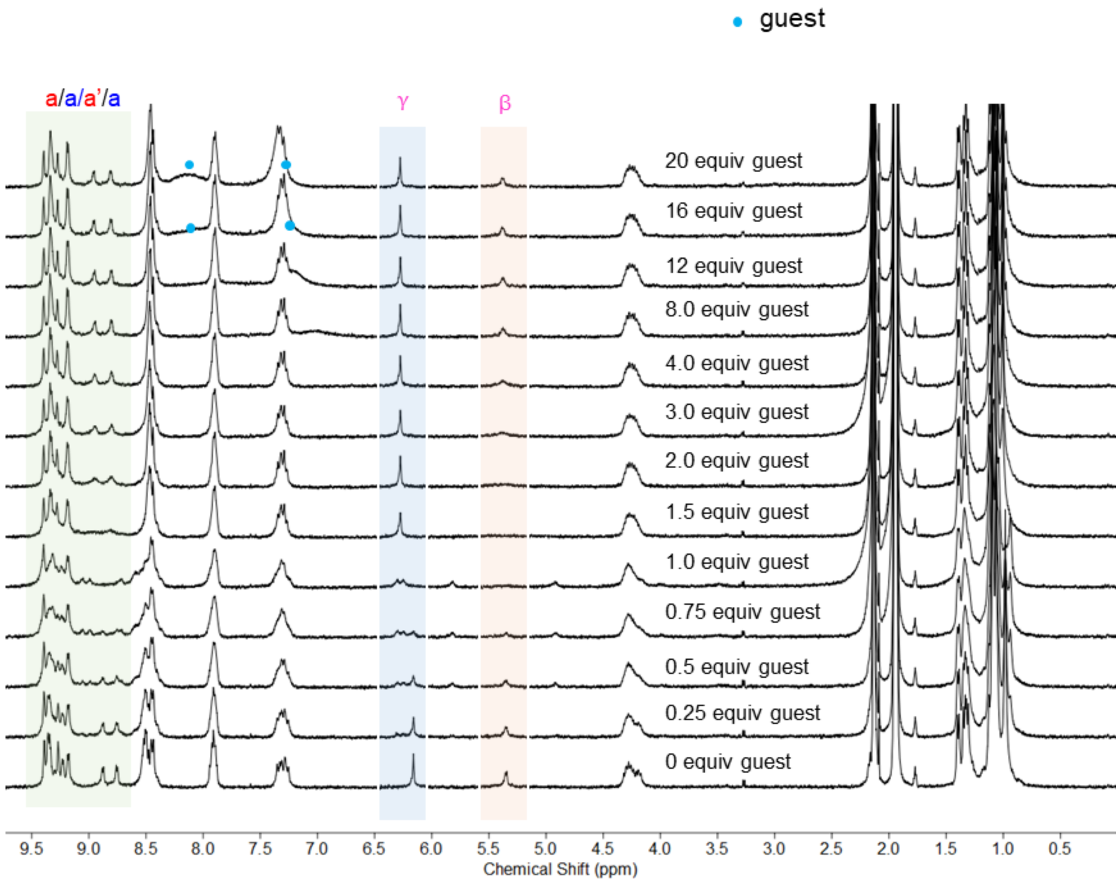

Figure 71. ^{1}H NMR spectra of **G2⊂1** (2 mM) upon addition of 4,4'-bipyridine (400 MHz, CD_3CN, 25 °C). Chemical shifts for H_β and H_γ of bound **G2** and $H_{a/a'}$ of ligand walls are highlighted.

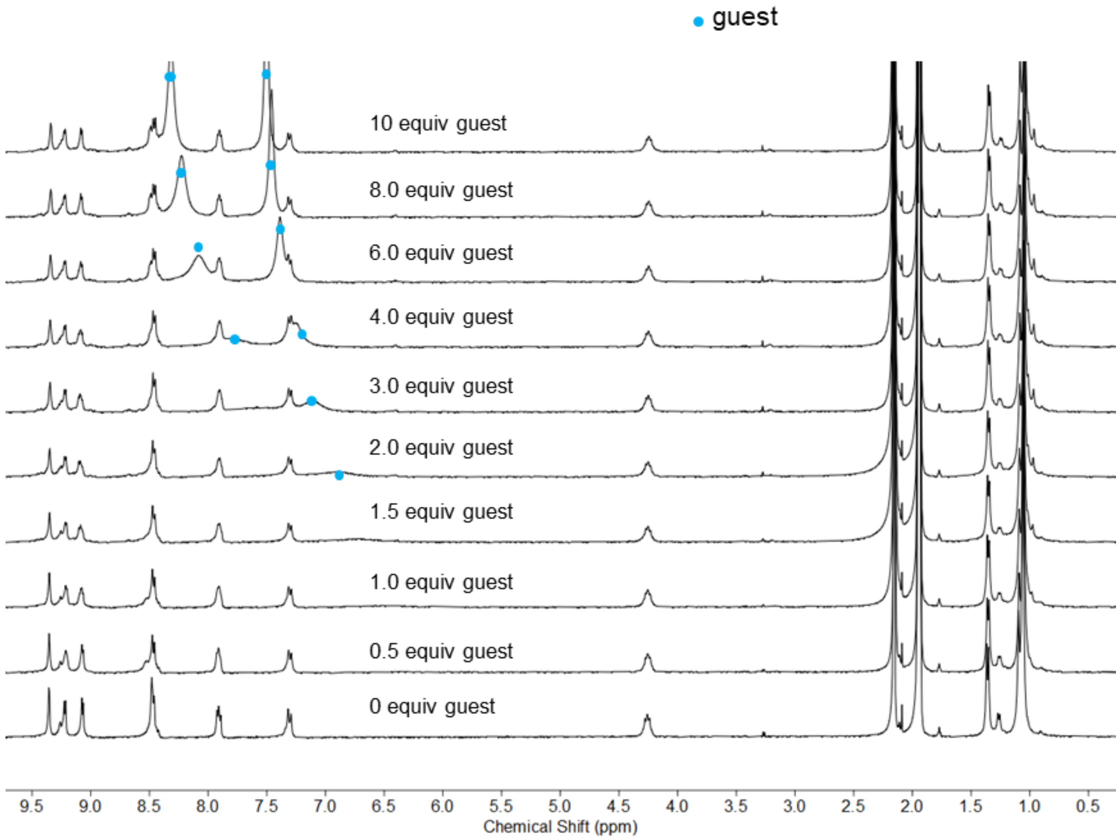

Figure 72. ^{1}H NMR spectra of cage **1** (2 mM) upon addition of 4,4'-bipyridine (400 MHz, CD_3CN, 25 °C). No changes in chemical shifts for cage **1** were observed.

5.4 Non-Binding Guests

Prospective guests (1.0 µmol, 5 equiv) and cage **1** (3.46mg, 0.2 µmol, 1.0 equiv) were combined in CD_3CN (0.5 mL) in NMR tubes. The reaction mixtures were heated at 70 °C for 16 hours. After cooling down to room temperature, ^1H NMR and ^{19}F NMR spectra were measured within 4 hours. No changes in chemical shift were observed for the following guests.

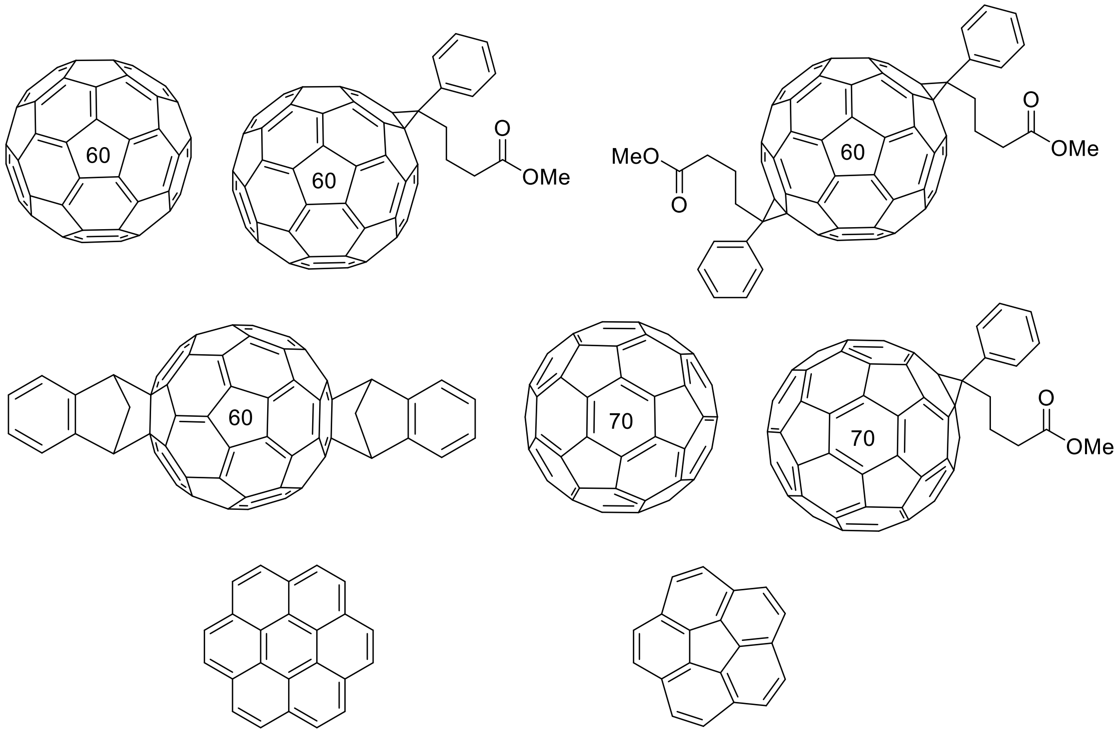

Figure S73. Non-binding Guests.

6 Volume Calculations

In order to determine the available void spaces within the structures of $(S)_{24}$-Λ_8-**1** and $(S)_{24}$-Λ_8-**2**, Molovol[3] calculations based on the crystal structures obtained in this study were performed with anions, disorder and solvent molecules removed. A probe with a radius of 2.4 Å was employed in all cases. The standard parameters are tabulated below, and the results are shown in Figure 74.

Probe mode: one probe

Probe radius: 2.4 Å

Grid resolution: 0.1 Å

Optimization depth: 4

Slightly different results were obtained by VOIDOO calculations using the same probe radius (2.4 Å).[4]

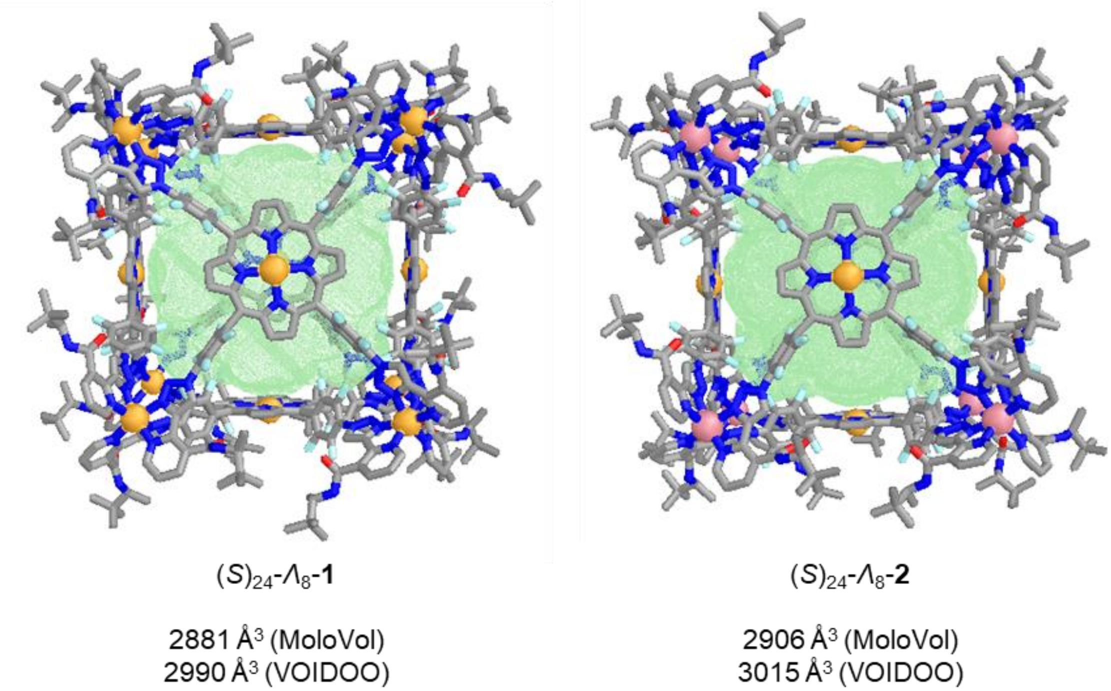

$(S)_{24}$-Λ_8-**1**

2881 Å^3 (MoloVol)
2990 Å^3 (VOIDOO)

$(S)_{24}$-Λ_8-**2**

2906 Å^3 (MoloVol)
3015 Å^3 (VOIDOO)

Figure 74. MoloVol-calculated void space (green mesh) within the crystal structures of $(S)_{24}$-Λ_8-**1** and $(S)_{24}$-Λ_8-**2**.

7 X-ray Crystallography

Data were collected at Beamline I19 of Diamond Light Source employing silicon double crystal monochromated synchrotron radiation (0.6889 Å) with ω and *ψ* scans at 100(2) K.[5] Data integration and reduction were undertaken with Xia2.[6] Subsequent computations were carried out using the WinGX-32 graphical user interface.[7] Absorption corrections were applied to the data using the AIMLESS[8] tool in the CCP4 suite.[9] The structures were solved by intrinsic phasing using SHELXT[10] then refined and extended with SHELXL.[11] In general, non-hydrogen atoms with occupancies greater than 0.5 were refined anisotropically. Carbon- and nitrogen-bound hydrogen atoms were included in idealised positions and refined using a riding model. Oxygen-bound hydrogen atoms were first located in the difference Fourier map before refinement where possible. Disorder was modelled using standard crystallographic methods including constraints, restraints and rigid bodies where necessary. Crystallographic data along with specific details pertaining to the refinement follow. Crystallographic data have been deposited with the CCDC (2115416 and 2115489).

$(S)_{24}$-Λ_8-**1**

Formula $C_{634}H_{495}F_{192}N_{161}O_{94}S_{32}Zn_{14}$, *M* 17461.98, trigonal, space group R 3 (#146), *a* 46.2635(2), *b* 46.2635(2), *c* 46.8217(5) Å, *γ* 120°, *V* 86787.0(13) Å^3, D_c 1.002 g cm^{-3}, *Z* 3, crystal size 0.020 by 0.015 by 0.012 mm, colour intense purple, habit cube, temperature 100(2) Kelvin, *λ*(Synchrotron) 0.6889 Å, *μ*(Synchrotron) 0.389 mm^{-1}, T(Analytical)$_{min,max}$ 0.9937906689213667, 1.0, $2\theta_{max}$ 51.00, *hkl* range -57 53, -57 57, -56 58, *N* 304735, N_{ind} 77062(R_{merge} 0.0398), N_{obs} 32461(I > 2σ(I)), N_{var} 3391, residuals* $R1(F)$ 0.0596, $wR2(F^2)$ 0.1544, GoF(all) 1.005, $\Delta\rho_{min,max}$ -0.270, 0.419 e$^-$ Å^{-3}.

*$R1 = \Sigma||F_o| - |F_c||/\Sigma|F_o|$ for $F_o > 2\sigma(F_o)$; $wR2 = (\Sigma w(F_o^2 - F_c^2)^2/\Sigma(wF_c^2)^2)^{1/2}$ all

reflections

$w=1/[\sigma^2(F_o^2)+(0.0640P)^2]$ where $P=(F_o^2+2F_c^2)/3$

Specific refinement details:

Crystals of **1**·16NTf$_2$·6H$_2$O·MeCN [+ solvent] were grown by vapor diffusion of diethyl ether into an acetonitrile solution of the complex. The crystals employed immediately lost solvent after removal from the mother liquor but rapid handling prior to quenching in liquid nitrogen and the use of synchrotron radiation enabled data to be collected to ca. 0.8 Å. The complex crystallised in chiral space group *R*3 and the asymmetric unit was found to contain one third of a Zn$_8$L$_6$ assembly plus associated solvents and counterions. The absolute configuration of the structure was confirmed using anomalous dispersion effects and the Flack parameter[12] refined to 0.040(3).

Due to relatively high levels of thermal motion present throughout the structure bond lengths and angles within pairs of chemically identical organic ligand arms were restrained to be similar to each other (SAME). Thermal parameter restraints (SIMU, RIGU) were applied to all atoms except for zinc to facilitate anisotropic refinement. Five of the eight crystallographically unique *tert*-butylmethylamide substituents could be readily resolved but the remaining three were substantially disordered and required extensive restraints to facilitate realistic modelling. The GRADE program[13] was employed using the GRADE Web Server[14] to generate a full set of bond distance and angle restraints (DFIX, DANG, FLAT) for the organic ligand arms, which were then applied to the disordered *tert*-butylmethylamide substituents and where necessary to other parts of the structure showing high degrees of thermal motion. The disordered *tert*-butylmethylamide substituents were modelled isotropically. Even after modelling as disordered over two locations, the thermal parameters of some of these groups and several other outer substituents and tetrafluorophenyl rings remain larger than ideal as a result of significant thermal motion (or dynamic disorder) resulting in a high U_{eq} min/max range for the main residue.

The porphyrin zinc atoms and coordinated water molecules were modelled as disordered over two positions on opposite sides of the porphyrin ring with equal occupancies. The hydrogen atoms of the water molecules were first located in the electron density map and then refined with bond length and angle restraints. Hydrogen bond acceptors were not located for any of the water molecules, presumably due to the highly disordered nature of the surrounding anions and solvents within the structure, giving rise to several B-level Checkcif alerts.

The anions and solvent molecules within the structure show evidence of disorder. One triflimide anion was modelled as disordered over two locations and two further triflimides were modelled with partial occupancy and their occupancies refined. Substantial bond length and thermal parameter restraints were applied to facilitate a reasonable refinement of the anions. Some atoms in the triflimide anions show larger than ideal thermal parameters suggesting further dynamic disorder. Attempts to model further discrete disordered positions of these anions did not improve the model. The hydrogen atoms of an acetonitrile solvent molecule disordered around a special position were not modelled resulting in a Checkcif alert for an apparent singly bonded carbon.

There is a significant amount of void volume in the lattice containing smeared electron density from disordered solvent and ca. 2.8 anions per Zn_8L_6 assembly (assigned to triflimide in the formula). Consequently the SQUEEZE[15] function of PLATON[16] was employed to remove the contribution of the electron density associated with these remaining anions and further highly disordered solvent, which gave a potential solvent accessible void of 38966 Å^3 per unit cell (a total of approximately 9949 electrons). Diffuse solvent molecules could not be assigned to acetonitrile or diethyl ether and were therefore not included in the formula. Consequently, the molecular weight and density given above are underestimated.

CheckCIF gives 1 A level alert due to isotropic modelling of the disordered substituents and 11 B levels alerts, mostly arising from water molecules missing hydrogen bond acceptors as well as the acetonitrile molecule for which hydrogen

atoms were not modelled and the presence of a high level of thermal motion (or dynamic disorder) in parts of the structure as described above.

(S)$_{24}$-Λ_8-**2**

Formula $C_{632}H_{490.50}Co_8F_{192}N_{160}O_{93.25}S_{32}Zn_6$, M 17355.90, trigonal, space group R 3 (#146), a 46.2635(2), b 46.2635(2), c 46.8217(5) Å, γ 120°, V 86787.0(13) Å^3, D_c 0.996 g cm^{-3}, Z 3, crystal size 0.150 by 0.130 by 0.120 mm, colour intense purple, habit cube, temperature 100(2) Kelvin, λ(Synchrotron) 0.6889 Å, μ(Synchrotron) 0.350 mm^{-1}, T(Analytical)$_{min,max}$ 0.994925685280335, 1.0, $2\theta_{max}$ 36.03, hkl range -41 41, -41 41, -42 42, N 72590, N_{ind} 28908(R_{merge} 0.0281), N_{obs} 13669(I > 2σ(I)), N_{var} 3350, residuals* $R1(F)$ 0.0826, $wR2(F^2)$ 0.2321, GoF(all) 0.997, $\Delta\rho_{min,max}$ -0.221, 0.377 e$^-$ Å^{-3}.

*$R1 = \Sigma||F_o| - |F_c||/\Sigma|F_o|$ for $F_o > 2\sigma(F_o)$; $wR2 = (\Sigma w(F_o^2 - F_c^2)^2/\Sigma(wF_c^2)^2)^{1/2}$ all reflections

$w=1/[\sigma^2(F_o^2)+(0.1350P)^2]$ where $P=(F_o^2+2F_c^2)/3$

Specific refinement details:

Crystals of **2**·16NTf$_2$·5.25H$_2$O [+ solvent] were grown by vapor diffusion of diethyl ether into an acetonitrile solution of the complex. Despite appearing visually of good quality, the crystals employed proved to be weakly diffracting and suffered immediate solvent loss. Rapid handling prior to quenching in liquid nitrogen was required to collect data. Despite these measures and the use of synchrotron radiation few reflections were observed past 1.1 Å resolution and the data were trimmed accordingly. Nevertheless, the quality of the data is far more than sufficient to establish the connectivity of the structure. The complex crystallised in chiral space group $R3$ and was approximately isomorphous to the structure of **1**·16NTf$_2$·6H$_2$O·MeCN described above apart from minor differences related to the

treatment of disorder. An initial solution was thus obtained using the structure of the Zn(II)-cornered analogue. The absolute configuration of the structure was confirmed using anomalous dispersion effects and the Flack parameter[12] refined to 0.087(7). The small deviation from zero can be explained by the high degree of disorder and thermal motion within the structure.

Due to the less than ideal resolution and high levels of thermal motion present throughout the structure the restraints, calculated using the GRADE program[13] as described above were applied to all of the ligand arms. Thermal parameter restraints (SIMU, RIGU) were applied to all atoms except for zinc and cobalt to facilitate anisotropic refinement. Five of the eight crystallographically unique *tert*-butylmethylamide substituents were modelled as disordered over two locations and the disordered substituents were modelled isotropically.

The porphyrin zinc atoms were disordered over two positions on opposite sides of the porphyrin ring with equal occupancies and some of their coordinated water molecules were modelled with partial occupancy. The hydrogen atoms of the water molecules could not be located in the electron density map and were not included in the model.

The anions and solvent molecules within the structure show evidence of disorder. One triflimide anion was modelled as disordered over two locations and the occupancies of the other located triflimides were refined; any additional minor occupancy disorder positions of these anions could not be resolved. Substantial bond length and thermal parameter restraints were applied to facilitate a reasonable refinement of the anions. Even with these restraints some atoms in the triflimide anions show larger than ideal thermal parameters suggesting further dynamic disorder.

Further reflecting the solvent loss there is a significant amount of void volume in the lattice containing smeared electron density from disordered solvent and ca. 5.2 anions per Co_8L_6 assembly (assigned to triflimide in the formula). Consequently, the SQUEEZE[15] unction of PLATON[16] was employed to remove the contribution of the electron density associated with these remaining anions and further highly disordered solvent, which gave a potential solvent accessible void of 36565 Å^3 per unit cell (a total of approximately 8395 electrons). Diffuse solvent molecules could not be

assigned to acetonitrile or diethyl ether and were therefore not included in the formula. Consequently, the molecular weight and density given above are underestimated. CheckCIF gives 2A level alert and 4B levels alerts. These alerts arise from the limited resolution, isotropic modelling of the disordered substituents and anions showing thermal motion/dynamic disorder.

8 Investigation of the Monomeric ZnL_3 Complex

Synthesis the monomeric chiral pyridyl-triazole ligand:

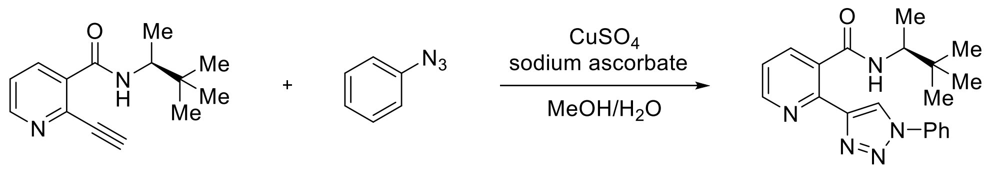

S4 **S6** **S7**

85%

To a solution of phenyl azide (1.0 mL, 1.0 mmol, 1.0 equiv, 0.5 M in 2-MeTHF) in MeOH/H_2O (3/2, 10 mL) was added $CuSO_4{\cdot}5H_2O$ (37.5 mg, 0.15 mmol, 0.15 equiv), sodium ascorbate (59.4 mg, 0.30 mmol, 0.30 equiv) and **S4** (230.3 mg, 1.0 mmol, 1.0 equiv). The reaction mixture was stirred at room temperature for 16 hours under nitrogen. Brine (50 mL) was then added, and the suspension was extracted with CH_2Cl_2 (3 × 50 mL). The combined organic phases were dried over anhydrous Na_2SO_4, filtered, and the solvents evaporated under reduced pressure. Purification by flash column chromatography on silica gel using hexane/EtOAc = 1/4 as eluent afforded **S7** as a pale yellow solid (298 mg, 85%).

R_f = 0.45 (hexane/EtOAc = 1/4). **^{1}H NMR** (400 MHz, CD_3CN): δ 0.89 (s, 9H), 1.08 (d, *J* = 6.7 Hz, 3H), 3.92–4.02 (m, 1H), 6.60 (d, *J* = 8.8 Hz, 1H), 7.36–7.42 (m, 1H), 7.48–7.55 (m, 1H), 7.57–7.64 (m, 2H), 7.82 (dd, *J* = 7.7, 1.7 Hz, 1H), 7.85–7.88 (m, 2H), 8.65–8.71 (m, 2H) ppm. **^{13}C NMR** (100 MHz, CD_3CN): δ 15.7, 26.6, 35.1, 54.4, 121.6, 123.2, 123.7, 129.9, 130.8, 133.6, 137.2, 138.0, 147.0, 148.6, 150.8, 168.5 ppm

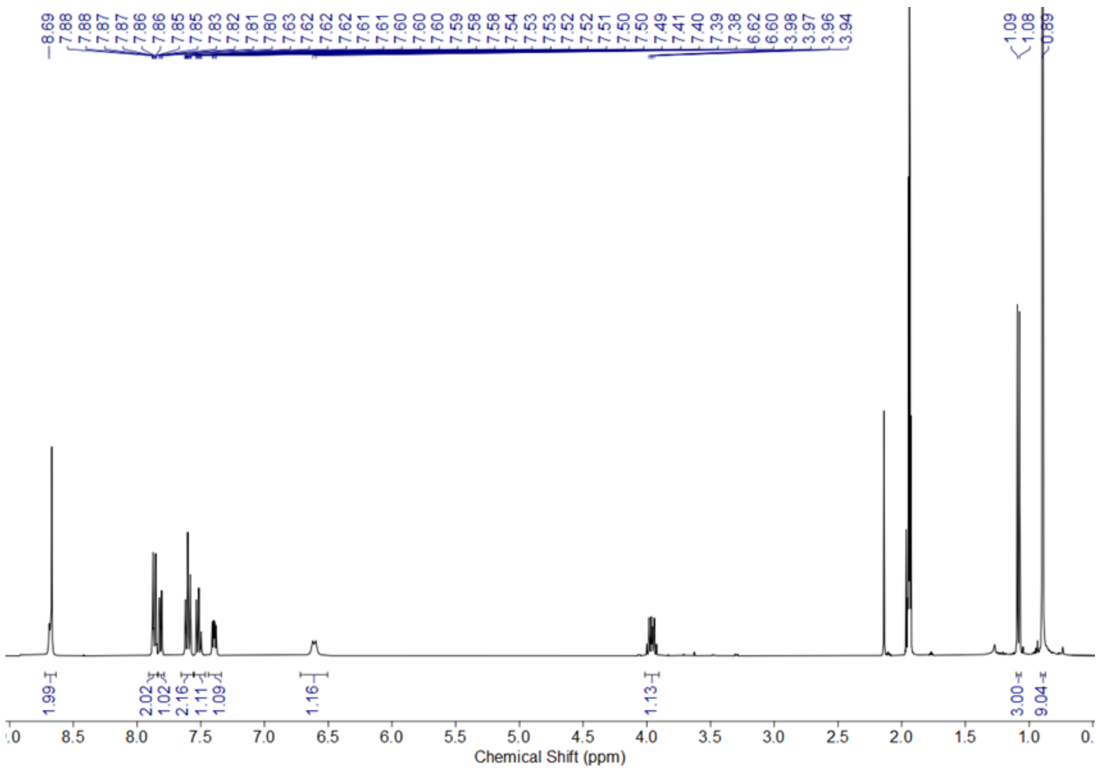

Figure S75. ^{1}H NMR spectrum of **S7** (400 MHz, CD_3CN, 25 °C).

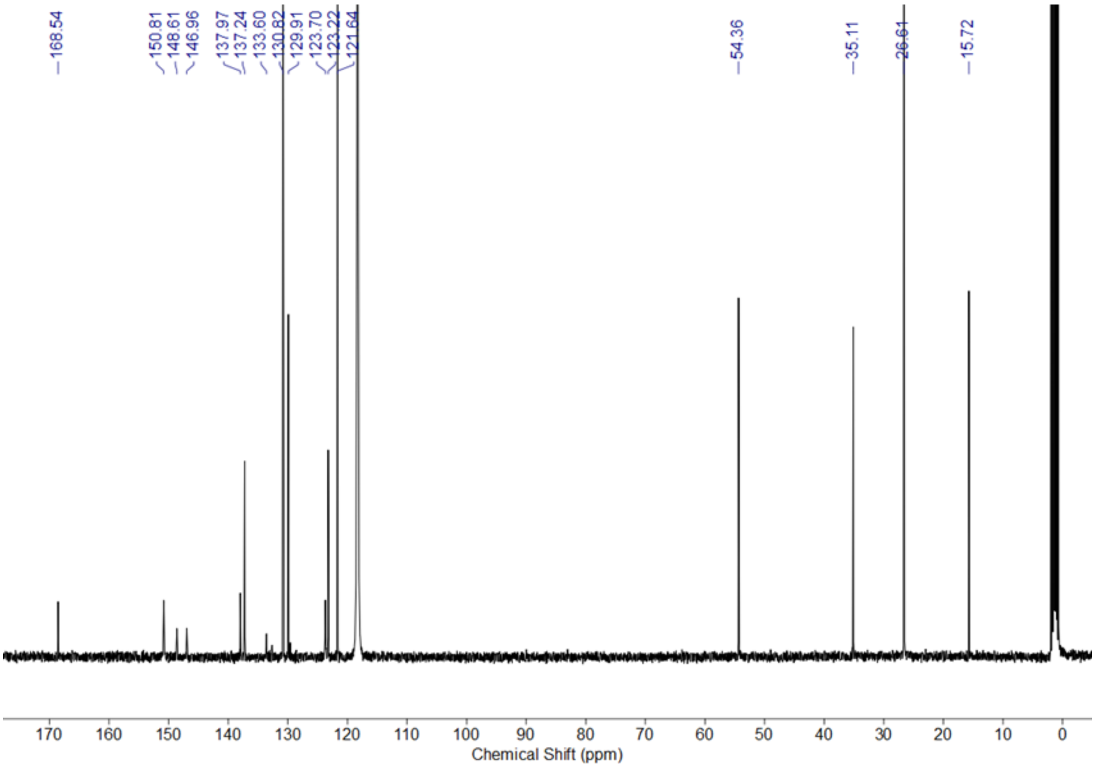

Figure S76. ^{13}C NMR spectrum of **S7** (100 MHz, CD_3CN, 25 °C).

Self-assembly of S7 with $Zn(NTf_2)_2$:

Ligand **S7** (2.10 mg, 6.0 μmol, 3.0 equiv) and $Zn(NTf_2)_2$ (1.25 mg, 2.0 μmol, 1.0 equiv) were combined in the corresponding deuterated solvents (0.5 mL) in NMR tubes. The reaction mixtures were heated at 70 °C for 2 hours. After cooling down to room temperature, VTA ^1H NMR spectra were measured.

The separated H_a signals at low temperatures allowed us to determine the diastereomeric ratio of the ZnL_3 complex in both CD_3CN and CD_3NO_2. In both solvents, the d.r. was determined to be 1.3:1, and the solvent-dependent stereochemical inversion phenomenon was not observed. Due to the low diastereoselectivity, weak Cotton effects were observed in CD spectra of ZnL_3 in MeCN; CD and UV spectra of ZnL_3 could not be measured in $MeNO_2$ due to the strong background signal from $MeNO_2$ (200–350 nm).

The above results indicated that the energy difference between the cage diastereomers emerges as a consequence of higher-order assembly.

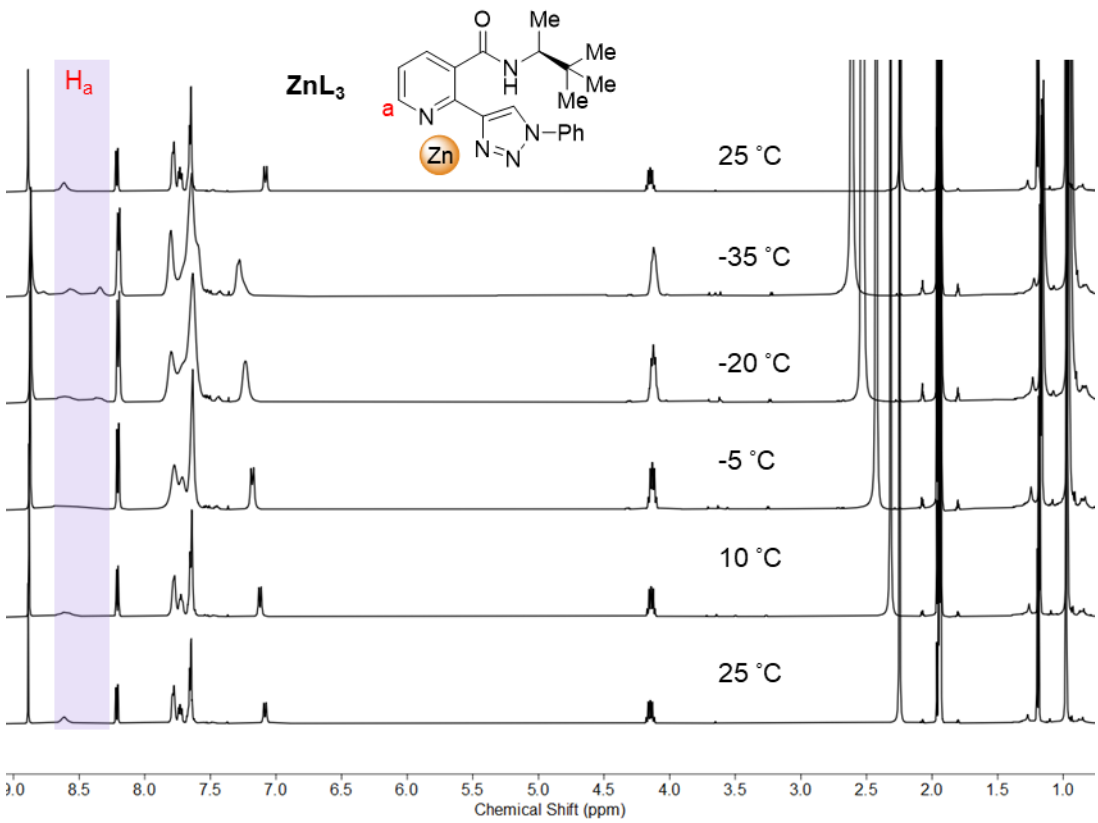

Figure S77. ^{1}H NMR spectra (500 MHz, CD_3CN) of ZnL_3 at different temperatures.

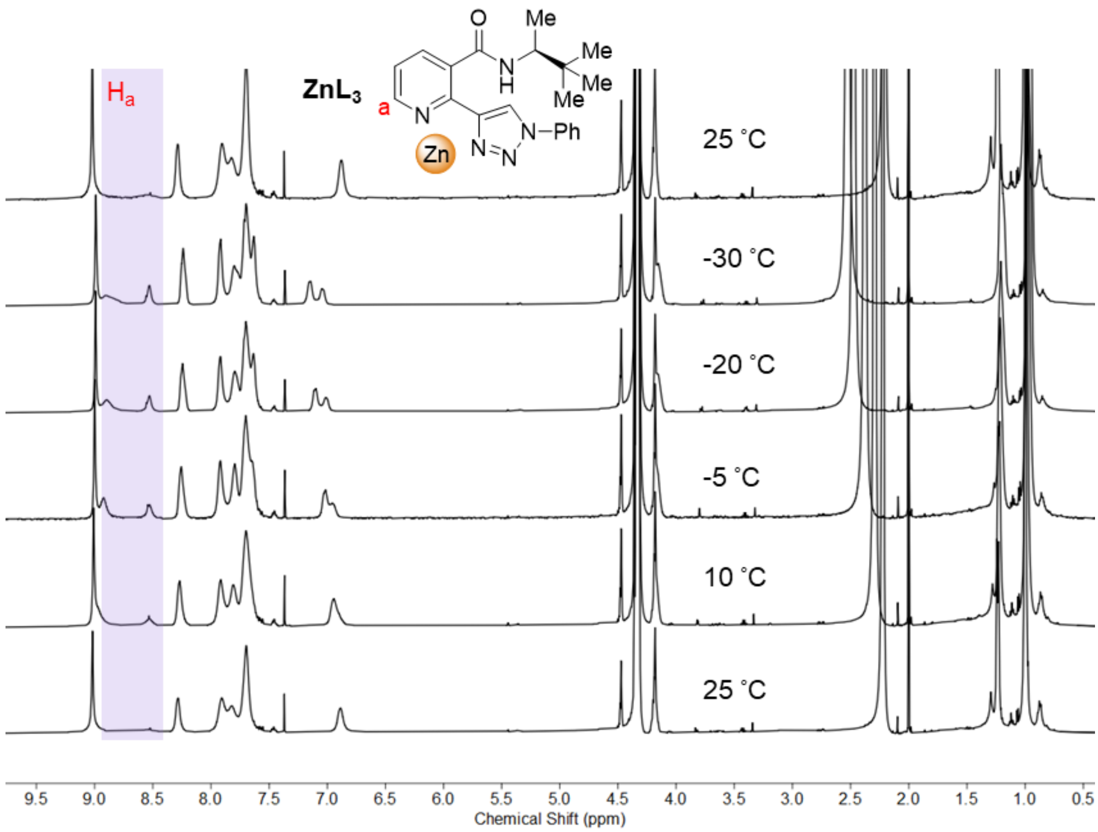

Figure S78. ^{1}H NMR spectra (500 MHz, CD_3NO_2) of ZnL_3 at different temperatures.

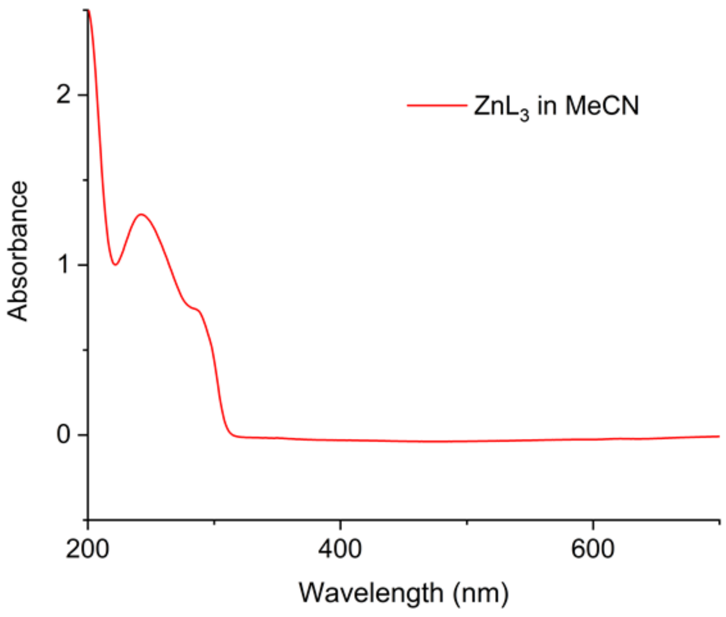

Figure S79. UV/Vis spectrum of ZnL_3 in MeCN.

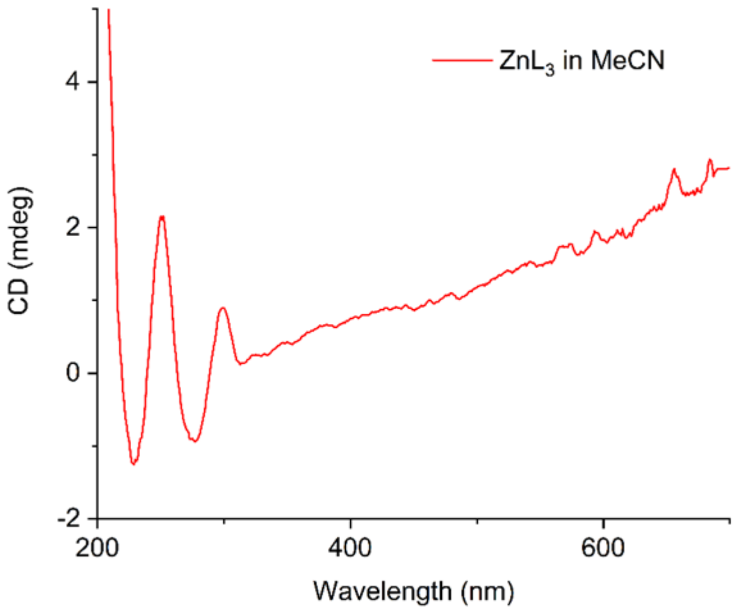

Figure S80. CD spectrum of ZnL_3 in MeCN.

9 References

1. Golf, H. R. A.; Reissig, H.-U.; Wiehe, A., Regioselective Nucleophilic Aromatic Substitution Reaction of *meso*-Pentafluorophenyl-Substituted Porphyrinoids with Alcohols. *Eur. J. Org. Chem*. **2015**, 1548.

2. Brenner, W.; Ronson, T. K.; Nitschke, J. R., Separation and Selective Formation of Fullerene Adducts within an $M^{II}_8L_6$ Cage. *J. Am. Chem. Soc.* **2017**, *139*, 75.

3. Maglic, J. B.; Lavendomme, R., An Easy-to-Use Program to Calculate Various Volumes and Surface Areas of Chemical Structures and Identify Cavities. doi.org/10.33774/chemrxiv-2021-dss1j.

4. Kleywegt, G. J.; Jones, T. A., Detection, Delineation, Measurement and Display of Cavities in Macromolecular Structures. *Acta Crystallogr., Sect. D: Biol. Crystallogr.* **1994**, *D50*, 178.

5. Allan, D.; Nowell, H.; Barnett, S.; Warren, M.; Wilcox, A.; Christensen, J.; Saunders, L.; Peach, A.; Hooper, M.; Zaja, L.; Patel, S.; Cahill, L.; Marshall, R.; Trimnell, S.; Foster, A.; Bates, T.; Lay, S.; Williams, M.; Hathaway, P.; Winter, G.; Gerstel, M.; Wooley, R., A Novel Dual Air-Bearing Fixed-χ Diffractometer for Small-Molecule Single-Crystal X-ray Diffraction on Beamline I19 at Diamond Light Source. *Crystals* **2017**, *7*, 336.

6. (a) Collaborative Computational Project, N., The CCP4 suite: Programs for Protein Crystallography. *Acta Cryst.* **1994**, *D50*, 760. (b) Evans, P., Scaling and Assessment of Data Quality. Acta Cryst. 2006, D62, 72; (c) Winter, G., Xia2: An Expert System for Macromolecular Crystallography Data Reduction. *J. Appl. Crystallogr.* **2010**, *43*, 186.

7. Farrugia, L., WinGX and ORTEP for Windows: an update. *J. Appl. Crystallogr.* **2012**, *45*, 849.

8. Evans, P. R.; Murshudov, G. N., How good are my data and what is the resolution? *Acta Cryst.* **2013**, *D69*, 1204.

9. Winn, M. D.; Ballard, C. C.; Cowtan, K. D.; Dodson, E. J.; Emsley, P.; Evans, P. R.; Keegan, R. M.; Krissinel, E. B.; Leslie, A. G. W.; McCoy, A.; McNicholas, S. J.; Murshudov, G. N.; Pannu, N. S.; Potterton, E. A.; Powell, H. R.; Read, R. J.; Vagin, A.; Wilson, K. S., Overview of the CCP4 Suite and Current Developments. *Acta Cryst.* **2011**, *D67*, 235.

10. Sheldrick, G., SHELXT-Integrated Space-Group and Crystal-Structure Determination. *Acta. Cryst.* **2015**, *A71*, 3.

11. Sheldrick, G. M., Crystal Structure Refinement with SHELXL. *Acta. Cryst.* **2015**, *C71*, 3.

12. Flack, H. D., Chiral and Achiral Crystal Structures. *Helv. Chim. Acta* **2003,** *86*, 905.

13. Bricogne, G.; Blanc, E.; Brandle, M.; Flensburg, C.; Keller, P.; Paciorek, W.; Roversi, P.; Sharff, A.; Smart, O. S.; Vonrhein, C.; Womack, T. O., *BUSTER*. 2.11.2 ed.; Global Phasing Ltd.: Cambridge, United Kingdom, 2011.

14. Smart, O. S.; Womack, T. O., *Grade Web Server*. Global Phasing Ltd., 2014.

15. van der Sluis, P.; Spek, A. L., BYPASS: An Effective Method for the Refinement of Crystal Structures Containing Disordered Solvent Regions. *Acta Cryst.* **1990,** *A46*, 194.

16. Spek, A. L., *PLATON: A Multipurpose Crystallographic Tool*. Utrecht University: Utrecht, The Netherlands, 2008.
